# How parents leverage guilt and pride: A comparison of parental guilt and pride induction in Hong Kong and the United States

**DOI:** 10.1111/jora.70107

**Published:** 2025-12-10

**Authors:** Natalie Wong, Wendy M. Rote

**Affiliations:** ^1^ Department of Educational Psychology The Chinese University of Hong Kong Sha Tin Hong Kong; ^2^ Department of Psychology University of South Florida St. Petersburg campus St. Petersburg Florida USA

**Keywords:** cross‐cultural, domain‐differentiated, guilt induction, parenting, pride induction, self‐conscious emotions

## Abstract

Parental socialization of self‐conscious emotions is crucial in shaping children's behaviors and moral development. While research has frequently examined the socialization of negative emotions like guilt, the socialization of positive emotions such as pride remains understudied. This gap limits our understanding of the outcomes and cultural differences in parental socialization of self‐conscious emotions. Additionally, current research often fails to consider domain differences in the socialization of self‐conscious emotions despite evidence indicating varying levels of perceived legitimacy and acceptance by adolescents, leading to a restricted understanding of their connection with parent–child relationships. Our study investigated the association between adolescents' perceptions of parents' pride and guilt induction across different domains (i.e., moral, conventional, prudential, and personal) and adolescents' perceptions of parent–child relationship quality in the United States (*N* = 142) and Hong Kong (*N* = 124). The domain‐differentiated guilt and pride induction scales demonstrated scalar invariance, indicating consistent meaning and structure across both cultural groups. Results revealed both cultural similarities and differences. In both cultures, guilt induction on personal issues was negatively associated with relationship quality, while perceived levels of pride induction across all issue types were positively associated with relationship satisfaction. However, perceived levels of guilt induction on other issues were negatively associated with relationship quality only in the US sample. The results suggest cultural similarities and differences in how adolescents perceive their parents' use of guilt and pride induction. Our study emphasizes the significance of examining the socialization of positive self‐evaluation emotions, like pride, and the need to differentiate between various domains in emotion socialization research.

## INTRODUCTION

Parental socialization of emotions plays a critical role in shaping adolescent behavior and adolescent–parent relationships. Theorists have emphasized parents' role in socializing negative self‐conscious emotions such as guilt (Baumeister, 1998; Rote et al., 2024) and positive self‐conscious emotions such as pride (Hagan et al., 2021; Leary, 2013; Tangney, 1999) during child and adolescent development. However, there is a notable imbalance in empirical research. While empirical research has extensively explored parental induction of negative self‐conscious emotions (e.g., Rote et al., 2022; Rote & Smetana, 2017; see also Choe et al., 2023 for a detailed review), considerably less attention has been given to parental pride induction (see discussion in Hagan et al., 2021). This disparity exists despite both emotions being theorized as significant motivators of behavior, highlighting the need to consider the induction of both positive and negative self‐conscious emotions simultaneously.

Moreover, existing studies on parental induction of positive and negative self‐conscious emotions have primarily focused on their effects on adolescents' behaviors or experiences of these emotions, largely neglecting their impact on parent–child relationships. This oversight represents a significant gap in the literature, especially considering that research in related areas of parenting, such as psychological control (Choe et al., 2023, review) and conditional regard (Haines & Schutte, 2023, meta‐analysis), suggests that such induction practices could potentially undermine adolescent–parent relationship quality.

Among the various types of self‐conscious emotions (i.e., guilt, shame, embarrassment, and pride; Tangney, 1999), the comparison between parental guilt and pride induction is particularly interesting in the context of adolescent–parent relationship quality. Unlike the induction of shame and embarrassment, which is generally found to be negatively related to adjustment (e.g., Smiley et al., 2020), both guilt induction (e.g., Rote et al., 2022; Wong & Konishi, 2023) and pride induction (e.g., Katzir et al., 2010) have sometimes been associated with adaptive functioning. Given the potential benefits of pride and guilt induction in promoting positive adolescent development, it becomes crucial to examine their potential cost on adolescent–parent relationship quality.

To address these gaps, the present study investigates domain‐differentiated parental pride induction and guilt induction, as conceptualized by Social‐Cognitive Domain Theory (Smetana, 2024), and their associations with adolescents' perceived relationship quality with their parents. By examining both positive and negative self‐conscious emotion induction, the present study aims to provide a more comprehensive understanding of their relational consequences. Importantly, pride and guilt are suggested to be valued differently across cultures, with individualistic cultures tending to emphasize pride and collectivistic cultures often prioritizing guilt (Furukawa et al., 2012). These cultural differences may shape the pattern of parental pride and guilt induction and modulate how they influence adolescent–parent relationships across cultural contexts. Given this potential cultural variation, this study compares how these associations may vary between adolescents from the United States and Hong Kong, offering insight into the cultural differences in the association of parent emotion socialization practices and adolescent–parent relationships.

### Parental guilt induction and adolescent–parent relationship

Research has shown that parents' use of guilt induction negatively affects adolescent–parent relationships in both Eastern and Western cultures. In a study on cultural context in parenting, Fung and Lau (2012) compared the association between relational induction and adolescent–parent relationships among European‐American and Hong Kong‐Chinese families. Although European‐American families showed a stronger link between guilt induction and perceptions of parental rejection compared with Hong Kong‐Chinese families, the negative relationship was consistent across both cultural contexts (Fung & Lau, 2012). This finding aligns with research on related parenting practices, such as psychological control, which involves emotional manipulation (Barber et al., 2012; Soenens & Vansteenkiste, 2010), and conditional negative regard, which involves withdrawing affection to reduce undesirable behavior (Roth et al., 2009). These practices have been associated with poor parent–child relationships (Kanat‐Maymon et al., 2016) and increased attachment anxiety in adulthood (Choe et al., 2021). Together, these findings underscore the importance of understanding the relational costs of inducing negative self‐conscious emotions.

However, to fully grasp the implications of guilt induction, it is essential to consider the specific domains in which it occurs. Social‐Cognitive Domain Theory (Smetana, 2024) provides a framework for understanding how the context of guilt induction might influence its impact on adolescent–parent relationships. This theory distinguishes between different types of social knowledge: (1) Moral issues: behaviors that affect others' rights or well‐being; (2) Conventional issues: societal rules and norms; (3) Prudential issues: behaviors that primarily affect one's own safety or health; (4) Personal issues: matters of individual choice and identity. Individuals make these domain distinctions across cultures (Smetana, 2024), and although there is some variation, adolescents consistently assign certain issues to specific domains regardless of cultural contexts (Helwig, 2006). For instance, adolescents in the US, Japan, the Philippines, and Chile all conceptualize their choice of friends, dress, and free time activities as mainly personal issues and smoking, drug, and alcohol use as prudential issues (Darling et al., 2005; Hasebe et al., 2004). Adolescents in Shenzhen and Hong Kong similarly justify conflicts with parents about free time activities and interpersonal behaviors in terms of maintaining personal jurisdiction (Yau & Smetana, 2003). Thus, even when there are mean level differences across cultures in parental control (e.g., Darling et al., 2005), youth tend to draw similar distinctions in the types of behaviors over which they should have more or less autonomy.

Relatedly, research applying this framework to parental control and emotion induction has revealed that adolescents' feelings about these practices vary significantly across different domains. For instance, studies in both Eastern (Smetana et al., 2021) and Western (Rote & Smetana, 2017) cultures have found that adolescents perceive parental guilt and shame induction for moral and prudential transgressions as more acceptable and less psychologically controlling than when applied to personal issues. Findings on guilt and shame induction using the domain‐differentiated approach align with recent reviews of parental psychological control, which highlight intrusiveness (on top of emotional manipulation) as one of the two key factors in its negative impact on adolescents' social development and parent–child relationships (Choe et al., 2023). The perceived legitimacy of parental intervention (Rote & Smetana, 2017) and whether adolescents' individuality is respected (Barber et al., 2012) play crucial roles in determining the effects of such practices.

Therefore, to gain a more nuanced understanding of how parental guilt induction affects the quality of adolescent–parent relationships, it is essential to consider the specific domains in which this induction occurs. This approach can uncover significant variations in parents' use of guilt induction across different domains of social knowledge, as well as the potential costs associated with these practices for adolescent–parent relationships.

### Parental pride induction and adolescent–parent relationship

While pride has been linked to positive outcomes such as increased prosocial behaviors (Hart & Matsuba, 2007; Tracy & Robins, 2007; Verbeke et al., 2004; Weidman et al., 2018), self‐esteem (Tracy et al., 2023) and goal‐attainment abilities (Katzir et al., 2010), research on parents' role in socializing pride remains limited. Notably, emerging research has explored parental pride induction, but existing studies have not examined its links to parent–child relationships (Hagan et al., 2021). Instead, much of the literature has focused on related concepts, such as parents' use of praise to encourage desirable behaviors, and conditional positive regard, which involves providing affirmation only when children meet expectations. Understanding the relational impact of parental pride induction requires exploring its connection to and distinction from these related constructs. While praise is likely a primary way parents induce feelings of pride in their children, pride induction encompasses a broader range of behaviors, including nonverbal expressions (e.g., smiling, showing excitement) and indirect actions (e.g., documenting and sharing a child's accomplishments with others). Moreover, the concept of pride induction shifts focus from parental action alone (e.g., praise) to the child's emotional response, allowing for the possibility that praise might not always make a child feel proud. Similarly, conditional positive regard and pride induction differ in the range of emotions they evoke: the latter focuses on pride, while the former also evokes feelings of being loved. Yet, both constructs elicit positive self‐conscious emotions such as pride and esteem. As such, research on conditional positive regard also provides a valuable foundation for understanding how pride induction may function in parent–child relationships.

It may be tempting to assume that parental pride induction enhances adolescent–parent relationships. However, insights from research on conditional positive regard suggest a more nuanced picture. Kanat‐Maymon et al. (2016) found that conditional positive regard, which depends on children's desirable behaviors or achievements, is linked to poorer parent–child relationship quality (Kanat‐Maymon et al., 2016). Similarly, Israeli‐Halevi et al. (2015) indicated that while mothers' conditional positive regard correlates with their own contingent self‐esteem, it does not translate into warmth and support for their children. This raises questions about whether parental pride induction, often contingent on children's behaviors, is beneficial for parent–child relationships (Israeli‐Halevi et al., 2015).

To fully grasp the relational implications of parental pride induction, it is important to distinguish it from conditional positive regard. Conditional positive regard involves the contingent provision of love, acceptance, and affection (Haines & Schutte, 2023). The relational costs of conditional positive regard stem from its frustration of relatedness needs associated with *unconditional* acceptance from significant others (Ryan & Deci, 2018), a link supported by a recent meta‐analysis (Haines & Schutte, 2023). However, unlike individuals' hopes for unconditional acceptance from close others, expressions of pride from others are typically expected to be conditional. Research on parental praise illuminates this distinction. Lee et al. (2016) found that both parents' over‐praising and under‐praising negatively affect children's academic performance and mental health, while appropriate praise has beneficial effects. This suggests that pride might be expected to be conditionally bestowed by parents, and such accurate positive appraisal, along with the resulting feelings of pride, could be viewed as supportive and be beneficial for the relationship. Corroborating this argument, studies indicate that parents' appropriate recognition of their children's academic achievements can enhance parent–child relationship quality. For instance, Li, Song, Zhou, Gu et al. (2024) found that parents' success‐oriented responses proportional to their children's academic achievements (e.g., “My parents would be very proud (when I succeed)”) were associated with stronger youth‐reported parent–child bonds. This distinction underscores the importance of studying pride induction as a unique construct, as it isolates the mechanisms by which parents influence children's self‐conscious emotions, separate from the broader affirmations of affection that are uniquely tied to unconditional acceptance.

In summary, evidence indicates that appropriate parental praise is positively associated with parent–child relationship quality, providing initial insights into how the broader construct of pride induction might relate to parent–child relations. However, a critical gap in the literature remains: the lack of domain differentiation. Adolescents perceive certain domains as more legitimately regulated by parents (Smetana, 2024), meaning that pride induction in these domains may enhance parent–child relationships, while pride induction in less legitimately regulated domains could be perceived as intrusive or unwarranted and, therefore, less beneficial. To deepen our understanding of how pride induction across different domains may impact adolescent–parent relationships, this study will adopt a domain‐differentiated approach similar to our approach to parental guilt induction.

### Parental guilt and pride induction and cultural differences

Culture may influence the frequency and pattern of parental pride and guilt induction, as well as their association with parent–child relationship quality. While direct cross‐cultural comparisons of parental pride and guilt induction are limited, existing research suggests potential cultural differences warrant further exploration. Cultural differences in the value placed on different self‐conscious emotions may influence parents' use of pride and guilt induction; individualistic cultures tend to emphasize pride, while collectivistic cultures often prioritize guilt (Furukawa et al., 2012). This cultural difference could affect the frequency with which parents employ these strategies. However, empirical findings are mixed. Some studies suggest that parents in Eastern/collectivistic cultures use more guilt and shame induction than those in Western/individualistic cultures (Chao & Aque, 2009; Fung & Lau, 2012). Other research presents mixed results (e.g., Lansford et al., 2018) or finds no significant cultural differences in negative parental control practices (e.g., Huang & Lamb, 2013). While research on parental pride induction is scarce, studies on praise indicate that Western parents use praise more frequently than Eastern parents (e.g., Huang & Lamb, 2013). However, research in this area is limited, and many existing studies failed to report results related to measurement invariance, a critical factor in ensuring valid cross‐cultural comparisons. This significant gap in research methodology underscores the pressing need for more rigorous cross‐cultural studies in this field.

Cultural beliefs may also influence parents' motivations for using pride and guilt induction, potentially affecting usage patterns. In collectivistic cultures, both guilt (e.g., Fung & Lau, 2012) and pride induction (in the form of praising; e.g., Wang et al., 2008) are generally viewed as acceptable ways to promote adherence to parents' expectations. Consequently, the use of pride and guilt induction might be positively correlated in these contexts. In Chinese cultures, this positive association is likely to be most pronounced in domains concerning collective welfare (moral), societal norms (conventional), and individual well‐being (prudential), because Chinese parenting philosophies emphasize guiding children toward behaviors that align with collectivistic values while simultaneously protecting them from potentially harmful influences (Chao, 2000). In contrast, individualistic cultures often prioritize nurturing children's self‐esteem (Miller et al., 2002), which may result in a different pattern of emotion induction. In these societies, guilt induction practices are perceived as more problematic and associated with negative perceptions of the child (e.g., Rudy & Halgunseth, 2005) while pride induction (such as the use of praise) is viewed more positively and used to promote independence (e.g., Wang et al., 2008). As such, we could expect in individualistic cultures the association between parental guilt induction and pride induction to be negative, particularly in the personal domain—an area considered crucial for the development of autonomy (Nucci, 2001). Empirical evidence lends support to these hypothesized cultural differences. Studies have found that Chinese parents' success‐oriented (i.e., highlighting success) and failure‐oriented (i.e., highlighting failure) responses to their children's academic performance are positively correlated (e.g., Li, Song, & Zhou, 2024), while in Western cultures, parents' praise and criticism of their children's achievements tend to be negatively correlated (e.g., Gunderson et al., 2018).

In terms of culture's influence on parent–child relationships, the relative emphasis on group versus individual interests may modulate the effects of parental pride and guilt induction. The emphasis on group harmony in many collectivistic cultures could potentially lead to greater acceptance of guilt induction, potentially mitigating its adverse effects on parent–child relationships. Some researchers argue that guilt induction in collectivist cultures helps children consider the relational consequences of their actions, promoting self‐regulation and collectivist values (Fung & Lau, 2012). Evidence suggests that Asian children are less likely to interpret relational forms of induction as parental rejection compared to their European–American counterparts (Fung et al., 2017). However, further research is needed to directly compare the use of guilt induction between parents from collectivistic and individualistic cultures. Research comparing parental pride induction across cultures is almost nonexistent. While we speculate that pride induction might be related to better parent–child relationship quality in light of previous findings on the positive association between praise and parent–child relationships (Li, Song, Zhou, Gu et al., 2024), there is no research on potential cultural differences in this association.

In sum, while existing research suggests cultural differences in the pattern of parental guilt and pride induction and their association with parent–child relationship quality (especially between Eastern collectivistic and Western individualistic cultures), the field lacks direct cross‐cultural comparisons of these parenting strategies. Our study aims to address this gap in the literature.

### The present study and hypotheses

Our present study aims to address three significant gaps in the research: (1) the lack of investigation into parental pride induction within the context of self‐conscious emotions, (2) the insufficient exploration of the relational consequences of parental induction of self‐conscious emotions, and (3) the absence of a domain‐differentiated approach in studying these phenomena. To achieve these objectives, we collected adolescent reports on domain‐differentiated parental guilt induction, domain‐differentiated parental pride induction, and the quality of adolescent–parent relationships. We utilized a late adolescent sample, asking youth to reflect on parenting practices “while they were growing up,” as children do not develop fully mature understandings of self‐conscious emotions (i.e., their relations to the self and identity) until middle adolescence (Harris et al., 1987; Hart & Matsuba, 2007), and parenting practices during adolescence have persistent effects on the parent–child relationship into emerging adulthood (Aquilino, 1997). Additionally, to explore potential cultural differences, we compared adolescent reports from the United States and Hong Kong. These cultures were selected due to their distinct parenting practices and beliefs, reflecting individualistic and collectivistic orientations, as highlighted by Fung and Lau (2012). Finally, we chose to focus on adolescents' *perceptions* of parental guilt and pride induction rather than specific actions of parents. Individual, cultural, and relational factors can alter youth interpretations of parenting behaviors, including guilt induction (Chao & Aque, 2009; Rote et al., 2021), and it is these subjective experiences (i.e., their “functional significance”), rather than the objective parenting practice, that most strongly predict youth adjustment and relational outcomes (e.g., Soenens et al., 2015). Based on the extant literature regarding the links between guilt induction and parent–adolescent relationships across cultures, we hypothesize:

Perceived guilt induction is negatively related to adolescent–parent relationship quality.

The negative association between perceived guilt induction and adolescent–parent relationship quality is more pronounced in topics perceived as not legitimately regulated by parents (i.e., personal domain).

The negative association between perceived guilt induction and adolescent–parent relationship quality is stronger among US participants.

Drawing from research related to the association between pride induction and parent–adolescent relationships, we posit:

Perceived pride induction is positively related to adolescent–parent relationship quality.

Due to the paucity of research, the potential variations in the association between pride induction and adolescent–parent relationship quality across different social domains or cultures remain unclear. As such, this aspect of our investigation is exploratory in nature.

Regarding cultural differences in parental guilt and pride induction, we hypothesize:

Perceived parental pride induction and guilt induction are positively correlated in the Hong Kong‐Chinese sample, particularly in moral, conventional, and prudential domains.

Perceived parental pride induction and guilt induction are negatively correlated in the U.S. sample, particularly in the personal domain.

Given the mixed findings and limited research on the frequency of parental pride and guilt induction between cultures, this aspect of our study will also be exploratory in nature.

## METHOD

### Participants

Our final sample included 266 late adolescents (97.3% between the ages of 18 and 25), of which 142 were from the United States (*M*_age_ = 20.2 years, 76.8% female, 2.8% nonbinary/third gender) and 124 were from Hong Kong (*M*_age_ = 20.9 years, 70.2% female, 0.8% nonbinary/third gender; four participants chose not to disclose their gender). Out of the 140 adolescents who took part in the study in Hong Kong, 16 were excluded from the analysis. Nine of them completed less than 50% of the questionnaire, and seven failed the attention check items. A majority of the participants from the United States identified as White (71.1%), followed by Black (7.0%), Asian (5.6%), or another ethnicity (6.3%). Forty‐six participants (32.4%) from the United States were identified as Hispanic. Three participants from the United States chose not to disclose their race (2.1%). Most participants from Hong Kong were identified as Asian (88.7%). The remaining 11.3% included participants who were identified as White (1.6%), an ethnicity other than Asian, Black, or White (4.0%), and seven participants who chose not to disclose their race (5.6%); no participants from Hong Kong identified as Hispanic. Regarding parents' highest education level, most participants in the United States had parents who attended some college or more (mother: 71.1%, father: 64.1%), while most participants in Hong Kong had parents who attended some college or less (mother: 70.2%, father: 64.5%). The most reported family income level was between $25,000 and $49,999 USD annually for participants from the United States (18.3%) and Hong Kong (34.7%).

### Procedure

This convenience sample was recruited online via university platforms and mass mailings at a major university in Hong Kong and the United States in January 2024. As data collection was carried out in two different regions, ethics approval for this study was obtained from the Institutional Review Board of both authors' institutions. Participants provided active informed consent when they enrolled in the study. They were fully informed about the details of their participation in our research study, including the aim of the study, the time required to complete the questionnaire, their right to confidentiality, the compensation for participation, and the ability to withdraw at any time without penalty. Participants completed the questionnaire using the online survey platform Qualtrics. Participants were compensated through course credits or an equivalent.

### Measures

All measures used in the study were originally developed in English. The English measures were then translated into Chinese and subsequently back‐translated into English by bilingual speakers. We utilized multigroup confirmatory factor analyses (MGCFA) to estimate reliabilities using the semTools package (version 0.5–6; Jorgensen et al., 2016) in R 4.2.1 (R Core Team, 2022). Composite reliability coefficients (*ω*) were computed. All models were estimated using the maximum likelihood method. A full list of the items used in the current study can be seen in Tables S1–S3 in the Supplemental Materials.

#### Perceptions of parental guilt and pride induction

Perceived parental guilt and pride induction were measured using the Domain‐Differentiated Guilt Induction scale (DDPGI; Rote et al., 2022) and the Domain‐Differentiated Pride Induction scale (DDPPI), an adaptation of DDPGI.

Participants rated how much their parents made them feel guilty about their behaviors “while they were growing up” using DDPGI. The scale assesses 15 behaviors divided into four domains: moral (4 items), conventional (3 items), prudential (3 items), and personal (5 items). A sample behavior under the moral domain is “lying or cheating”; the conventional domain is “not doing chores or helping out around the house”; the prudential domain is “skipping school, getting poor grades, or not doing your homework”; and the personal domain is “the way you dressed or your appearance.” Participants reported on a 5‐point Likert scale (1 = *not at all true*; 5 = *extremely much*). The scale showed good reliability in both cultures (Hong Kong: *ω* = .93; US: *ω* = .91).

Participants rated how much their parents made them feel proud about their behaviors “while they were growing up” using the DDPPI. Similar to DDPGI, the scale also assesses 15 behaviors divided into four domains: moral (4 items), conventional (3 items), prudential (3 items), and personal (5 items). A sample behavior under the moral domain is “being honest”; the conventional domain is “doing chores or helping out around the house”; the prudential domain is “good school attendance, getting good grades, or doing your homework”; and the personal domain is “the way you dressed or your appearance.” Participants reported on a 5‐point Likert scale (1 = *not at all true*; 5 = *extremely much*). The scale showed good reliability in both cultures (Hong Kong: *ω* = .95; U.S.: *ω* = .94).

In instances where the participants did not engage in the behavior, or it went unnoticed by their parents, they were prompted to assess how much they thought their parents would have made them feel guilt or pride about their behavior.

#### Adolescent–parent relationship quality

Fifteen items from the Networks of Relationships Inventory, Social Provision Version (NRI‐SPV; Furman & Buhrmester, 1985) measured five features of adolescents' relationship quality with their parents, including two negative interaction features (i.e., conflict and antagonism) and three support features (i.e., affection, reassurance, and satisfaction), each feature consisting of three items. Participants reported on a 5‐point Likert scale (1 = *never or hardly at all*; 2 = *seldom or not too much*; 3 = *sometimes or somewhat*; 4 = *often or very much*; 5 = *always or extremely much*). The scale showed good reliability in both cultures (Hong Kong: *ω* = .92 and the U.S.: *ω* = .93). Support features were reliable: affection (HK: *ω* = .92; U.S.: *ω* = .86), reassurance of worth (HK: *ω* = .89; U.S.: *ω* = .88), and satisfaction (HK: *ω* = .94; U.S.: *ω* = .95). Negative interaction features also showed good reliability: conflict (HK: *ω* = .92; U.S.: *ω* = .90) and antagonism (HK: *ω* = .89; U.S.: *ω* = .85).

### Data analysis

All analyses were conducted in R (version 4.2.1; R Core Team, 2022). All codes used in this study are presented in the Supplemental Materials as R‐Markdown documents. To ensure the scales (DDPGI, DDPPI, and NRI‐SPV) were comparable between the Hong Kong and US samples, we examined the measurement invariance of these scales. There are four degrees of measurement invariance: configural invariance (i.e., equivalent factor structure), metric invariance (i.e., equivalent factor loadings), scalar invariance (i.e., equivalent intercepts), and residual invariance (i.e., equivalent error variances). Establishing scalar invariance allows for comparing the mean score of a scale across samples.

We first tested for configural invariance by evaluating the goodness‐of‐fit of a multigroup structural equation model with the same factor structure (four factors for the DDPGI and DDPPI scales and five for the NRI‐SPV) using intercorrelated latent factors without coefficient constraints across the two samples. We applied the criteria of CFI >.90, RMSEA <.08, and SRMR <.08, to indicate a satisfactory model fit (Hu & Bentler, 1999; Kenny et al., 2015; MacCallum et al., 1996). We then tested the other three types of measurement invariance through model comparison: for metric invariance, we compared the metric model (constraining the factor loadings to be equal across samples) against the configural model; for scalar invariance, we compared the scalar model (constraining the item intercepts to be equal across samples) against the metric model; and for residual invariance, we compared the residual model (constraining the error variances to be equal across samples) against the scalar model. Consistent with the widely adopted criteria (Chen, 2007), metric invariance can be rejected if ΔCFI ≥ − .010 supplemented with ΔRMSEA ≥.015 or with ΔSRMR ≥.030, and scalar and residual invariance can be rejected if ΔCFI ≥ − .010 supplemented with ΔRMSEA ≥.015 or with ΔSRMR ≥.010. Since all variables were measured through adolescent self‐reports, we also assessed potential common method bias using Harman's single‐factor test. This approach is recommended for studies with similar designs (Kock et al., 2021). Common method bias is suggested if the first factor in a principal component analysis (PCA) of all study variables accounts for more than 50% of the variance in the data (Fuller et al., 2016).

Following the establishment of measurement invariance, between‐group differences (culture) and within‐group differences (domain/relationship attribute) in perceived parental pride induction, guilt induction, and adolescent–parent relationship quality were assessed via mixed measures ANOVAs. When a significant interaction between culture and domain/relationship attribute was identified, post hoc analyses were performed using pairwise comparisons with Tukey adjustments. Additionally, building on previously observed differences in the perceived legitimacy of guilt induction across nonpersonal and personal domains (Rote & Smetana, 2017), two custom contrast analyses were conducted to examine cultural differences in the disparity between perceived guilt and pride induction in nonpersonal domains (moral, conventional, and prudential) versus the personal domain. These analyses provide a baseline understanding of cultural similarities and differences in the key constructs of interest for this study. To examine hypotheses related to the direction of association between two variables (H1, H4, H5, H6), we calculated bivariate correlations for the entire sample and separately for the Hong Kong and US subsamples, as specified by each hypothesis. Z‐scores are used to compare correlation strengths between culture groups. To examine whether the negative association between guilt induction and adolescent–parent relationship quality is more pronounced in topics perceived as not legitimately regulated by parents (H2) and whether such a negative association is more pronounced among US participants (H3), we conducted two hierarchical linear regression analyses. The outcome of the first regression analysis was perceived support from parents, a composite score generated by averaging items on the affection, reassurance of worth, and satisfaction subscales of the NRI‐SPV. The outcome of the second analysis was negative interactions with parents, a composite score obtained by averaging items on the conflict and antagonism subscales of the NRI‐SPV. We examined the interactions among culture (Hong Kong vs. U.S.), guilt induction domain (personal vs. legitimately regulated), and guilt induction domain scores in each analysis. Consistent with conceptual theorizing (Smetana, 2024) and empirical work with US adolescents (Rote et al., 2022), the “legitimately regulated” domain consisted of all moral, prudential, and conventional issues; intercorrelations among issue types can be found in Figure 3. Simple slope analyses were used to test interaction effects using the “interactions” package (version 1.1.5; Long, 2024). Data visualization was generated using the “ggplot2” (version 3.4.4; Wickham, 2016) and “GGally” (version 2.1.2; Schloerke et al., 2024) in R.

## RESULTS

### Measurement invariance

The goodness‐of‐fit and model comparison results for the scales (DDPGI, DDPPI, and NRI‐SPV) are displayed in Table 1A–C. According to the recommended criteria, the configural models for all three scales showed satisfactory fit. Model comparisons showed satisfactory measurement invariance at the metric, scalar, and residual measurement invariance. Model comparisons (metric vs. configural, scalar vs. metric, residual vs. scalar) showed no significant differences between the compared metric and configural models, as well as scalar and metric models, indicating scalar invariance.

**TABLE 1:** (A) Cross‐cultural (Hong Kong vs. U.S.) measurement invariance of the domain‐differentiated parental guilt induction scale (DDPGI). (B) Cross‐cultural (Hong Kong vs. U.S.) measurement invariance of the domain‐differentiated parental pride induction scale (DDPPI). (C) Measurement invariance (Hong Kong vs. U.S.) of the networks of relationships inventory, social provision version scale (NRI‐SPV).

	Goodness‐of‐fit						Model comparison					
	*χ* ^2^ (*df*)	*p*	RMSEA	90% CI	CFI	SRMR	Ref. model	Δ *χ* ^2^ (*df*)	*p*	∆RMSEA	∆CFI	∆SRMR
(A)												
M1: Configural	240.549 (164)	<.001	0.059	[.042, .075]	0.954	0.067						
M2: Metric	253.084 (175)	<.001	0.058	[.041, .073]	0.953	0.075	M1	12.536 (11)	.325	−0.001	−0.001	0.008
M3: Scalar	277.191 (186)	<.001	0.061	[.045, .075]	0.946	0.080	M2	24.107 (11)	.012	0.003	−0.008	0.005
M4: Residual	313.352 (201)	<.001	0.065	[.051, .078]	0.933	0.085	M3	36.161 (15)	.002	−0.004	−0.013	0.005

	Goodness‐of‐fit						Model comparison					
	*χ* ^2^ (*df*)	*p*	RMSEA	90% CI	CFI	SRMR	Ref. model	∆*χ* ^2^ (*df*)	*p*	∆RMSEA	∆CFI	∆SRMR
(B)												
M1: Configural	243.310 (164)	<.001	0.060	[.044, .076]	0.966	0.042						
M2: Metric	265.510 (175)	<.001	0.062	[.047, .077]	0.962	0.065	M1	22.200 (11)	.023	0.002	−0.005	0.023
M3: Scalar	288.826 (186)	<.001	0.064	[.050, .079]	0.956	0.069	M2	23.317 (11)	.016	0.002	−0.005	0.004
M4: Residual	328.995 (201)	<.001	0.069	[.055, .082]	0.946	0.072	M3	40.169 (15)	<.001	0.005	−0.011	0.003
(C)												
M1: Configural	247.908 (156)	<.001	0.067	[.051, .082]	0.973	0.041						
M2: Metric	270.291 (166)	<.001	0.069	[.053, .083]	0.970	0.055	M1	22.382 (10)	.013	0.002	−0.004	0.014
M3: Scalar	333.885 (176)	<.001	0.082	[.069, .095]	0.954	0.063	M2	63.595 (10)	<.001	0.013	−0.016	0.008
M4: Residual	384.361 (191)	<.001	0.087	[.075, .100]	0.944	0.061	M3	50.475 (15)	<.001	0.005	−0.010	−0.002

*Note*: Residuals for four items across two paths were allowed to correlate to enhance model fit during the measurement invariance process. Detailed specifications of the models, including their correlated paths and outputs, are detailed in the Supplemental Materials.

### Common method bias

The first factor in the principal component analysis (PCA) of all study variables accounted for 28.84% of the variance, which is below the 50% threshold (Fuller et al., 2016). This suggests that common method bias is unlikely to be a concern in our data.

### Descriptive statistics

**FIGURE 1:**
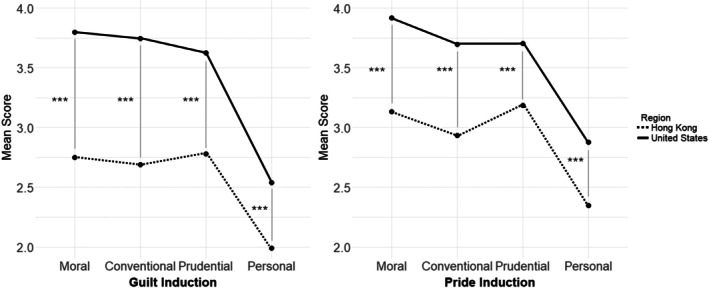
Frequency of self‐conscious emotions induction across different domains. The dashed line represents the induction reported by participants from Hong Kong, while the bold line indicates the induction reported by participants from the United States. Post hoc *t*‐tests revealed that participants in the United States reported significantly higher levels of parental guilt and pride induction across all domains compared to those in Hong Kong, with ***p < .001.

The descriptive statistics for the study variables are presented in Table 2. A mixed measures ANOVA was conducted to investigate perceived parental use of guilt and pride induction, examining the effects of culture (Hong Kong vs. US) and domain (moral, conventional, prudential, and personal) on induction scores. The analysis revealed significant interactions between culture and induction domain for guilt, *F*(3, 792) = 7.929, *p* < .001, partial *η* ^2^ = .029, and for pride, *F*(3, 792) = 5.192, *p* = .002, partial *η* ^2^ = .019 (Figure 1). Post hoc contrasts showed that the differences between Hong Kong and the US in guilt induction scores (*t*(792) = −4.448, *p* < .001) and pride induction scores (*t*(792) = −0.158, *p* = .031) in the personal domain were significantly smaller than the differences in other dimensions.

**TABLE 2:** Descriptive statistics for study variables.

	Hong Kong			United States		
	*M*	*SD*	Min–max	*M*	*SD*	Min–max
1. Guilt induction–Moral	2.75	1.03	1–5	3.80	0.94	1–5
2. Guilt induction–Conventional	2.69	0.83	1–5	3.75	1.01	1–5
3. Guilt induction–Prudential	2.78	1.16	1–5	3.62	1.01	1–5
4. Guilt induction–Personal	1.99	0.83	1–4.8	2.54	1.13	1–5
5. Pride induction–Moral	3.13	0.99	1–5	3.92	1.03	1–5
6. Pride induction–Conventional	2.94	0.89	1–5	3.69	1.04	1–5
7. Pride induction–Prudential	3.19	0.99	1–5	3.70	1.01	1–5
8. Pride induction–Personal	2.35	0.91	1–5	2.88	1.03	1–5
9. A–P relationship: Conflict	2.46	0.89	1–5	2.39	0.98	1–5
10. A–P relationship: Antagonism	2.77	0.87	1–5	2.59	1.03	1–5
11. A–P relationship: Affection	3.74	0.88	1.33–5	4.48	0.87	1–5
12. A–P relationship: Reassurance	3.42	0.89	1–5	3.88	0.99	1–5
13. A–P relationship: Satisfaction	3.47	0.86	1–5	3.79	1.09	1–5

*Note*: A–P relationship = adolescent–parent relationship quality.

**FIGURE 2:**
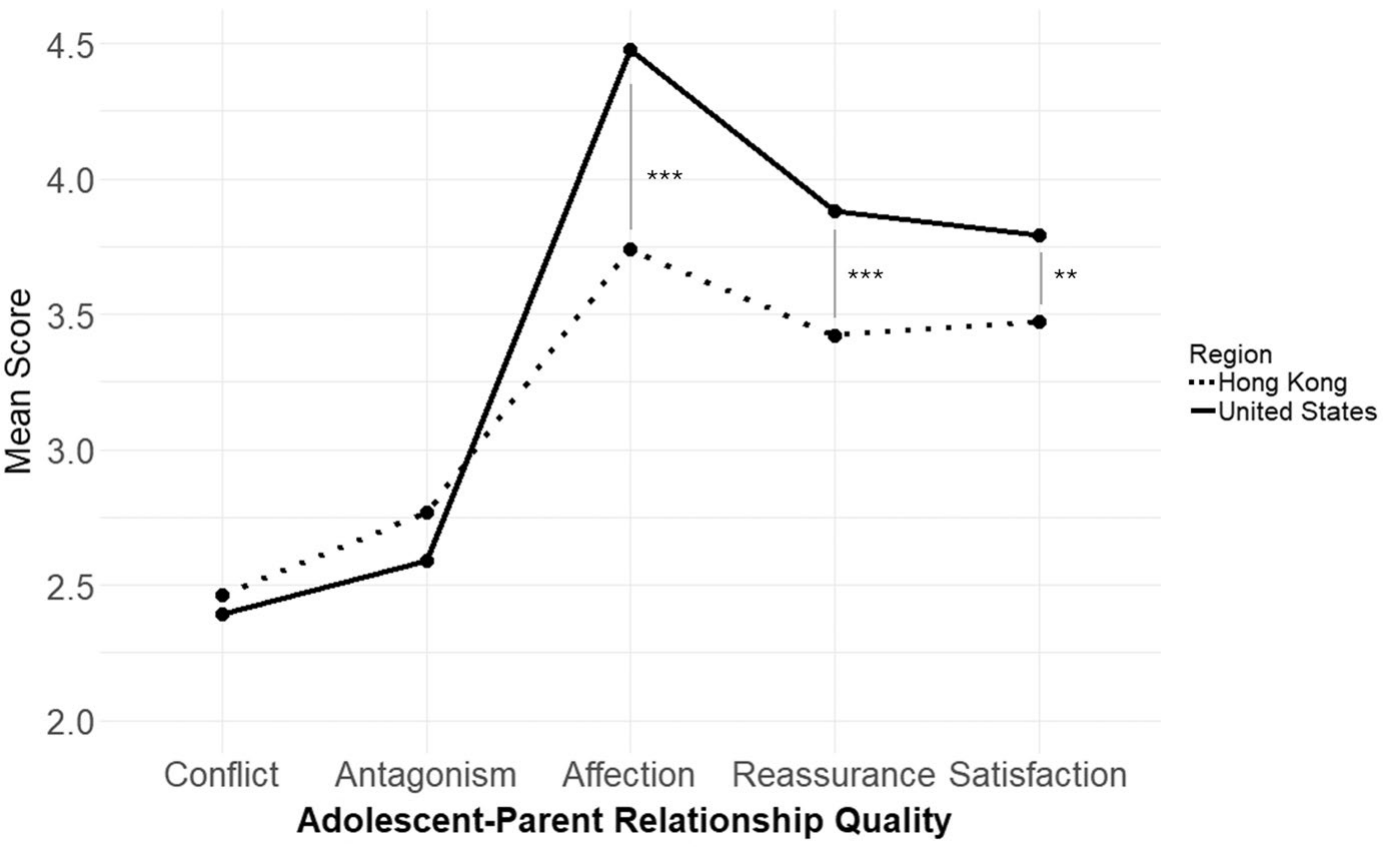
Adolescent–parent relationship quality across different features. The dashed line represents the relationship quality reported by participants from Hong Kong, while the bold line indicates the quality reported by participants from the United States. Post hoc *t*‐tests revealed that participants in the United States reported significantly higher levels of perceived parental support, with ***p < .001 and **p < .01.

Additionally, to examine the differences in adolescent–parent relationship quality between cultures, another mixed measures ANOVA was conducted. This analysis assessed the effects of culture (US vs. Hong Kong) and relationship features, including two negative interaction features (conflict and antagonism) and three support features (affection, reassurance, and satisfaction), on adolescent–parent relationship quality scores. The results revealed significant interactions between culture and relationship quality features, *F*(4, 792) = 11.700, *p* < .001, partial *η* ^2^ = .043 (see Figure 2). Post hoc t‐tests showed that adolescents in the U.S. reported higher levels of parental support (affection, reassurance, and satisfaction). However, there were no significant differences between the two cultures regarding adolescents' reports of negative interactions with their parents. Detailed results of the post hoc *t*‐tests can be found in the Supplemental Materials.

### Domain‐differentiated guilt induction and adolescent–parent relationship

**FIGURE 3:**
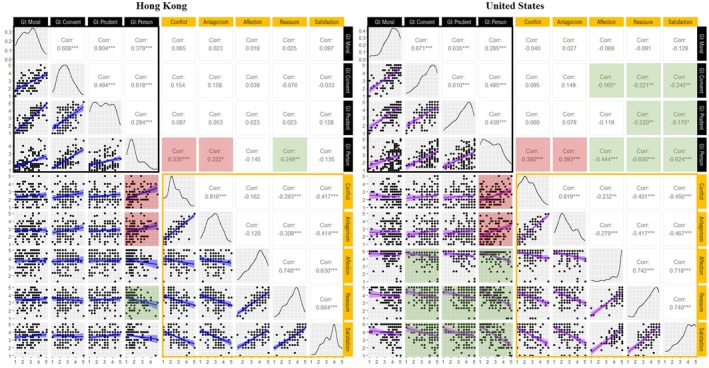
Correlations and density plots for domain‐differentiated guilt induction and adolescent–parent relationship quality. Blue regression lines indicate correlations for Hong Kong, while purple regression lines represent correlations for the U.S. GI = Guilt Induction; Convent = Conventional; Prudent = Prudential. Black square outlines (□) denote correlations within Guilt Induction domains, and yellow square outlines (□) highlight correlations across relationship quality features. Positive correlations between guilt induction and relationship quality are represented by red shading, whereas negative correlations are indicated by green shading. *p < .05, **p < .01, and ***p < .001 sample, perceived guilt induction in the conventional, prudential, and personal domains was correlated with lower perceived support from parents (*rs* = −.165 to −.624). Additionally, perceived guilt induction in the personal domain was positively correlated with negative interactions (conflict: *r* = .380; antagonism: *r* = .393). Only perceived guilt induction in the moral domain appeared unrelated to adolescent–parent relationship quality in the U.S.

The density plots and correlations between guilt induction domains and various aspects of the adolescent–parent relationship are presented in Figure 3. In both cultures, positive correlations were observed among different domains of guilt induction, suggesting that the perceived level of parental guilt induction in one domain is positively associated with its perceived use in other domains. Partially supporting our hypothesis (H1), perceived levels of parental guilt induction in certain domains were negatively associated with adolescent–parent relationship quality. Specifically, in the Hong Kong sample, perceived guilt induction in the personal domain showed a negative correlation with adolescents' perceived reassurance from their parents and a positive correlation with negative interactions (conflict: *r* = .335; antagonism: *r* = .222). Perceived guilt induction in other domains did not demonstrate a significant association with adolescent–parent relationship quality in Hong Kong. In the U.S.

Results of our regression analyses indicated that perceived guilt induction in the personal domain was associated with poorer adolescent–parent relationships compared to perceived guilt induction in other domains, which aligned with our hypothesis (H2). Specifically, the interaction between guilt induction domain (i.e., personal domain vs. legitimately regulated domains) and mean scores for guilt induction was significant for regressions on perceived positive support from parents (*β* = −0.21, *SE* = 0.10, *t* = −2.201, *p* = .028) and negative interaction with parents (*β* = 0.22, *SE* = 0.11, *t* = 2.08, *p* = .038). Simple slope analysis indicated that perceived guilt induction in the personal domain was more negatively correlated with perceived positive support from parents (*β* = −0.19, *SE* = 0.09, *t* = −2.13, *p* = .030) compared to perceived guilt induction in legitimately regulated domains (*β* = 0.03, *SE* = 0.04, *t* = 0.65, *p* = .520). Additionally, perceived guilt induction in the personal domain showed a stronger positive correlation with negative interactions with parents (*β* = 0.30, *SE* = 0.10, *t* = 3.07, *p* < .001) than that observed in legitimately regulated domains (*β* = 0.07, SE = 0.05, *t* = 1.64, *p* = .100). Supporting hypotheses (H3), we also found a significant interaction between mean scores of perceived guilt induction and culture (i.e., Hong Kong vs. U.S.) in our regression analysis on perceived positive support from parents (*β* = −0.19, *SE* = 0.06, *t* = −3.28, *p* = .001). A simple slope analysis was performed to further understand this interaction. For U.S. participants, the relationship between perceived guilt induction and perceived support from parents was significantly negative (*β* = −0.24, *SE* = 0.03, *t* = −7.29, *p* < .001), whereas for Hong Kong participants, the relationship was not significant (*β* = −0.03, *SE* = 0.04, *t* = 0.70, *p* = .490). Results of z‐score comparisons indicate that the negative associations between perceived guilt induction in the personal domain and aspects of perceived parental support were more pronounced in the U.S. sample (affection: *z* = −2.664, *p* = .008; reassurance: *z* = −3.529, *p* < .001; satisfaction: *z* = −4.791, *p* < .001). The negative association between perceived guilt induction in the prudential domain and adolescents' perceived relationship satisfaction with their parents was also stronger in the U.S. sample (*z* = −1.983, *p* = .047). The strength of correlations between perceived guilt induction in the conventional domain and aspects of perceived parental support did not significantly differ across cultures, although they were only significantly negative in the United States. The correlations between perceived guilt induction in the moral domain and adolescent–parent relationship quality aspects were not significant in either cultural context.

### Domain‐differentiated pride induction and adolescent–parent relationship

**FIGURE 4:**
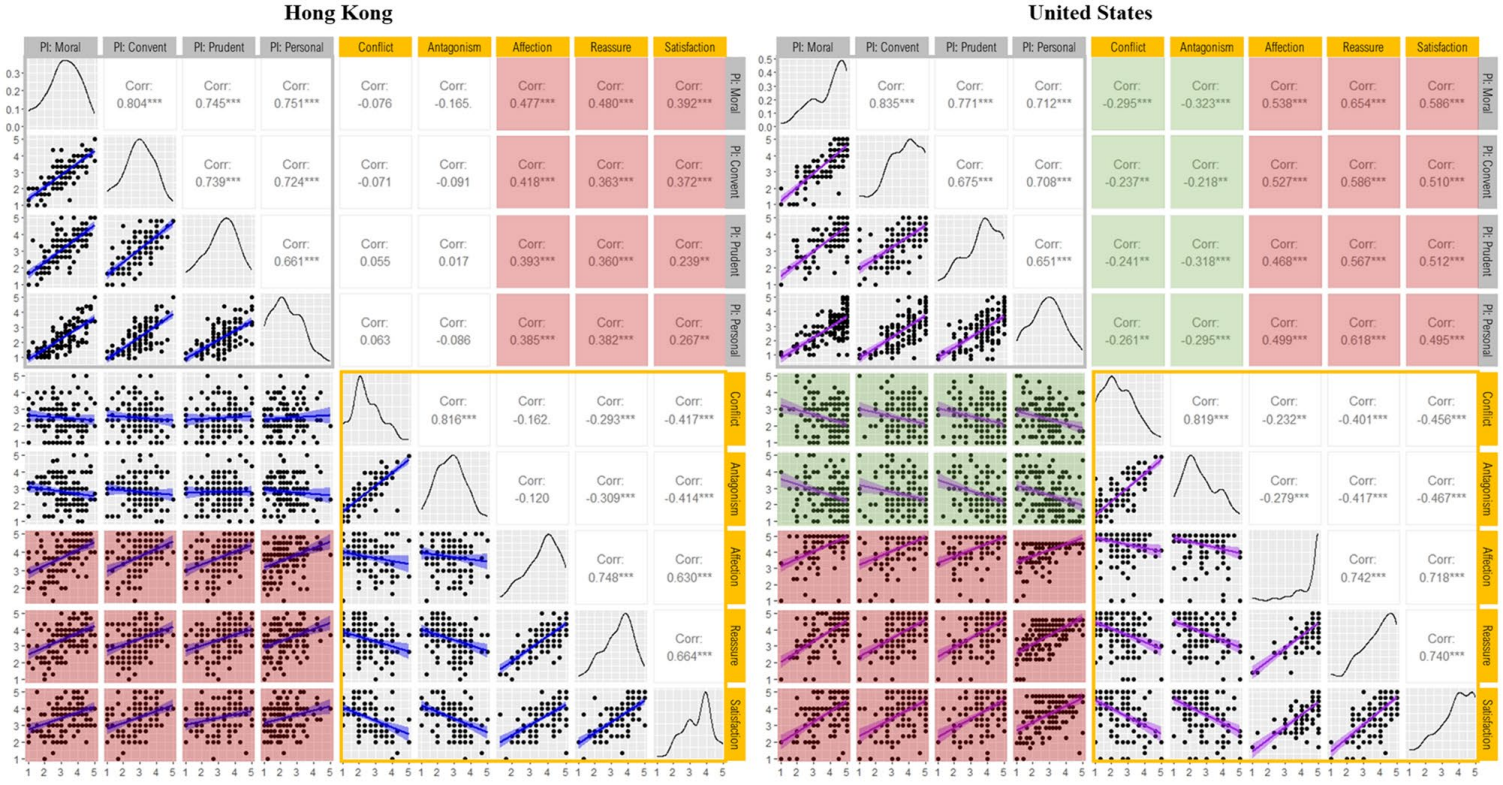
Correlations and density plots for domain‐differentiated guilt induction and adolescent–parent relationship quality. Blue regression lines indicate correlations for Hong Kong, while purple regression lines represent correlations for the U.S. PI = Pride Induction; Convent = Conventional; Prudent = Prudential. The gray square outlines (□) denote correlations within Pride Induction domains, and the yellow square outlines (□) highlight correlations across relationship quality features. Positive correlations between guilt induction and relationship quality are represented by red shading, whereas negative correlations are indicated by green shading. **p < .01, and ***p < .001.

Figure 4 displays the density plots and correlations between pride induction domains and various aspects of the adolescent–parent relationship. In both cultures, we observed positive correlations among different pride domains, indicating that perceived parental pride induction in one domain was associated with its perceived use in others. Supporting our hypothesis (H4), perceived parental pride induction positively correlated with the quality of the adolescent–parent relationship across both cultures. Specifically, perceived parental pride induction was positively correlated with adolescents' perceived support from their parents—encompassing affection, reassurance, and relationship satisfaction (*rs* = .239 to .654) in both samples from Hong Kong and the U.S. Additionally, in the U.S. sample, perceived parental pride induction was associated with fewer parent–child negative interactions, evidenced by its negative correlation with conflict and antagonism (*rs* = −.218 to −.323).

### Domain‐differentiated guilt induction and pride induction

**FIGURE 5:**
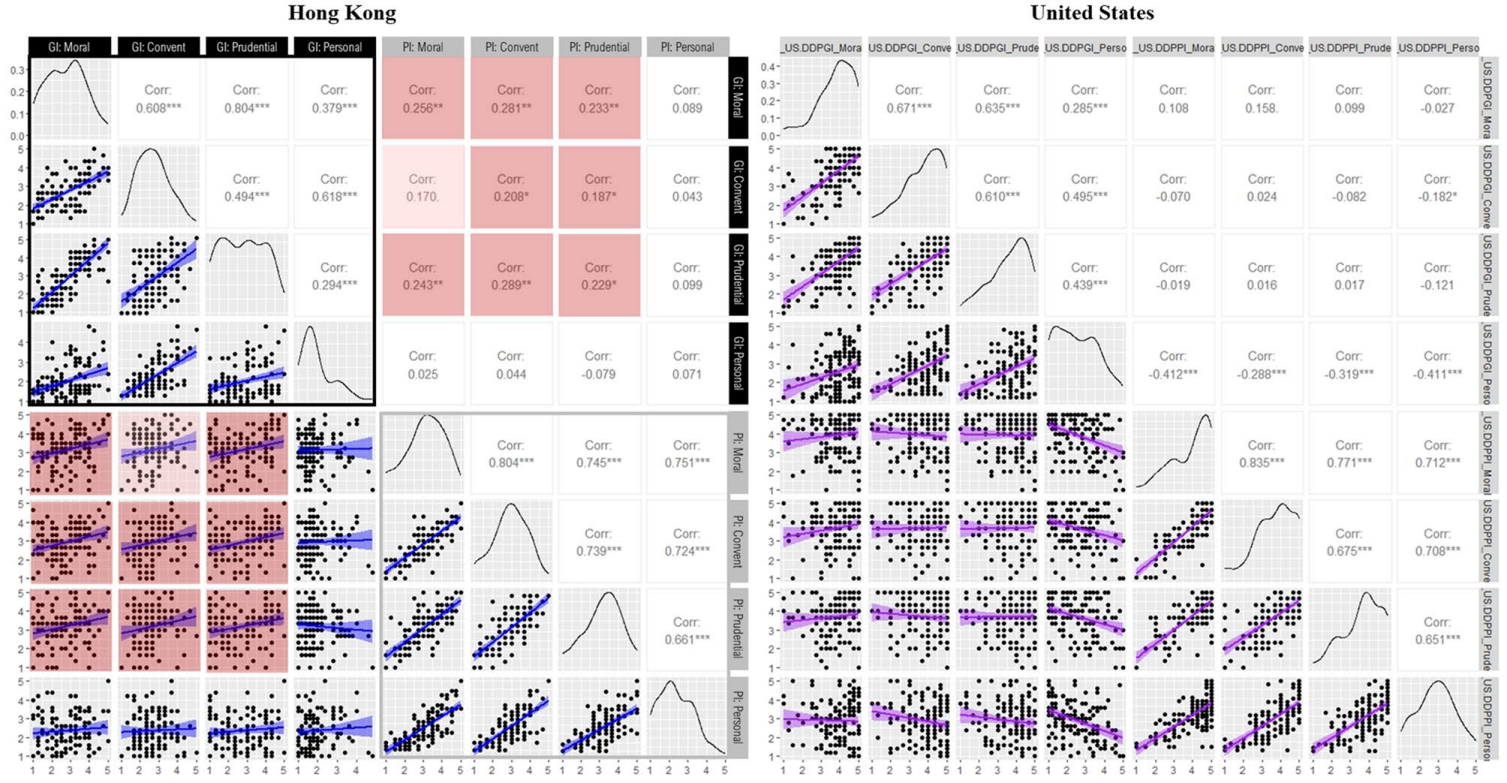
Correlations and density plots for domain‐differentiated guilt induction and domain‐differentiated pride induction. Blue regression lines indicate correlations for Hong Kong, while purple regression lines represent correlations for the U.S. GI = Guilt Induction; PI = Pride Induction; Convent = Conventional. The black square outlines (□) denote correlations within Guilt Induction domains, and the gray square outlines (□) highlight correlations within Pride Induction domains. Positive correlations between guilt and pride induction are represented by red shading, whereas negative correlations are indicated by green shading. *p < .05, **p < .01, ***p < .001, and †*p* = .05867.

Figure 5 presents the correlations among different domains of perceived guilt induction and pride induction, as well as the correlations between perceived induction of these two self‐conscious emotions across domains. Distinct cultural differences emerged in the correlations between perceived guilt and pride induction across domains, as expected. Consistent with our hypothesis (H5), the Hong Kong‐Chinese sample demonstrated positive correlations between perceived pride induction and guilt induction in moral, conventional, and prudential domains (*r*s ranging from .187 to .289). Most of these correlations were statistically significant, except for the correlation between perceived pride induction in the moral domain and perceived guilt induction in the conventional domain, which approached significance (*p* = .059). In contrast, in support of H6, the U.S. sample demonstrated nil or negative correlations between perceived parental pride induction and guilt induction, with perceived guilt induction in the personal domain particularly showing consistent negative correlations with perceptions of pride induction (*r*s ranging from −.288 to −.412).

## DISCUSSION

This study addresses critical gaps in the literature on parental socialization of self‐conscious emotions and their association with adolescent–parent relationships. We investigated adolescents' domain‐differentiated perceptions of parental pride and guilt induction, as conceptualized by the social cognitive domain theory (Smetana, 2024), and their associations with adolescents' perceived relationship quality with their parents. By comparing adolescents from the United States and Hong Kong, we offer insights into cultural differences in parent emotion socialization practices and their relational consequences. Our findings revealed both cultural similarities and differences, with perceptions of pride induction generally linked to positive relationship quality and perceptions of guilt induction showing more context‐specific associations. This study's unique contribution lies in its domain‐differentiated approach and cross‐cultural perspective, offering a comprehensive examination of parental emotion socialization strategies and their associations with adolescent–parent relationships in diverse settings.

### Measurement invariance for domain‐differentiated guilt and pride induction

Our study established scalar invariance for a four‐factor structure—comprising moral, conventional, prudential, and personal domains—within the domain‐differentiated guilt and pride induction scales across two groups of adolescents from Hong Kong and the United States. The achievement of scalar invariance underscores the universality of understanding and differentiating various types of social knowledge, as conceptualized by the social‐cognitive domain theory (Smetana, 2024). The domain‐differentiated guilt induction scale was initially developed and tested by Rote et al. (2022), who found that U.S. adolescents can distinguish between issues legitimately regulated by parents and those concerning personal choice (Rote et al., 2022). Our research builds on this foundation by examining the domain‐differentiated approach in parental guilt and pride induction across Eastern and Western cultures, thereby expanding our understanding of parental self‐conscious emotional socialization practices. The scalar invariance we achieved offers both theoretical and methodological significance. Theoretically, it demonstrates the universality of differentiation across the four domains in guilt and pride induction. Methodologically, scalar invariance allows for valid group comparisons across cultures—an aspect not established in previous studies of social domains.

### Parental guilt induction and adolescent–parent relationship across cultures

With scalar invariance established for our scales, we investigated the links between parental self‐conscious emotion induction and the quality of adolescent–parent relationships. Our findings indicated potential relational costs associated with perceptions of parents' use of guilt induction, aligning with previous research on parental psychological control (Choe et al., 2023) and parental conditional regard (Haines & Schutte, 2023). However, contrary to the prevailing narrative in these fields, which often characterizes emotional induction as emotional manipulation and inherently detrimental, our results revealed that the negative associations between perceived guilt induction and adolescent–parent relationship quality were not uniform across all domains. This finding is consistent with other work on domain‐differentiated guilt induction (Rote et al., 2022; Rote & Smetana, 2017; Smetana et al., 2021) and underscores the necessity of differentiating between various domains of social knowledge when examining their associations with youth adjustment (Smetana, 2024).

In our study, we found that adolescents' perceptions of parental guilt induction within the personal domain correlated with poorer adolescent–parent relationship quality, characterized by more negative interactions and less perceived parental support, across both cultural contexts. In contrast, perceived guilt induction in the moral domain did not show any correlation with adolescent–parent relationships in either culture. This suggests that adolescents may view parental guilt induction related to personal matters as more detrimental and intrusive than that related to moral issues. This perspective aligns with the findings of Rote and Smetana (2017), which indicated that U.S. youth view parental guilt induction for moral transgressions as more acceptable, while they evaluate guilt induction regarding personal issues less favorably.

Although the association between perceived guilt induction in the personal domain and adolescent–parent relationship quality was consistently negative across both cultures, we identified significant cultural differences that warrant further exploration. Specifically, our data suggest that perceived guilt induction may have a stronger negative association with adolescent–parent relationship quality in the United States compared to Hong Kong. In addition to the negative associations observed in the personal domain, we found significant negative correlations between parental guilt induction in the prudential and conventional domains and adolescent–parent relationship quality within the U.S. sample. This indicates that U.S. adolescents may be experiencing a heightened desire for autonomy during adolescence (Nucci, 2001). In contrast, this pattern was not evident in the Hong Kong sample, likely due to the cultural context in which guilt induction is generally perceived as an acceptable means of promoting adherence to parental expectations and collectivistic values (Fung & Lau, 2012; Rudy & Halgunseth, 2005). As a result, adolescents in Hong Kong may be more receptive to guilt induction related to conventional and prudential issues than their U.S. counterparts.

Additionally, we observed that the negative association between perceived levels of parental guilt induction in the personal domain and adolescents' perceived parental support—including affection, reassurance of worth, and relationship satisfaction—was significantly stronger in the U.S. sample than in the Hong Kong sample. This disparity may be linked to the strong emphasis on autonomy and independence within families in individualistic cultures (Nucci, 2001; Wang et al., 2008), suggesting that guilt induction in personal domains can be perceived as particularly intrusive and detrimental to adolescent–parent relationships in these contexts. In contrast, in collectivistic cultures like Hong Kong, where familial interconnectedness and adherence to social norms are prioritized (Fung & Lau, 2012), adolescents may perceive parental guilt induction less negatively, potentially mitigating some of its adverse effects on perceived parental support, even within the personal domain.

### Parental pride induction and adolescent–parent relationship across cultures

As expected, adolescents' perceptions of parental pride induction were associated with better adolescent–parent relationship quality in both cultures. Specifically, perceived pride induction across all domains—moral, conventional, prudential, and personal—positively correlated with all aspects of perceived parental support (i.e., affection, reassurance of worth, and relationship satisfaction) in both Hong Kong and U.S. samples. This aligns with studies indicating that appropriate parental praise has beneficial effects on youth (Lee et al., 2016).

Consistent with our argument, pride induction contingent on adolescents' behaviors (e.g., keeping promises, doing chores, maintaining good academic performance, and engaging in leisure activities) did not appear to negatively impact adolescent–parent relationship quality. This contrasts sharply with the negative association observed between conditional positive regard and relatedness (Haines & Schutte, 2023). We propose that pride induction maintains its positive association with adolescent–parent relationship quality, even when conditional, because unlike parental acceptance, which is expected to be unconditional (Ryan & Deci, 2008), pride is typically understood to be bestowed conditionally based on specific achievements or behaviors (Li, Song, Zhou, et al., 2024).

We also observed cultural differences in the association between parental pride induction and adolescent–parent relationship quality. In the U.S. sample, perceived pride induction was associated with fewer negative interactions between adolescents and parents, a pattern not evident in the Hong Kong sample. This divergence may stem from differing cultural perspectives on pride induction. In individualistic cultures, parents often use pride induction to promote independence (Wang et al., 2008), potentially aligning with adolescents' increasing desire for autonomy and leading to fewer conflicts. Conversely, in collectivistic cultures like Hong Kong, pride induction is viewed as one of the means to encourage adherence to parental expectations (Wang et al., 2008). While this approach enhances perceived parental support, it may not necessarily reduce conflicts, as it does not directly address adolescents' growing need for independence. Thus, in individualistic cultures, increased use of pride induction may correlate with fewer negative interactions during a period when adolescents crave more independence from their parents.

### Parental pride induction and parental guilt induction across cultures

Consistent with our hypotheses, the association between perceived parental pride induction and parental guilt induction differed between the two cultures. In the Hong Kong sample, perceived parental pride induction and guilt induction were positively correlated in domains generally acknowledged as legitimately regulated by parents (i.e., moral, conventional, and prudential; Rote et al., 2022). These associations were not significant in the U.S. sample. Conversely, in the U.S. sample, perceived parental pride induction and guilt induction in personal domains were negatively correlated, while this association was not significant in the Hong Kong sample. These findings align with research on praise and criticism, which indicates a positive association in Chinese samples (e.g., Li, Song, & Zhou, 2024) but a negative association in Western samples (e.g., Gunderson et al., 2018).

Our results suggest that Chinese parents might use both guilt (Fung & Lau, 2012; Rudy & Halgunseth, 2005) and pride induction (Wang et al., 2008) to foster child regulation and promote adherence to expectations, particularly in areas related to personal and others' welfare and societal values. In contrast, individualistic cultures often prioritize nurturing children's self‐esteem (Miller et al., 2002), leading to guilt induction being perceived as more problematic and associated with negative perceptions of the child (Rudy & Halgunseth, 2005). Simultaneously, in such cultures, pride induction is viewed more positively and used to promote independence (Wang et al., 2008). The negative association between perceived pride induction and guilt induction in the personal domain in the U.S. sample suggests that guilt induction in this domain might be particularly associated with negative perceptions of the child, correlating with less pride induction in those areas. While further research is needed to test these claims, our study provides a strong foundation for such comparisons.

As an exploratory aspect of our study, we compared the frequency of perceived parental pride and guilt induction between cultures. Interestingly, adolescents from the U.S. reported higher levels of both parental guilt induction and pride induction across all domains compared to their Hong Kong counterparts. This difference is less likely to be due to a general rating bias, as the two samples did not differ in their ratings for adolescent–parent negative interactions. The higher levels of parental pride induction in the U.S. sample are consistent with literature suggesting that positive emotions are valued more in Western cultures (Campos & Kim, 2017; Tsai et al., 2007). However, the higher parental guilt induction in the U.S. sample is unexpected, given that East Asian cultures typically value negative emotions more (Campos & Kim, 2017; Tsai et al., 2007) and report more parental guilting and shaming (Chao & Aque, 2009; Rudy & Halgunseth, 2005).

This unexpected finding might be related to our sample characteristics, particularly the recruitment of participants from a top university in Hong Kong. As these students were likely high‐achieving, their parents might have used less guilt induction across all domains during their upbringing. Alternatively, this could reflect a true emotional expressivity difference between U.S. and Hong Kong parents, as open emotional displays are often deemed normative in Western cultures (Butler et al., 2007), while a balanced emotional state is preferred in East Asian countries (Miyamoto & Ryff, 2011). This cultural difference might lead to generally lower levels of both pride and guilt induction among Hong Kong parents. Finally, this finding may be due to a distinction between *perceived* parental guilt induction and actual guilt induction behaviors. Youth are less likely to perceive that parents *made them* feel guilty about an issue if they already felt guilty about the issue before interacting with their parent (Rote et al., 2017). Given the greater emphasis on guilt in collectivist cultures (Furukawa et al., 2012), it may be that Hong Kong adolescents perceived less parental guilt induction than U.S. youth, independent of parental behavior, because they interpreted their guilt as stemming from more internal sources. The same attribution dynamic may extend to perceptions of pride. If U.S. adolescents experience more pride in general, they might attribute some of this emotion to self‐generated achievement rather than parental induction. Nonetheless, U.S. adolescents in our sample still reported higher perceived parental pride induction than Hong Kong adolescents, perhaps reflecting that American parents tend to express pride more explicitly. Future research should further investigate how cultural norms around emotion expression and self‐conscious emotion attribution jointly shape adolescents' perceptions of parental guilt and pride induction.

While we can only speculate on these explanations, our study provides a strong foundation for further research in the area of parental socialization of self‐conscious emotions. Future studies should aim to disentangle these potential explanations and explore the implications of these cultural differences in emotion socialization practices.

### Limitations and strengths

Our study has several limitations to consider when interpreting the results. First, the cross‐sectional nature of our data precludes drawing causal conclusions about the relationship between parental pride/guilt induction and adolescent–parent relationship quality. Our approach of asking participants to reflect on parental guilt and pride induction during their upbringing provides valuable insight into the potential long‐term influence of these practices. However, retrospective assessments have limitations due to their reliance on memory, which could be influenced by current perspectives. While our findings are informative and consistent with previous literature, future research employing experimental designs or longitudinal observations is necessary to determine the directionality of these relationships.

Second, our use of convenience sampling may have introduced some bias in comparisons between the U.S. and Hong Kong samples. Although we attempted to mitigate this by recruiting primarily from universities using similar methods and age requirements, and our demographics indicate similar socioeconomic backgrounds, future research should employ more rigorous sampling methods to ensure greater comparability between samples.

Third, our reliance on single‐informant self‐report data may limit the generalizability of our findings. Although adolescent self‐reports are particularly valuable for capturing adolescents' perceptions, which are central to understanding the influence of parental emotion socialization strategies on adolescent–parent relationships, such assessments may not fully capture all dimensions of the relationship between these constructs. Previous research has shown discrepancies between self‐reported and observed emotion expressivity, and differences in associations with child outcomes (Chen et al., 2015). Future studies should employ multi‐informant, multi‐method approaches to confirm and extend our findings.

Despite these limitations, our study has notable strengths. It is among the first to consider the socialization of both positive and negative self‐conscious emotions and their link to adolescent–parent relationship quality. Our findings have important implications for future research on the relational consequences of parental guilt and pride induction.

The domain‐differentiated approach (Rote et al., 2022; Smetana, 2024) used in this study highlights the importance of recognizing nuances in the effects of self‐conscious emotion induction on adolescent–parent relationships. Methodologically, we have established scalar invariance for the domain‐differentiated guilt induction and the domain‐differentiated pride induction scale, providing a robust framework for future cross‐cultural comparisons of guilt and pride induction across social domains. Doing so also furthers evidence that youth across cultures conceptualize similar issues as primarily personal choice and differentiate these behaviors from others in terms of legitimate parental authority (Smetana, 2024).

Our study also has practical implications. The findings reveal that guilt induction in the personal domain could be particularly detrimental to adolescent–parent relationships, leading to more negative interactions and less adolescent‐perceived support from parents. This pattern is consistent across cultures, underscoring the universality of the need for autonomy in parent–child relationships. During adolescence, when there is an increasing desire for autonomy and a developmental goal to establish independence, parents should be particularly mindful about not inducing guilt for their child's personal choices.

### Conclusion

In conclusion, this study examined the association between parental pride and guilt induction across different domains and adolescent–parent relationship quality in the United States and Hong Kong. Our domain‐differentiated approach provides a nuanced understanding of parental self‐conscious emotion socialization and its relational implications across cultures. The findings suggest that while guilt induction in the personal domain may be particularly detrimental to adolescent–parent relationships, pride induction is generally positively associated with relationship quality in both cultural contexts. However, the extent and nature of these associations vary between cultures, likely reflecting differences in cultural norms and parenting beliefs. This research contributes significantly to our understanding of parental emotion socialization across cultures, offering important theoretical insights, methodological advancements, and practical implications for fostering positive parent–adolescent relationships in diverse cultural settings. Future research can build upon these findings to further explore the complexities of emotion socialization and its impact on family dynamics across different cultural contexts.

## AUTHOR CONTRIBUTIONS

**Wendy M. Rote:** Conceptualization; investigation; methodology; writing – review and editing; validation. **Natalie Wong:** Conceptualization; investigation; writing – original draft; methodology; formal analysis; writing – review and editing; validation; visualization.

## FUNDING INFORMATION

The work described in this article was partially supported by a fellowship award from the Research Grants Council of the Hong Kong Special Administrative Region, China (Project No. CUHK PDFS2122‐4H05).

## CONFLICT OF INTEREST STATEMENT

The authors declare no conflict of interest.

## ETHICS STATEMENT

This study was approved by the Survey and Behavioral Research Ethics Committee (SBRE) of the Chinese University of Hong Kong (Protocol #: SBRE‐23‐0086; approved on October 6, 2023) and the Institutional Review Board (IRB) of the University of South Florida (Study # 006418; approved on November 1, 2023).

## CONSENT STATEMENT

All participants have provided their informed consent before taking part in the study. No participants were under the age to consent to participation.

## Supporting information


Table S1.

Data S1:

Data S2:

Data S3:

Data S4:

Data S5:

## Data Availability

The data that support the findings of this study are available from the corresponding author upon reasonable request.
