# Supplementary material for: How parents leverage guilt and pride: A comparison of parental guilt and pride induction in Hong Kong and the United States
Source: J Res Adolesc. 2025 Dec 10;35(4):e70107. doi: 10.1111/jora.70107 (PMC12696220; doi:10.1111/jora.70107)
Supplement: Supplementary file 1 — Table S1. [file JORA-35-0-s006.docx]

**Supplemental Materials**

This document provides detailed instructions, along with the reliability of each factor and the factor loadings for each item of the following scales:

(1) Domain-Differentiated Guilt Induction Scale (DDPGI)

(2) Domain-Differentiated Pride Induction Scale (DDPPI)

(3) Networks of Relationships Inventory, Social Provision Version (NRI-SPV)

We conducted Multiple Group Confirmatory Factor Analysis (MGCFA) to establish scalar invariance across participants from Hong Kong and the United States. To ensure consistency and interpretability, we present the factor loadings derived from the scalar invariant models, excluding those from the configural and metric models. The complete code and output for the MGCFA, including results from the configural, metric, and residual models, can be found in the supplementary R-Markdown document.

**Domain-Differentiated Guilt Induction scale (DDPGI)**

Detailed instructions, reliability of each factor, and factor loading of each item of DDPGI are listed below. For consistency, only the factor loadings and reliabilities of the scalar invariant model are presented below. The complete code and output for the MGCFA are available in the supplemental R-Markdown.

The following items ask about the extent to which your parents made you feel guilty about various behaviors while you were growing up. Please rate each statement from 1 (not at all) to 5 (extremely much). If you did not engage in the behavior or your parents never found out, please rate how much you think your parents would have made you feel guilty about it. If the answer to a question varies depending on the parent considered, please select the answer with the higher score (e.g., select 4 “often or very much” rather than 3 “sometimes or somewhat”).

**Table S1.** Factor loadings from confirmatory factor analysis of the Domain-Differentiated Guilt Induction scale (DDPGI)

|  |  | **HK** | | **US** | |
| --- | --- | --- | --- | --- | --- |
| While you were growing up, how much did your parents make you feel guilty about… | **No.** | **𝜔** | **Factor loading** | **𝜔** | **Factor loading** |
| **Factor 1: Moral Domain** |  | 0.833 |  | 0.797 |  |
| Saying mean things about others or hurting other people | DDPGI_Moral_1 |  | 0.692 |  | 0.664 |
| Breaking promises or not following through with things you said you’d do | DDPGI_Moral_2 |  | 0.639 |  | 0.575 |
| Lying or cheating | DDPGI_Moral_3 |  | 0.828 |  | 0.856 |
| Stealing or taking things from others without asking | DDPGI_Moral_4 |  | 0.703 |  | 0.732 |
| **Factor 2: Conventional Domain** |  | 0.651 |  | 0.710 |  |
| Talking back, using foul language, or not using proper manners | DDPGI_Conven_5 |  | 0.629 |  | 0.692 |
| Breaking rules, violating curfew, or otherwise not doing what’s expected of you | DDPGI_Conven_6 |  | 0.649 |  | 0.732 |
| Not doing chores or helping out around the house | DDPGI_Conven_7 |  | 0.530 |  | 0.607 |
| **Factor 3: Prudential Domain** |  | 0.658 |  | 0.688 |  |
| Skipping school, getting poor grades, or not doing your homework | DDPGI_Pruden_8 |  | 0.772 |  | 0.758 |
| Doing things that could be risky or bad for your health | DDPGI_Pruden_9 |  | 0.638 |  | 0.591 |
| Drinking, smoking, or doing drugs | DDPGI_Pruden_10 |  | 0.567 |  | 0.615 |
| **Factor 4: Personal Domain** |  | 0.830 |  | 0.850 |  |
| The way you dressed or your appearance | DDPGI_Person_11 |  | 0.720 |  | 0.737 |
| How you spent your own money (e.g., allowance, gifts, paychecks) | DDPGI_Person_12 |  | 0.679 |  | 0.730 |
| The music you listened to or the TV shows or movies you watched | DDPGI_Person_13 |  | 0.648 |  | 0.664 |
| Who you hung out with or had as friends | DDPGI_Person_14 |  | 0.728 |  | 0.780 |
| What you did in your free time (e.g., play video-games, go shopping, play sports, etc.) | DDPGI_Person_15 |  | 0.684 |  | 0.746 |
| **Scale Total** |  | 0.932 |  | 0.905 |  |

*Note.* 𝜔 = Composite Reliability (𝜔); Modification indices suggested the presence of correlated residuals, and thus, residuals for four items across
two paths were allowed to correlate: DDPGI_Moral_4 ~~ DDPGI_Pruden_10; DDPGI_Conven_7 ~~ DDPGI_Person_15.

**Domain-Differentiated Pride Induction scale (DDPPI)**

Detailed instructions, reliability of each factor, and factor loading of each item of DDPPI are listed below. For consistency, only the factor loadings and reliabilities of the scalar invariant model are presented below. The complete code and output for the MGCFA are available in the supplemental R-Markdown.

The following items ask about the extent to which your parents made you feel proud about various behaviors while you were growing up. Please rate each statement from 1 (not at all) to 5 (extremely much). If you did not engage in the behavior or your parents never found out, please rate how much you think your parents would have made you feel proud about it. If the answer to a question varies depending on the parent considered, please select the answer with the higher score (e.g., select 4 “often or very much” rather than 3 “sometimes or somewhat”).

**Table S2.** Factor loadings from confirmatory factor analysis of the Domain-Differentiated Pride Induction scale (DDPPI)

|  |  | **HK** | | **US** | |
| --- | --- | --- | --- | --- | --- |
| While you were growing up, how much did your parents make you feel proud about… | **No.** | **𝜔** | **Factor loading** | **𝜔** | **Factor loading** |
| **Factor 1: Moral Domain** |  | 0.866 |  | 0.921 |  |
| Saying nice things about others or helping other people | DDPPI_Moral_1 |  | 0.759 |  | 0.830 |
| Keeping promises or following through with things you said you’d do | DDPPI_Moral_2 |  | 0.865 |  | 0.855 |
| Being honest | DDPPI_Moral_3 |  | 0.818 |  | 0.891 |
| Working hard for the things you wanted | DDPPI_Moral_4 |  | 0.678 |  | 0.746 |
| **Factor 2: Conventional Domain** |  | 0.703 |  | 0.779 |  |
| Being polite, using nice language, or using proper manners | DDPPI_Conven_5 |  | 0.645 |  | 0.766 |
| Following rules, obeying curfew, or otherwise doing what’s expected of you | DDPPI_Conven_6 |  | 0.733 |  | 0.850 |
| Doing chores or helping out around the house | DDPPI_Conven_7 |  | 0.594 |  | 0.671 |
| **Factor 3: Prudential Domain** |  | 0.728 |  | 0.736 |  |
| Good school attendance, getting good grades, or doing your homework | DDPPI_Pruden_8 |  | 0.661 |  | 0.758 |
| Avoiding things that could be risky or bad for your health | DDPPI_Pruden_9 |  | 0.764 |  | 0.705 |
| Not Drinking, smoking, or doing drugs | DDPPI_Pruden_10 |  | 0.627 |  | 0.665 |
| **Factor 4: Personal Domain** |  | 0.861 |  | 0.853 |  |
| The way you dressed or your appearance | DDPPI_Person_11 |  | 0.796 |  | 0.782 |
| How you spent your own money (e.g., allowance, gifts, paychecks) | DDPPI_Person_12 |  | 0.696 |  | 0.729 |
| The music you listened to or the TV shows or movies you watched | DDPPI_Person_13 |  | 0.695 |  | 0.702 |
| Who you hung out with or had as friends | DDPPI_Person_14 |  | 0.707 |  | 0.736 |
| What you did in your free time (e.g., play video-games, go shopping, play sports, etc.) | DDPPI_Person_15 |  | 0.778 |  | 0.706 |
| **Scale Total** |  | 0.949 |  | 0.944 |  |

*Note.* 𝜔 = Composite Reliability (𝜔); Modification indices suggested the presence of correlated residuals, and thus, residuals for four items across
two paths were allowed to correlate: DDPPI_Moral_4 ~~ DDPPI_Pruden_10; DDPPI_Conven_7 ~~ DDPPI_Person_15.

**Networks of Relationships Inventory, Social Provision Version (NRI-SPV)**

Detailed instructions, reliability of each factor, and factor loading of each item of NRI-SPV are listed below. For consistency, only the factor loadings and reliabilities of the scalar invariant model are presented below. The complete code and output for the MGCFA are available in the supplemental R-Markdown.

The following questions ask about your current relationship with your parents (or the people act most as your parental figures). Please answer the questions below on scale ranging from 1 "never or hardly at all" to 5 "always or extremely much." If the answer to a question varies depending on the parent considered, please select the answer with the higher score (e.g., select 4 “often or very much” rather than 3 “sometimes or somewhat”).

**Table S3.** Factor loadings from confirmatory factor analysis of the Networks of Relationships Inventory, Social Provision Version (NRI-SPV)

|  |  | **HK** | | **US** | |
| --- | --- | --- | --- | --- | --- |
|  | **No.** | **CR** | **Factor loading** | **CR** | **Factor loading** |
| **Factor 1: Conflict** |  | 0.916 |  | 0.899 |  |
| How often do you and your parents disagree and quarrel with each other? | NRI_SPV_Con_1 |  | 0.901 |  | 0.865 |
| How often do you and your parents get mad at or get in fights with each other? | NRI_SPV_Con_2 |  | 0.861 |  | 0.858 |
| How often do you and your parents argue with each other? | NRI_SPV_Con_3 |  | 0.895 |  | 0.875 |
| **Factor 2: Antagonism** |  | 0.888 |  | 0.852 |  |
| How much do you and your parents get on each other’s nerves? | NRI_SPV_Ant_4 |  | 0.813 |  | 0.811 |
| How much do you and your parents get annoyed with each other’s behavior? | NRI_SPV_Ant_5 |  | 0.895 |  | 0.805 |
| How much do you and your parents hassle or nag one another? | NRI_SPV_Ant_6 |  | 0.671 |  | 0.806 |
| **Factor 3: Affection** |  | 0.924 |  | 0.860 |  |
| How much do your parents like or love you? | NRI_SPV_Aff_7 |  | 0.883 |  | 0.866 |
| How much do your parents really care about you? | NRI_SPV_Aff_8 |  | 0.845 |  | 0.871 |
| How much do your parents have a strong feeling of affection (loving or liking) toward you? | NRI_SPV_Aff_9 |  | 0.804 |  | 0.831 |
| **Factor 4: Reassurance of Worth** |  | 0.890 |  | 0.875 |  |
| How much do your parents treat you like you’re admired and respected? | NRI_SPV_RS_10 |  | 0.884 |  | 0.894 |
| How much do your parents treat you like you’re good at many things? | NRI_SPV_RS_11 |  | 0.758 |  | 0.838 |
| How much do your parents like or approve of the things you do? | NRI_SPV_RS_12 |  | 0.801 |  | 0.767 |
| **Factor 5: Satisfaction** |  | 0.941 |  | 0.951 |  |
| How happy are you with your relationship with your parents? | NRI_SPV_Sat_13 |  | 0.928 |  | 0.960 |
| How much do you like the way things are between you and your parents? | NRI_SPV_Sat_14 |  | 0.810 |  | 0.919 |
| How satisfied are you with your relationship with your parents? | NRI_SPV_Sat_15 |  | 0.889 |  | 0.929 |
| **Scale Total** |  | 0.918 |  | 0.929 |  |

*Note.* 𝜔 = Composite Reliability (𝜔); Modification indices suggested the presence of correlated residuals, and thus, residuals for four items across
two paths were allowed to correlate: NRI_SPV_Aff_7 ~~ NRI_SPV_Aff_8; NRI_SPV_Aff_7 ~~ NRI_SPV_RS_11.
