# Supplementary material for: How parents leverage guilt and pride: A comparison of parental guilt and pride induction in Hong Kong and the United States
Source: J Res Adolesc. 2025 Dec 10;35(4):e70107. doi: 10.1111/jora.70107 (PMC12696220; doi:10.1111/jora.70107)
Supplement: Supplementary file 2 — Data S1: [file JORA-35-0-s005.pdf]

Descriptive Statistics and Group Comparisons

Corresponding Author

2024-10-25

This R Markdown document outlines the code used for conducting descriptive statistics and mixed measures ANOVA for group comparisons in our study. Further edits to enhance the aesthetics of the interaction plot were made using image editing software for the version presented in the manuscript.

Load packages

```
library(dplyr)
library(tidyr)
library(data.table) #function:as.data.table
library(vtable)#Table for descriptive
library(ggplot2) #Plot
library(effectsize)#Anova
library(emmeans) #Anova
```

Import data

```
datafile <- read.csv("D:\\R WM\\gipca data csv_with newly added data.csv")
```

Data Preparation

Set Region and Subject ID (Ref) as factors

```
datafile$Region<- as.factor(datafile$Region)
datafile$Ref<- as.factor(datafile$Ref)
```

Choose needed variables

```
datafile <- datafile %>%
  select(
    DDPGI_Moral_Mean, DDPGI_Conven_Mean,
    DDPGI_Pruden_Mean, DDPGI_Person_Mean,
    DDPPI_Moral_Mean, DDPPI_Conven_Mean,
    DDPPI_Pruden_Mean, DDPPI_Person_Mean,
    Parent_conflict_Mean, Parent_antag_Mean,
    Parent_affection_Mean, Parent_reassurance_Mean,
    Parent_satisfaction_Mean, Region, Ref
  )
```

Descriptive Statistics of Study Variables- Hong Kong

Variable	N	Mean	Std. Dev.	Min	Pctl. 25	Pctl. 75	Max
DDPGI_Moral_Mean	124	2.75	1.03	1	2	3.5	5
DDPGI_Conven_Mean	124	2.69	0.83	1	2	3.33	5
DDPGI_Pruden_Mean	124	2.78	1.16	1	1.67	4	5
DDPGI_Person_Mean	124	1.99	0.83	1	1.4	2.4	4.8
DDPPI_Moral_Mean	124	3.13	0.99	1	2.5	3.81	5
DDPPI_Conven_Mean	124	2.94	0.89	1	2.33	3.67	5
DDPPI_Pruden_Mean	124	3.19	0.99	1	2.67	4	5
DDPPI_Person_Mean	124	2.35	0.91	1	1.6	3	5
Parent_conflict_Mean	124	2.46	0.89	1	2	3	5
Parent_antag_Mean	124	2.77	0.87	1	2	3.33	5
Parent_affection_Mean	124	3.74	0.88	1.33	3	4.33	5
Parent_reassurance_Mean	124	3.42	0.89	1	3	4	5
Parent_satisfaction_Mean	124	3.47	0.86	1	3	4	5

Create subset data

Create subsets of the data to separate the US sample from the Hong Kong (HK) sample.

```
datafile_HK<-subset(datafile, Region == "HK")
datafile_US<-subset(datafile, Region == "US")
```

Descriptive Statistics: Hong Kong

```
# Create a new data frame excluding the "Ref" and "Region" columns
datafile_HK_subset <- datafile_HK[, !(names(datafile_HK) %in% c("Ref", "Region"))]

# Generate the vtable for the subsetted data
st(datafile_HK_subset,
   title = "Descriptive Statistics of Study Variables- Hong Kong",
   digits=2, numformat = NA)
```

Descriptive Statistics: United States

```
# Create a new data frame excluding the "Ref" and "Region" columns
datafile_US_subset <- datafile_US[, !(names(datafile_US) %in% c("Ref", "Region"))]

# Generate the vtable for the subsetted data
st(datafile_US_subset,
   title = "Descriptive Statistics of Study Variables- US",
   digits=2, numformat = NA)
```

Descriptive Statistics of Study Variables- US

Variable	N	Mean	Std. Dev.	Min	Pctl. 25	Pctl. 75	Max
DDPGI_Moral_Mean	142	3.8	0.94	1	3.25	4.5	5
DDPGI_Conven_Mean	142	3.75	1.01	1	3	4.67	5
DDPGI_Pruden_Mean	142	3.62	1.01	1	3	4.33	5
DDPGI_Person_Mean	142	2.54	1.13	1	1.45	3.4	5
DDPPI_Moral_Mean	142	3.92	1.03	1	3	4.75	5
DDPPI_Conven_Mean	142	3.69	1.04	1	3	4.33	5
DDPPI_Pruden_Mean	142	3.7	1.01	1	3	4.67	5
DDPPI_Person_Mean	142	2.88	1.03	1	2.2	3.6	5
Parent_conflict_Mean	142	2.39	0.98	1	1.67	3	5
Parent_antag_Mean	142	2.59	1.03	1	2	3.33	5
Parent_affection_Mean	142	4.48	0.87	1	4.33	5	5
Parent_reassurance_Mean	142	3.88	0.99	1	3.33	4.67	5
Parent_satisfaction_Mean	142	3.79	1.09	1	3	4.67	5

Mixed Measure ANOVA: Guilt Induction Domains * Region

This section presents the code for a mixed measures ANOVA to investigate guilt induction across cultures (United States vs. Hong Kong) and domains (moral, conventional, prudential, personal) on induction scores. It includes three sections: (1) Main Analysis, (2) Interaction Plot, and (3) Posthoc Analysis.

(1) Main Analysis

Transform data to long format

Guilt induction domains are labeled as 'DDPGI_Type' in the code for conciseness.

```
long_data <- datafile %>%
  pivot_longer(cols = c(DDPGI_Moral_Mean, DDPGI_Conven_Mean,
                        DDPGI_Pruden_Mean, DDPGI_Person_Mean),
               names_to = "DDPGI_Type",
               values_to = "Score")
```

Run the mixed measures ANOVA

```
anova_result <- aov(Score ~ DDPGI_Type * Region + Error(Ref/DDPGI_Type),
                    data = long_data)

# Summary of the ANOVA
summary(anova_result)
```

```
##
## Error: Ref
##            Df Sum Sq Mean Sq F value Pr(>F)
## Region      1  202.6  202.60    79.2 <2e-16 ***
## Residuals 264  675.3    2.56
```

```
## ---
## Signif. codes:  0 '***' 0.001 '**' 0.01 '*' 0.05 '.' 0.1 ' ' 1
##
## Error: Ref:DDPGI_Type
##                     Df Sum Sq Mean Sq F value   Pr(>F)
## DDPGI_Type           3  192.6   64.21 133.240  < 2e-16 ***
## DDPGI_Type:Region    3   11.5    3.82   7.929 3.25e-05 ***
## Residuals          792  381.7    0.48
## ---
## Signif. codes:  0 '***' 0.001 '**' 0.01 '*' 0.05 '.' 0.1 ' ' 1
```

Calculate effect sizes

```
effect_sizes <- eta_squared(anova_result)

# Display effect sizes
print(effect_sizes)
```

```
## # Effect Size for ANOVA (Type I)
##
## Group           |         Parameter | Eta2 (partial) |       95% CI
## -------------------------------------------------------------------
## Ref             |            Region |           0.23 | [0.16, 1.00]
## Ref:DDPGI_Type  |        DDPGI_Type |           0.34 | [0.29, 1.00]
## Ref:DDPGI_Type  | DDPGI_Type:Region |           0.03 | [0.01, 1.00]
##
## - One-sided CIs: upper bound fixed at [1.00].
```

```
str(effect_sizes)
```

```
## Classes 'effectsize_anova', 'effectsize_table', 'see_effectsize_table' and 'data.frame': 3 obs. of  [illegible]
##  $ Group       : chr  "Ref" "Ref:DDPGI_Type" "Ref:DDPGI_Type"
##  $ Parameter   : chr  "Region" "DDPGI_Type" "DDPGI_Type:Region"
##  $ Eta2_partial: num  0.2308 0.3354 0.0292
##  $ CI          : num  0.95 0.95 0.95
##  $ CI_low      : num  0.1614 0.2925 0.0109
##  $ CI_high     : num  1 1 1
##  - attr(*, "generalized")= logi FALSE
##  - attr(*, "ci")= num 0.95
##  - attr(*, "approximate")= logi FALSE
##  - attr(*, "alternative")= chr "greater"
##  - attr(*, "anova_type")= num 1
##  - attr(*, "ci_method")=List of 2
##   ..$ method      : chr "ncp"
##   ..$ distribution: chr "F"
```

(2) Interaction Plot

Convert long_data to a data.table

```
long_data_dt <- as.data.table(long_data)
```

Create plot_data using data.table syntax

```
plot_data <- long_data_dt[, .(Mean_Score = mean(Score, na.rm = TRUE)),
                          by = .(DDPGI_Type, Region)]
# Check the resulting plot_data
print(plot_data)
```

```
##           DDPGI_Type Region Mean_Score
##               <char> <fctr>      <num>
## 1:  DDPGI_Moral_Mean     HK   2.747984
## 2: DDPGI_Conven_Mean     HK   2.688253
## 3: DDPGI_Pruden_Mean     HK   2.779140
## 4: DDPGI_Person_Mean     HK   1.991935
## 5:  DDPGI_Moral_Mean     US   3.797535
## 6: DDPGI_Conven_Mean     US   3.746620
## 7: DDPGI_Pruden_Mean     US   3.624085
## 8: DDPGI_Person_Mean     US   2.538028
```

Adjust the levels and labels for DDPGI_Type

```
plot_data$DDPGI_Type <- factor(plot_data$DDPGI_Type,
                               levels = c("DDPGI_Moral_Mean",
                                          "DDPGI_Conven_Mean",
                                          "DDPGI_Pruden_Mean",
                                          "DDPGI_Person_Mean"),
                               labels = c("Moral", "Conventional",
                                          "Prudential", "Personal"))
```

Plot the interaction

```
ggplot(plot_data, aes(x = DDPGI_Type, y = Mean_Score, color = Region, group = Region)) +
  geom_line(aes(linetype = Region), size = 1) +  # Set size for better visibility
  geom_point(size = 3) +  # Set point size for better visibility
  labs(title = "Interaction between Guilt Induction and Region",
       x = "Guilt Induction",
       y = "Mean Score") +
  scale_color_manual(name = "Region",
                     labels = c("HK" = "Hong Kong", "US" = "United States"),
                     values = c("HK" = "black", "US" = "black")) +
  scale_linetype_manual(name = "Region",
```

```r
                        labels = c("HK" = "Hong Kong", "US" = "United States"),
                        values = c("HK" = "dotted", "US" = "solid")) +
  theme_minimal() +
  theme(
    plot.title = element_text(size = 16, face = "bold"),  # Title style
    axis.title.x = element_text(size = 14, face = "bold"),  # X-axis style
    axis.title.y = element_text(size = 14),  # Y-axis style
    axis.text.x = element_text(size = 12, margin = margin(b = 5)),  # X-axis style
    axis.text.y = element_text(size = 14, margin = margin(l = 5)),  # Y-axis style
    legend.text = element_text(size = 10, margin = margin(r = 5)),  # Legend text size
    legend.title = element_text(size = 10, margin = margin(r = 5)),  # Legend title size
     plot.margin = margin(t = 10, r = 20, b = 10, l = 20)
  ) +
  coord_cartesian(ylim = c(2, 4))  # Set y-axis limits
```

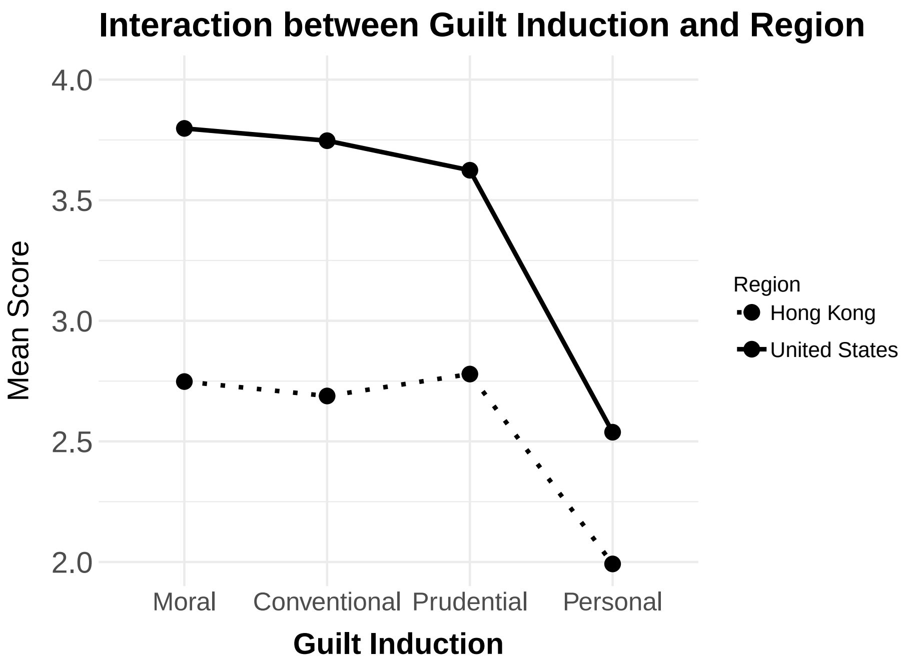

Frequency of Guilt Induction across Different Domains

(3) Posthoc analysis

Get estimated marginal means for the interaction

```r
emmeans_result <- emmeans(anova_result, ~ DDPGI_Type * Region)

emmeans_result
```

```
##  DDPGI_Type        Region emmean     SE  df lower.CL upper.CL
##  DDPGI_Conven_Mean HK       2.72 0.0879 606     2.55     2.89
```

```
##  DDPGI_Moral_Mean  HK       2.78 0.0879 606     2.60     2.95
##  DDPGI_Person_Mean HK       2.02 0.0879 606     1.85     2.19
##  DDPGI_Pruden_Mean HK       2.81 0.0879 606     2.64     2.98
##  DDPGI_Conven_Mean US       3.78 0.0858 564     3.61     3.94
##  DDPGI_Moral_Mean  US       3.83 0.0858 564     3.66     4.00
##  DDPGI_Person_Mean US       2.57 0.0858 564     2.40     2.74
##  DDPGI_Pruden_Mean US       3.65 0.0858 564     3.49     3.82
##
## Warning: EMMs are biased unless design is perfectly balanced
## Confidence level used: 0.95
```

Perform pairwise comparisons with Tukey adjustment

```r
posthoc_results <- pairs(emmeans_result, adjust = "tukey")
```

View the summary of the post-hoc results

```r
# Extract summary
summary_results <- summary(posthoc_results)

# Modify contrast labels
summary_results$contrast <- gsub("_Mean", "", summary_results$contrast)

# Display the modified summary
print(summary_results)
```

```
##  contrast                            estimate     SE  df t.ratio p.value
##  DDPGI_Conven HK - DDPGI_Moral HK     -0.0597 0.0882 792  -0.678  0.9976
##  DDPGI_Conven HK - DDPGI_Person HK     0.6963 0.0882 792   7.898  <.0001
##  DDPGI_Conven HK - DDPGI_Pruden HK    -0.0909 0.0882 792  -1.031  0.9697
##  DDPGI_Conven HK - DDPGI_Conven US    -1.0584 0.1230 585  -8.607  <.0001
##  DDPGI_Conven HK - DDPGI_Moral US     -1.1093 0.1230 585  -9.021  <.0001
##  DDPGI_Conven HK - DDPGI_Person US     0.1502 0.1230 585   1.222  0.9254
##  DDPGI_Conven HK - DDPGI_Pruden US    -0.9358 0.1230 585  -7.610  <.0001
##  DDPGI_Moral HK - DDPGI_Person HK      0.7560 0.0882 792   8.576  <.0001
##  DDPGI_Moral HK - DDPGI_Pruden HK     -0.0312 0.0882 792  -0.353  1.0000
##  DDPGI_Moral HK - DDPGI_Conven US     -0.9986 0.1230 585  -8.121  <.0001
##  DDPGI_Moral HK - DDPGI_Moral US      -1.0496 0.1230 585  -8.535  <.0001
##  DDPGI_Moral HK - DDPGI_Person US      0.2100 0.1230 585   1.707  0.6825
##  DDPGI_Moral HK - DDPGI_Pruden US     -0.8761 0.1230 585  -7.125  <.0001
##  DDPGI_Person HK - DDPGI_Pruden HK    -0.7872 0.0882 792  -8.929  <.0001
##  DDPGI_Person HK - DDPGI_Conven US    -1.7547 0.1230 585 -14.270  <.0001
##  DDPGI_Person HK - DDPGI_Moral US     -1.8056 0.1230 585 -14.684  <.0001
##  DDPGI_Person HK - DDPGI_Person US    -0.5461 0.1230 585  -4.441  0.0003
##  DDPGI_Person HK - DDPGI_Pruden US    -1.6321 0.1230 585 -13.273  <.0001
##  DDPGI_Pruden HK - DDPGI_Conven US    -0.9675 0.1230 585  -7.868  <.0001
##  DDPGI_Pruden HK - DDPGI_Moral US     -1.0184 0.1230 585  -8.282  <.0001
##  DDPGI_Pruden HK - DDPGI_Person US     0.2411 0.1230 585   1.961  0.5095
##  DDPGI_Pruden HK - DDPGI_Pruden US    -0.8449 0.1230 585  -6.871  <.0001
##  DDPGI_Conven US - DDPGI_Moral US     -0.0509 0.0824 792  -0.618  0.9986
```

```
##  DDPGI_Conven US - DDPGI_Person US   1.2086 0.0824 792  14.670  <.0001
##  DDPGI_Conven US - DDPGI_Pruden US   0.1225 0.0824 792   1.487  0.8143
##  DDPGI_Moral US - DDPGI_Person US    1.2595 0.0824 792  15.288  <.0001
##  DDPGI_Moral US - DDPGI_Pruden US    0.1735 0.0824 792   2.105  0.4123
##  DDPGI_Person US - DDPGI_Pruden US  -1.0861 0.0824 792 -13.182  <.0001
##
## P value adjustment: tukey method for comparing a family of 8 estimates
```

Custom contrast

```r
cc <- list(
  Person_non_Person_HK_US = c(1/3, 1/3, -1, 1/3, -1/3, -1/3, 1, -1/3)
  )

contrast(emmeans_result, cc, adjust = "tukey")
```

```
##  contrast                 estimate     SE  df t.ratio p.value
##  Person_non_Person_HK_US   -0.438 0.0985 792  -4.448  <.0001
```

Mixed Measure ANOVA: Pride Induction Domains * Region

This section presents the code for a mixed measures ANOVA to investigate pride induction across cultures (United States vs. Hong Kong) and domains (moral, conventional, prudential, personal) on induction scores. It includes three sections: (1) Main Analysis, (2) Interaction Plot, and (3) Posthoc Analysis.

(1) Main Analysis

Transform data to long format

Pride induction domains are labeled as 'DDPPI_Type' in the code for conciseness.

```
long_data <- datafile %>%
  pivot_longer(cols = c(DDPPI_Moral_Mean, DDPPI_Conven_Mean,
                        DDPPI_Pruden_Mean, DDPPI_Person_Mean),
               names_to = "DDPPI_Type",
               values_to = "Score")
```

Run the mixed measures ANOVA

```
anova_result <- aov(Score ~ DDPPI_Type * Region + Error(Ref/DDPPI_Type),
                    data = long_data)

# Summary of the ANOVA
summary(anova_result)
```

```
##
## Error: Ref
##            Df Sum Sq Mean Sq F value   Pr(>F)
## Region      1  111.6  111.62   35.69 7.45e-09 ***
## Residuals 264  825.6    3.13
## ---
## Signif. codes:  0 '***' 0.001 '**' 0.01 '*' 0.05 '.' 0.1 ' ' 1
##
## Error: Ref:DDPPI_Type
##                    Df Sum Sq Mean Sq F value  Pr(>F)
## DDPPI_Type          3 140.64   46.88 176.773 < 2e-16 ***
## DDPPI_Type:Region   3   4.13    1.38   5.192 0.00148 **
## Residuals         792 210.04    0.27
## ---
## Signif. codes:  0 '***' 0.001 '**' 0.01 '*' 0.05 '.' 0.1 ' ' 1
```

Calculate effect sizes

```
effect_sizes <- eta_squared(anova_result)

# Display effect sizes
print(effect_sizes)
```

```
## # Effect Size for ANOVA (Type I)
##
## Group           |          Parameter | Eta2 (partial) |       95% CI
## -------------------------------------------------------------------
## Ref             |             Region |           0.12 | [0.06, 1.00]
## Ref:DDPPI_Type  |         DDPPI_Type |           0.40 | [0.36, 1.00]
## Ref:DDPPI_Type  | DDPPI_Type:Region  |           0.02 | [0.00, 1.00]
##
## - One-sided CIs: upper bound fixed at [1.00].
```

```r
str(effect_sizes)
```

```
## Classes 'effectsize_anova', 'effectsize_table', 'see_effectsize_table' and 'data.frame': 3 obs. of
##  $ Group        : chr  "Ref" "Ref:DDPPI_Type" "Ref:DDPPI_Type"
##  $ Parameter    : chr  "Region" "DDPPI_Type" "DDPPI_Type:Region"
##  $ Eta2_partial: num  0.1191 0.4011 0.0193
##  $ CI           : num  0.95 0.95 0.95
##  $ CI_low       : num  0.06458 0.35933 0.00468
##  $ CI_high      : num  1 1 1
##  - attr(*, "generalized")= logi FALSE
##  - attr(*, "ci")= num 0.95
##  - attr(*, "approximate")= logi FALSE
##  - attr(*, "alternative")= chr "greater"
##  - attr(*, "anova_type")= num 1
##  - attr(*, "ci_method")=List of 2
##   ..$ method      : chr "ncp"
##   ..$ distribution: chr "F"
```

(2) Interaction Plot

Convert long_data to a data.table

```r
long_data_dt <- as.data.table(long_data)
```

Create plot_data using data.table syntax

```r
plot_data <- long_data_dt[, .(Mean_Score = mean(Score, na.rm = TRUE)),
                          by = .(DDPPI_Type, Region)]
# Check the resulting plot_data
print(plot_data)
```

```
##            DDPPI_Type Region Mean_Score
##                <char> <fctr>      <num>
## 1:  DDPPI_Moral_Mean     HK   3.131048
## 2: DDPPI_Conven_Mean     HK   2.935430
## 3: DDPPI_Pruden_Mean     HK   3.185591
## 4: DDPPI_Person_Mean     HK   2.346774
## 5:  DDPPI_Moral_Mean     US   3.919014
## 6: DDPPI_Conven_Mean     US   3.694859
## 7: DDPPI_Pruden_Mean     US   3.704577
## 8: DDPPI_Person_Mean     US   2.877465
```

Adjust the levels and labels for DDPPI_Type

```
plot_data$DDPPI_Type <- factor(plot_data$DDPPI_Type,
                               levels = c("DDPPI_Moral_Mean",
                                          "DDPPI_Conven_Mean",
                                          "DDPPI_Pruden_Mean",
                                          "DDPPI_Person_Mean"),
                               labels = c("Moral", "Conventional",
                                          "Prudential", "Personal"))
```

Plot the interaction

```
ggplot(plot_data, aes(x = DDPPI_Type, y = Mean_Score, color = Region, group = Region)) +
  geom_line(aes(linetype = Region), size = 1) +  # Set size for better visibility
  geom_point(size = 3) +  # Set point size for better visibility
  labs(title = "Interaction between Pride Induction and Region",
       x = "Pride Induction",
       y = "Mean Score") +
  scale_color_manual(name = "Region",
                     labels = c("HK" = "Hong Kong", "US" = "United States"),
                     values = c("HK" = "black", "US" = "black")) +
  scale_linetype_manual(name = "Region",
                        labels = c("HK" = "Hong Kong", "US" = "United States"),
                        values = c("HK" = "dotted", "US" = "solid")) +
  theme_minimal() +
  theme(
    plot.title = element_text(size = 16, face = "bold"),  # Title style
    axis.title.x = element_text(size = 14, face = "bold"),  # X-axis style
    axis.title.y = element_text(size = 14),  # Y-axis style
    axis.text.x = element_text(size = 12, margin = margin(b = 5)),  # X-axis style
    axis.text.y = element_text(size = 14, margin = margin(l = 5)),  # Y-axis style
    legend.text = element_text(size = 10, margin = margin(r = 5)),  # Legend text size
    legend.title = element_text(size = 10, margin = margin(r = 5)),  # Legend title size
    plot.margin = margin(t = 10, r = 20, b = 10, l = 20)
  ) +
  coord_cartesian(ylim = c(2, 4))  # Set y-axis limits
```

(3) Posthoc analysis

Get estimated marginal means for the interaction

```
emmeans_result <- emmeans(anova_result, ~ DDPPI_Type * Region)
```

Perform pairwise comparisons with Tukey adjustment

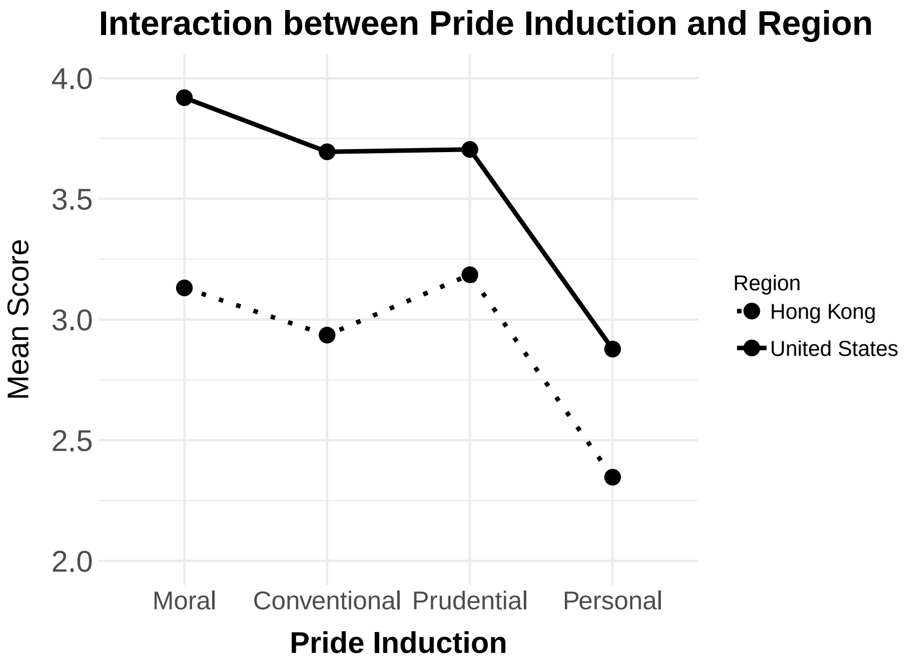

Frequency of Pride Induction across Different Domains

```
posthoc_results <- pairs(emmeans_result, adjust = "tukey")
```

View the summary of the post-hoc results

```
# Extract summary
summary_results <- summary(posthoc_results)

# Modify contrast labels
summary_results$contrast <- gsub("_Mean", "", summary_results$contrast)

# Display the modified summary
print(summary_results)
```

```
##  contrast                            estimate     SE  df t.ratio p.value
##  DDPPI_Conven HK - DDPPI_Moral HK  -0.19562 0.0654 792  -2.991  0.0574
##  DDPPI_Conven HK - DDPPI_Person HK  0.58866 0.0654 792   9.000  <.0001
##  DDPPI_Conven HK - DDPPI_Pruden HK -0.25016 0.0654 792  -3.825  0.0035
##  DDPPI_Conven HK - DDPPI_Conven US -0.75943 0.1217 407  -6.239  <.0001
##  DDPPI_Conven HK - DDPPI_Moral US  -0.98358 0.1217 407  -8.081  <.0001
##  DDPPI_Conven HK - DDPPI_Person US  0.05797 0.1217 407   0.476  0.9998
##  DDPPI_Conven HK - DDPPI_Pruden US -0.76915 0.1217 407  -6.319  <.0001
##  DDPPI_Moral HK - DDPPI_Person HK   0.78427 0.0654 792  11.991  <.0001
##  DDPPI_Moral HK - DDPPI_Pruden HK  -0.05454 0.0654 792  -0.834  0.9911
##  DDPPI_Moral HK - DDPPI_Conven US  -0.56381 0.1217 407  -4.632  0.0001
##  DDPPI_Moral HK - DDPPI_Moral US   -0.78797 0.1217 407  -6.474  <.0001
```

```
##  DDPPI_Moral HK - DDPPI_Person US   0.25358 0.1217 407   2.083  0.4275
##  DDPPI_Moral HK - DDPPI_Pruden US  -0.57353 0.1217 407  -4.712  0.0001
##  DDPPI_Person HK - DDPPI_Pruden HK -0.83882 0.0654 792 -12.825  <.0001
##  DDPPI_Person HK - DDPPI_Conven US -1.34808 0.1217 407 -11.075  <.0001
##  DDPPI_Person HK - DDPPI_Moral US  -1.57224 0.1217 407 -12.917  <.0001
##  DDPPI_Person HK - DDPPI_Person US -0.53069 0.1217 407  -4.360  0.0004
##  DDPPI_Person HK - DDPPI_Pruden US -1.35780 0.1217 407 -11.155  <.0001
##  DDPPI_Pruden HK - DDPPI_Conven US -0.50927 0.1217 407  -4.184  0.0009
##  DDPPI_Pruden HK - DDPPI_Moral US  -0.73342 0.1217 407  -6.025  <.0001
##  DDPPI_Pruden HK - DDPPI_Person US  0.30813 0.1217 407   2.531  0.1853
##  DDPPI_Pruden HK - DDPPI_Pruden US -0.51899 0.1217 407  -4.264  0.0007
##  DDPPI_Conven US - DDPPI_Moral US  -0.22415 0.0611 792  -3.668  0.0063
##  DDPPI_Conven US - DDPPI_Person US  0.81739 0.0611 792  13.374  <.0001
##  DDPPI_Conven US - DDPPI_Pruden US -0.00972 0.0611 792  -0.159  1.0000
##  DDPPI_Moral US - DDPPI_Person US   1.04155 0.0611 792  17.042  <.0001
##  DDPPI_Moral US - DDPPI_Pruden US   0.21444 0.0611 792   3.509  0.0112
##  DDPPI_Person US - DDPPI_Pruden US -0.82711 0.0611 792 -13.533  <.0001
##
## P value adjustment: tukey method for comparing a family of 8 estimates
```

Custom contrast

```
cc <- list(
  Person_non_Person_HK_US = c(1/3, 1/3, -1, 1/3, -1/3, -1/3, 1, -1/3)
  )

contrast(emmeans_result, cc, adjust = "tukey")
```

```
##  contrast                 estimate     SE  df t.ratio p.value
##  Person_non_Person_HK_US   -0.158 0.0731 792  -2.163  0.0308
```

Mixed Measure ANOVA: Relationship Quality features * Region

This section presents the code for a mixed measures ANOVA to investigate adolescent-parent relationship quality across cultures (United States vs. Hong Kong) and features (conflict, antagonism, affection, reassurance, satisfaction) on relationship scores. It includes three sections: (1) Main Analysis, (2) Interaction Plot, and (3) Posthoc Analysis.

(1) Main Analysis

Transform data to long format

Adolescent-parent relationship features are labeled as 'NRI_Type' in the code for conciseness.

```
long_data <- datafile %>%
  pivot_longer(cols = c(Parent_conflict_Mean, Parent_antag_Mean,
Parent_affection_Mean,  Parent_reassurance_Mean, Parent_satisfaction_Mean),
               names_to = "NRI_Type",
               values_to = "Score")
```

Run the mixed measures ANOVA

```
anova_result <- aov(Score ~ NRI_Type * Region + Error(Ref/NRI_Type),
                    data = long_data)

# Summary of the ANOVA
summary(anova_result)
```

```
##
## Error: Ref
##            Df Sum Sq Mean Sq F value   Pr(>F)
## Region      1  21.38  21.380   17.87 3.26e-05 ***
## Residuals 264 315.88   1.196
## ---
## Signif. codes:  0 '***' 0.001 '**' 0.01 '*' 0.05 '.' 0.1 ' ' 1
##
## Error: Ref:NRI_Type
##                   Df Sum Sq Mean Sq F value   Pr(>F)
## NRI_Type           4  559.0  139.76   172.0  < 2e-16 ***
## NRI_Type:Region    4   38.0    9.51    11.7 2.65e-09 ***
## Residuals       1056  858.2    0.81
## ---
## Signif. codes:  0 '***' 0.001 '**' 0.01 '*' 0.05 '.' 0.1 ' ' 1
```

Calculate effect sizes

```
effect_sizes <- eta_squared(anova_result)

# Display effect sizes
print(effect_sizes)
```

```
## # Effect Size for ANOVA (Type I)
##
## Group        |       Parameter | Eta2 (partial) |       95% CI
## ---------------------------------------------------------------
## Ref          |          Region |           0.06 | [0.02, 1.00]
## Ref:NRI_Type |        NRI_Type |           0.39 | [0.36, 1.00]
## Ref:NRI_Type | NRI_Type:Region |           0.04 | [0.02, 1.00]
##
## - One-sided CIs: upper bound fixed at [1.00].
```

```
str(effect_sizes)
```

```
## Classes 'effectsize_anova', 'effectsize_table', 'see_effectsize_table' and 'data.frame': 3 obs. of
##  $ Group       : chr  "Ref" "Ref:NRI_Type" "Ref:NRI_Type"
##  $ Parameter   : chr  "Region" "NRI_Type" "NRI_Type:Region"
##  $ Eta2_partial: num  0.0634 0.3945 0.0425
##  $ CI          : num  0.95 0.95 0.95
##  $ CI_low      : num  0.0241 0.358 0.0224
##  $ CI_high     : num  1 1 1
##  - attr(*, "generalized")= logi FALSE
##  - attr(*, "ci")= num 0.95
##  - attr(*, "approximate")= logi FALSE
##  - attr(*, "alternative")= chr "greater"
##  - attr(*, "anova_type")= num 1
##  - attr(*, "ci_method")=List of 2
##   ..$ method      : chr "ncp"
##   ..$ distribution: chr "F"
```

(2) Interaction Plot

Convert long_data to a data.table

```
long_data_dt <- as.data.table(long_data)
```

Create plot_data using data.table syntax

```
plot_data <- long_data_dt[, .(Mean_Score = mean(Score, na.rm = TRUE)),
                          by = .(NRI_Type, Region)]
# Check the resulting plot_data
print(plot_data)
```

```
##                     NRI_Type Region Mean_Score
##                       <char> <fctr>      <num>
##  1:      Parent_conflict_Mean     HK   2.464973
##  2:         Parent_antag_Mean     HK   2.768763
##  3:     Parent_affection_Mean     HK   3.739301
##  4:   Parent_reassurance_Mean     HK   3.422016
##  5: Parent_satisfaction_Mean     HK   3.470618
##  6:      Parent_conflict_Mean     US   2.394155
```

```
## 7:        Parent_antag_Mean     US   2.589296
## 8:     Parent_affection_Mean     US   4.476972
## 9:  Parent_reassurance_Mean     US   3.882535
## 10: Parent_satisfaction_Mean     US   3.793521
```

Adjust the levels and labels for NRI_Type

```
plot_data$NRI_Type <- factor(plot_data$NRI_Type,
                             levels = c("Parent_conflict_Mean",
                                        "Parent_antag_Mean",
                                        "Parent_affection_Mean",
                                        "Parent_reassurance_Mean",
                                        "Parent_satisfaction_Mean"),
                             labels = c("Conflict", "Antagonism", "Affection",
                                        "Reassurance", "Satisfaction"))
```

Plot the interaction

```
ggplot(plot_data, aes(x = NRI_Type, y = Mean_Score, color = Region, group = Region)) +
  geom_line(aes(linetype = Region), size = 1) +  # Set size for better visibility
  geom_point(size = 3) +  # Set point size for better visibility
  labs(title = "Interaction between Relationship Quality and Region",
       x = "Adolescent-Parent Relationship Quality",
       y = "Mean Score") +
  scale_color_manual(name = "Region",
                     labels = c("HK" = "Hong Kong", "US" = "United States"),
                     values = c("HK" = "black", "US" = "black")) +
  scale_linetype_manual(name = "Region",
                        labels = c("HK" = "Hong Kong", "US" = "United States"),
                        values = c("HK" = "dotted", "US" = "solid")) +
  theme_minimal() +
  theme(
    plot.title = element_text(size = 16, face = "bold"),  # Title style
    axis.title.x = element_text(size = 14, face = "bold"),  # X-axis style
    axis.title.y = element_text(size = 14),  # Y-axis style
    axis.text.x = element_text(size = 10, margin = margin(b = 5)),  # X-axis style
    axis.text.y = element_text(size = 14, margin = margin(l = 5)),  # Y-axis style
    legend.text = element_text(size = 10, margin = margin(r = 5)),  # Legend text size
    legend.title = element_text(size = 10, margin = margin(r = 5))  # Legend title size
  ) +
  coord_cartesian(ylim = c(2, 4.5))  # Set y-axis limits
```

(3) Posthoc analysis

Get estimated marginal means for the interaction

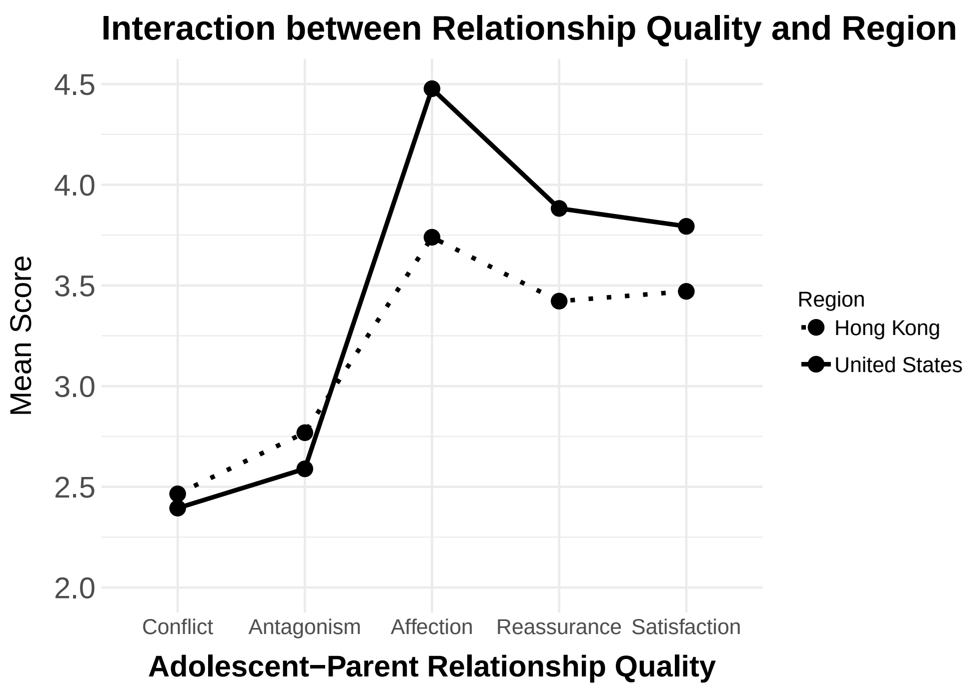

Adolescent-Parent Relationship Quality Across Different Features

```r
emmeans_result <- emmeans(anova_result, ~ NRI_Type * Region)
```

Perform pairwise comparisons with Tukey adjustment

```r
posthoc_results <- pairs(emmeans_result, adjust = "tukey")
```

View the summary of the post-hoc results

```r
# Extract summary
summary_results <- summary(posthoc_results)

# Modify contrast labels
summary_results$contrast <- gsub("Parent_|_Mean", "", summary_results$contrast)

# Display the modified summary
print(summary_results)
```

```
##  contrast                           estimate    SE   df t.ratio p.value
##  affection HK - antag HK              0.9705 0.114 1056   8.477  <.0001
##  affection HK - conflict HK           1.2743 0.114 1056  11.131  <.0001
##  affection HK - reassurance HK        0.3173 0.114 1056   2.771  0.1476
##  affection HK - satisfaction HK       0.2687 0.114 1056   2.347  0.3596
##  affection HK - affection US         -0.7377 0.116 1282  -6.364  <.0001
```

```
##  affection HK - antag US            1.1500 0.116 1282   9.921  <.0001
##  affection HK - conflict US         1.3451 0.116 1282  11.605  <.0001
##  affection HK - reassurance US     -0.1432 0.116 1282  -1.236  0.9666
##  affection HK - satisfaction US    -0.0542 0.116 1282  -0.468  1.0000
##  antag HK - conflict HK             0.3038 0.114 1056   2.653  0.1944
##  antag HK - reassurance HK         -0.6533 0.114 1056  -5.706  <.0001
##  antag HK - satisfaction HK        -0.7019 0.114 1056  -6.130  <.0001
##  antag HK - affection US           -1.7082 0.116 1282 -14.737  <.0001
##  antag HK - antag US                0.1795 0.116 1282   1.548  0.8722
##  antag HK - conflict US             0.3746 0.116 1282   3.232  0.0414
##  antag HK - reassurance US         -1.1138 0.116 1282  -9.609  <.0001
##  antag HK - satisfaction US        -1.0248 0.116 1282  -8.841  <.0001
##  conflict HK - reassurance HK      -0.9570 0.114 1056  -8.359  <.0001
##  conflict HK - satisfaction HK     -1.0056 0.114 1056  -8.784  <.0001
##  conflict HK - affection US        -2.0120 0.116 1282 -17.358  <.0001
##  conflict HK - antag US            -0.1243 0.116 1282  -1.073  0.9872
##  conflict HK - conflict US          0.0708 0.116 1282   0.611  0.9998
##  conflict HK - reassurance US      -1.4176 0.116 1282 -12.229  <.0001
##  conflict HK - satisfaction US     -1.3285 0.116 1282 -11.461  <.0001
##  reassurance HK - satisfaction HK  -0.0486 0.114 1056  -0.425  1.0000
##  reassurance HK - affection US     -1.0550 0.116 1282  -9.101  <.0001
##  reassurance HK - antag US          0.8327 0.116 1282   7.184  <.0001
##  reassurance HK - conflict US       1.0279 0.116 1282   8.867  <.0001
##  reassurance HK - reassurance US   -0.4605 0.116 1282  -3.973  0.0030
##  reassurance HK - satisfaction US  -0.3715 0.116 1282  -3.205  0.0449
##  satisfaction HK - affection US    -1.0064 0.116 1282  -8.682  <.0001
##  satisfaction HK - antag US         0.8813 0.116 1282   7.603  <.0001
##  satisfaction HK - conflict US      1.0765 0.116 1282   9.287  <.0001
##  satisfaction HK - reassurance US  -0.4119 0.116 1282  -3.554  0.0144
##  satisfaction HK - satisfaction US -0.3229 0.116 1282  -2.786  0.1423
##  affection US - antag US            1.8877 0.107 1056  17.644  <.0001
##  affection US - conflict US         2.0828 0.107 1056  19.468  <.0001
##  affection US - reassurance US      0.5944 0.107 1056   5.556  <.0001
##  affection US - satisfaction US     0.6835 0.107 1056   6.388  <.0001
##  antag US - conflict US             0.1951 0.107 1056   1.824  0.7199
##  antag US - reassurance US         -1.2932 0.107 1056 -12.088  <.0001
##  antag US - satisfaction US        -1.2042 0.107 1056 -11.256  <.0001
##  conflict US - reassurance US      -1.4884 0.107 1056 -13.912  <.0001
##  conflict US - satisfaction US     -1.3994 0.107 1056 -13.080  <.0001
##  reassurance US - satisfaction US   0.0890 0.107 1056   0.832  0.9981
##
## P value adjustment: tukey method for comparing a family of 10 estimates
```
