# Supplementary material for: How parents leverage guilt and pride: A comparison of parental guilt and pride induction in Hong Kong and the United States
Source: J Res Adolesc. 2025 Dec 10;35(4):e70107. doi: 10.1111/jora.70107 (PMC12696220; doi:10.1111/jora.70107)
Supplement: Supplementary file 3 — Data S2: [file JORA-35-0-s002.pdf]

Common Method Bias

Corresponding Author

2025-02-27

This R-markdown document presents the code utilized to test common method bias of the variables included in our study.

Load packages

```
library(psych) #for Harman's single-factor test
```

Import data

```
datafile <- read.csv("D:\\R WM\\gipca data csv_with newly added data.csv")
```

Harman's Single-Factor Test

Combine items for test

```
data_items <- datafile[, c(
  "DDPPI_Moral_1", "DDPPI_Moral_2", "DDPPI_Moral_3", "DDPPI_Moral_4",
  "DDPPI_Conven_5", "DDPPI_Conven_6", "DDPPI_Conven_7",
  "DDPPI_Pruden_8", "DDPPI_Pruden_9", "DDPPI_Pruden_10",
  "DDPGI_Moral_1", "DDPGI_Moral_2", "DDPGI_Moral_3", "DDPGI_Moral_4",
  "DDPGI_Conven_5", "DDPGI_Conven_6", "DDPGI_Conven_7",
  "DDPGI_Pruden_8", "DDPGI_Pruden_9", "DDPGI_Pruden_10",
  "DDPGI_Person_11", "DDPGI_Person_12", "DDPGI_Person_13", "DDPGI_Person_14", "DDPGI_Person_15",
  "NRI_SPV_Con_1", "NRI_SPV_Con_2", "NRI_SPV_Con_3",
  "NRI_SPV_Ant_4", "NRI_SPV_Ant_5", "NRI_SPV_Ant_6",
  "NRI_SPV_Aff_7", "NRI_SPV_Aff_8", "NRI_SPV_Aff_9",
  "NRI_SPV_RS_10", "NRI_SPV_RS_11", "NRI_SPV_RS_12",
  "NRI_SPV_Sat_13", "NRI_SPV_Sat_14", "NRI_SPV_Sat_15"
)]
```

Perform PCA (unrotated)

```r
pca_result <- principal(data_items, nfactors = ncol(data_items), rotate = "none")
```

Check variance explained by the first factor

```r
variance_explained <- (pca_result$values[1] / sum(pca_result$values)) * 100
cat("Variance explained by the first factor:", round(variance_explained, 2), "%\n")
```

```
## Variance explained by the first factor: 28.84 %
```

Output eigenvalues of all factors

```r
print(pca_result$values / sum(pca_result$values))
```

```
##  [1] 0.288446814 0.187790436 0.087391984 0.049044740 0.040082211 0.023493603
##  [7] 0.020794732 0.018657331 0.017563865 0.017385877 0.015619962 0.015310732
## [13] 0.014161460 0.013615442 0.013201999 0.011939061 0.011650058 0.011514840
## [19] 0.010672443 0.010434635 0.009454398 0.009244535 0.008965862 0.008321917
## [25] 0.007953315 0.007627810 0.007544804 0.007006058 0.006547820 0.006014863
## [31] 0.005663501 0.005355544 0.004933681 0.004915137 0.004588786 0.004383456
## [37] 0.004299611 0.003532085 0.002673904 0.002200686
```
