# Supplementary material for: How parents leverage guilt and pride: A comparison of parental guilt and pride induction in Hong Kong and the United States
Source: J Res Adolesc. 2025 Dec 10;35(4):e70107. doi: 10.1111/jora.70107 (PMC12696220; doi:10.1111/jora.70107)
Supplement: Supplementary file 4 — Data S3: [file JORA-35-0-s004.pdf]

Correlations and Density Plots

Corresponding Author

2024-10-25

This R-markdown document presents the code utilized to generate the correlation and density plot in our study. Further edits to enhance the aesthetics of the correlation plot, including adjustments to the labels and figure border, were made using image editing software for the version presented in the manuscript.

Load packages

```
library(plyr) #needed to run GGally
library(GGally) #function:ggpairs
library(psych) #function:corr.test
library(pander) #function: pander
library(cocor) #Compare correlations
```

Import data

```
datafile <- read.csv("D:\\R WM\\gipca data csv_with newly added data.csv")
```

Create subset data

Create subsets of the data to separate the US sample from the Hong Kong (HK) sample.

```
datafile_HK<-subset(datafile, Region == "HK")
datafile_US<-subset(datafile, Region == "US")
```

Domain-Differentiated Guilt Induction (DDPGI) and Adolescent-Parent Relationship Quality (NRI-SPV)

This section showcases the code used to create the correlation and density plots for the factors of the DDPGI and NRI-SPV scales.

Create dataframe for HK sample

```r
dGNRV_HK = data.frame(datafile_HK$DDPGI_Moral_Mean,datafile_HK$DDPGI_Conven_Mean,
                      datafile_HK$DDPGI_Pruden_Mean,datafile_HK$DDPGI_Person_Mean,
                      datafile_HK$Parent_conflict_Mean,datafile_HK$Parent_antag_Mean,
                      datafile_HK$Parent_affection_Mean,datafile_HK$Parent_reassurance_Mean,
                      datafile_HK$Parent_satisfaction_Mean)
```

Estimated density plot in diagonal

```r
my_fn <- function(data, mapping, ...){
  p <- ggplot(data =dGNRV_HK, mapping = mapping) +
    geom_point() + geom_smooth(method=lm, fill="blue", color="blue", ...)
  p }
```

Output r coefficients and P values

Parental Guilt Induction is labeled as 'GI' for conciseness.

```r
# Calculate correlation and p-values
dGNRV_HK.corr <- psych::corr.test(dGNRV_HK)
# Round p-values
rounded_p_values <- round(dGNRV_HK.corr$p, 3)
# Replace very small p-values with "< 0.0001"
formatted_p_values <- ifelse(rounded_p_values < 0.001, "< 0.001",
                              format(rounded_p_values, nsmall = 3,
                                     scientific = FALSE))
# Shorten variable labels by removing "datafile_" and "_Mean"
shorten_labels <- function(x) {
  gsub("datafile_", "", gsub("_Mean", "", x))
}
# Apply the function to row and column names
rownames(dGNRV_HK.corr$r) <- shorten_labels(rownames(dGNRV_HK.corr$r))
colnames(dGNRV_HK.corr$r) <- shorten_labels(colnames(dGNRV_HK.corr$r))
# Create a combined matrix with r and p values
combined_results <- matrix("", nrow = nrow(dGNRV_HK.corr$r), ncol = ncol(dGNRV_HK.corr$r))
combined_results[,] <- paste("r:", round(dGNRV_HK.corr$r, 3), "\np:", formatted_p_values)
# Set row and column names
rownames(combined_results) <- gsub("HK.|\\DDP", "",rownames(dGNRV_HK.corr$r))
colnames(combined_results) <- gsub("HK.|\\DDP", "",colnames(dGNRV_HK.corr$r))
# Output the combined results using pander
pander(combined_results)
```

Table continues below

	GI_Moral	GI_Conven
**GI_Moral**	r: 1 p: < 0.001	r: 0.608 p: < 0.001
**GI_Conven**	r: 0.608 p: < 0.001	r: 1 p: < 0.001
**GI_Pruden**	r: 0.804 p: < 0.001	r: 0.494 p: < 0.001
**GI_Person**	r: 0.379 p: < 0.001	r: 0.618 p: < 0.001
**Parent_conflict**	r: 0.065 p: 0.476	r: 0.154 p: 0.087

	GI_Moral	GI_Conven
**Parent__antag**	r: 0.023 p: 0.801	r: 0.158 p: 0.080
**Parent__affection**	r: 0.018 p: 0.843	r: 0.039 p: 0.666
**Parent__reassurance**	r: 0.025 p: 0.779	r: -0.076 p: 0.404
**Parent__satisfaction**	r: 0.097 p: 0.282	r: -0.033 p: 0.716

Table continues below

	GI_Pruden	GI_Person
**GI__Moral**	r: 0.804 p: < 0.001	r: 0.379 p: < 0.001
**GI__Conven**	r: 0.494 p: < 0.001	r: 0.618 p: < 0.001
**GI__Pruden**	r: 1 p: < 0.001	r: 0.294 p: 0.021
**GI__Person**	r: 0.294 p: 0.001	r: 1 p: < 0.001
**Parent__conflict**	r: 0.097 p: 0.285	r: 0.335 p: < 0.001
**Parent__antag**	r: 0.053 p: 0.559	r: 0.222 p: 0.013
**Parent__affection**	r: 0.023 p: 0.804	r: -0.145 p: 0.109
**Parent__reassurance**	r: 0.023 p: 0.797	r: -0.249 p: 0.005
**Parent__satisfaction**	r: 0.128 p: 0.158	r: -0.135 p: 0.135

Table continues below

	Parent__conflict	Parent__antag
**GI__Moral**	r: 0.065 p: 1.000	r: 0.023 p: 1.000
**GI__Conven**	r: 0.154 p: 1.000	r: 0.158 p: 1.000
**GI__Pruden**	r: 0.097 p: 1.000	r: 0.053 p: 1.000
**GI__Person**	r: 0.335 p: 0.004	r: 0.222 p: 0.264
**Parent__conflict**	r: 1 p: < 0.001	r: 0.816 p: < 0.001
**Parent__antag**	r: 0.816 p: < 0.001	r: 1 p: < 0.001
**Parent__affection**	r: -0.162 p: 0.073	r: -0.12 p: 0.184
**Parent__reassurance**	r: -0.293 p: 0.001	r: -0.309 p: < 0.001
**Parent__satisfaction**	r: -0.417 p: < 0.001	r: -0.414 p: < 0.001

Table continues below

	Parent__affection	Parent__reassurance
**GI__Moral**	r: 0.018 p: 1.000	r: 0.025 p: 1.000
**GI__Conven**	r: 0.039 p: 1.000	r: -0.076 p: 1.000
**GI__Pruden**	r: 0.023 p: 1.000	r: 0.023 p: 1.000
**GI__Person**	r: -0.145 p: 1.000	r: -0.249 p: 0.109
**Parent__conflict**	r: -0.162 p: 1.000	r: -0.293 p: 0.021
**Parent__antag**	r: -0.12 p: 1.000	r: -0.309 p: 0.011
**Parent__affection**	r: 1 p: < 0.001	r: 0.748 p: < 0.001
**Parent__reassurance**	r: 0.748 p: < 0.001	r: 1 p: < 0.001
**Parent__satisfaction**	r: 0.63 p: < 0.001	r: 0.664 p: < 0.001

	Parent__satisfaction
**GI__Moral**	r: 0.097 p: 1.000
**GI__Conven**	r: -0.033 p: 1.000
**GI__Pruden**	r: 0.128 p: 1.000
**GI__Person**	r: -0.135 p: 1.000
**Parent__conflict**	r: -0.417 p: < 0.001
**Parent__antag**	r: -0.414 p: < 0.001
**Parent__affection**	r: 0.63 p: < 0.001
**Parent__reassurance**	r: 0.664 p: < 0.001
**Parent__satisfaction**	r: 1 p: < 0.001

Create correlation and density plot for HK sample

```
ggpairs(dGNRV_HK,lower = list(continuous=my_fn))
```

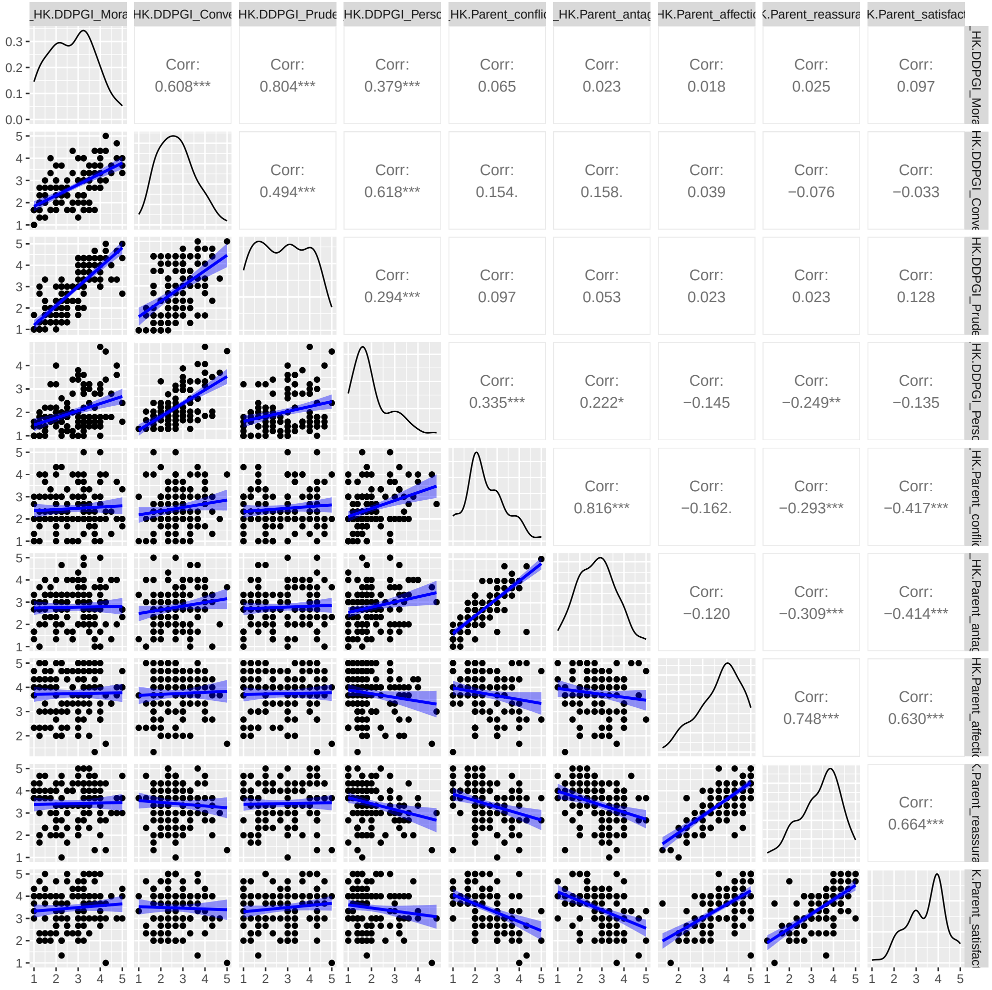

Correlations and Density Plots for DDPGI and NRI-SPV- Hong Kong Sample

Compare correlations between groups

Compare the correlations between four guilt induction domains and five features of adolescent-parent relationship quality in the United States and Hong Kong.

```
# DDPGI domains ~ NRI-SPV support features (Affection, Reassurance, Satisfaction)
correlations_HK <- c(0.018, 0.025, 0.097, 0.039, -0.076, -0.033,
                     0.023, 0.023, 0.128, -0.145, -0.249, -0.135)  # Corr HK
correlations_US <- c(-0.069, -0.091, -0.129, -0.165, -0.221, -0.245,
                     -0.119, -0.220, -0.170, -0.444, -0.600, -0.624)  # Corr US
# Sample sizes for each group
n1 <- 124  # Sample size for HK
n2 <- 142  # Sample size for US
# Define the correlation labels
correlation_labels <- c(
  "GI_Moral ~ Parent_affection", "GI_Moral ~ Parent_reassurance",
  "GI_Moral ~ Parent_satisfaction", "GI_Conven ~ Parent_affection",
  "GI_Conven ~ Parent_reassurance", "GI_Conven ~ Parent_satisfaction",
  "GI_Pruden ~ Parent_affection", "GI_Pruden ~ Parent_reassurance",
  "GI_Pruden ~ Parent_satisfaction", "GI_Person ~ Parent_affection",
  "GI_Person ~ Parent_reassurance", "GI_Person ~ Parent_satisfaction"
)
# Compare correlations and store results in a named list
results <- lapply(1:length(correlations_HK), function(i) {
  cocor.indep.groups(correlations_HK[i], correlations_US[i], n1, n2)
})
# Name the results
names(results) <- correlation_labels
# Print the results
for (label in correlation_labels) {
  cat("Comparison:", label, "\n")
  print(results[[label]])
}
```

```
## Comparison: GI_Moral ~ Parent_affection
##
##   Results of a comparison of two correlations based on independent groups
##
## Comparison between r1.jk = 0.018 and r2.hm = -0.069
## Difference: r1.jk - r2.hm = 0.087
## Group sizes: n1 = 124, n2 = 142
## Null hypothesis: r1.jk is equal to r2.hm
## Alternative hypothesis: r1.jk is not equal to r2.hm (two-sided)
## Alpha: 0.05
##
## fisher1925: Fisher's z (1925)
##   z = 0.7006, p-value = 0.4835
##   Null hypothesis retained
##
## zou2007: Zou's (2007) confidence interval
##   95% confidence interval for r1.jk - r2.hm: -0.1554 0.3261
##   Null hypothesis retained (Interval includes 0)
##
## Comparison: GI_Moral ~ Parent_reassurance
```

```
##
##   Results of a comparison of two correlations based on independent groups
##
## Comparison between r1.jk = 0.025 and r2.hm = -0.091
## Difference: r1.jk - r2.hm = 0.116
## Group sizes: n1 = 124, n2 = 142
## Null hypothesis: r1.jk is equal to r2.hm
## Alternative hypothesis: r1.jk is not equal to r2.hm (two-sided)
## Alpha: 0.05
##
## fisher1925: Fisher's z (1925)
##   z = 0.9351, p-value = 0.3498
##   Null hypothesis retained
##
## zou2007: Zou's (2007) confidence interval
##   95% confidence interval for r1.jk - r2.hm: -0.1265 0.3541
##   Null hypothesis retained (Interval includes 0)
##
## Comparison: GI_Moral ~ Parent_satisfaction
##
##   Results of a comparison of two correlations based on independent groups
##
## Comparison between r1.jk = 0.097 and r2.hm = -0.129
## Difference: r1.jk - r2.hm = 0.226
## Group sizes: n1 = 124, n2 = 142
## Null hypothesis: r1.jk is equal to r2.hm
## Alternative hypothesis: r1.jk is not equal to r2.hm (two-sided)
## Alpha: 0.05
##
## fisher1925: Fisher's z (1925)
##   z = 1.8260, p-value = 0.0679
##   Null hypothesis retained
##
## zou2007: Zou's (2007) confidence interval
##   95% confidence interval for r1.jk - r2.hm: -0.0168 0.4598
##   Null hypothesis retained (Interval includes 0)
##
## Comparison: GI_Conven ~ Parent_affection
##
##   Results of a comparison of two correlations based on independent groups
##
## Comparison between r1.jk = 0.039 and r2.hm = -0.165
## Difference: r1.jk - r2.hm = 0.204
## Group sizes: n1 = 124, n2 = 142
## Null hypothesis: r1.jk is equal to r2.hm
## Alternative hypothesis: r1.jk is not equal to r2.hm (two-sided)
## Alpha: 0.05
##
## fisher1925: Fisher's z (1925)
##   z = 1.6532, p-value = 0.0983
##   Null hypothesis retained
##
## zou2007: Zou's (2007) confidence interval
##   95% confidence interval for r1.jk - r2.hm: -0.0380 0.4383
```

```
##   Null hypothesis retained (Interval includes 0)
##
## Comparison: GI_Conven ~ Parent_reassurance
##
##   Results of a comparison of two correlations based on independent groups
##
## Comparison between r1.jk = -0.076 and r2.hm = -0.221
## Difference: r1.jk - r2.hm = 0.145
## Group sizes: n1 = 124, n2 = 142
## Null hypothesis: r1.jk is equal to r2.hm
## Alternative hypothesis: r1.jk is not equal to r2.hm (two-sided)
## Alpha: 0.05
##
## fisher1925: Fisher's z (1925)
##   z = 1.1949, p-value = 0.2321
##   Null hypothesis retained
##
## zou2007: Zou's (2007) confidence interval
##   95% confidence interval for r1.jk - r2.hm: -0.0924 0.3783
##   Null hypothesis retained (Interval includes 0)
##
## Comparison: GI_Conven ~ Parent_satisfaction
##
##   Results of a comparison of two correlations based on independent groups
##
## Comparison between r1.jk = -0.033 and r2.hm = -0.245
## Difference: r1.jk - r2.hm = 0.212
## Group sizes: n1 = 124, n2 = 142
## Null hypothesis: r1.jk is equal to r2.hm
## Alternative hypothesis: r1.jk is not equal to r2.hm (two-sided)
## Alpha: 0.05
##
## fisher1925: Fisher's z (1925)
##   z = 1.7459, p-value = 0.0808
##   Null hypothesis retained
##
## zou2007: Zou's (2007) confidence interval
##   95% confidence interval for r1.jk - r2.hm: -0.0261 0.4434
##   Null hypothesis retained (Interval includes 0)
##
## Comparison: GI_Pruden ~ Parent_affection
##
##   Results of a comparison of two correlations based on independent groups
##
## Comparison between r1.jk = 0.023 and r2.hm = -0.119
## Difference: r1.jk - r2.hm = 0.142
## Group sizes: n1 = 124, n2 = 142
## Null hypothesis: r1.jk is equal to r2.hm
## Alternative hypothesis: r1.jk is not equal to r2.hm (two-sided)
## Alpha: 0.05
##
## fisher1925: Fisher's z (1925)
##   z = 1.1467, p-value = 0.2515
##   Null hypothesis retained
```

```
##
## zou2007: Zou's (2007) confidence interval
##   95% confidence interval for r1.jk - r2.hm: -0.1004 0.3790
##   Null hypothesis retained (Interval includes 0)
##
## Comparison: GI_Pruden ~ Parent_reassurance
##
##   Results of a comparison of two correlations based on independent groups
##
## Comparison between r1.jk = 0.023 and r2.hm = -0.22
## Difference: r1.jk - r2.hm = 0.243
## Group sizes: n1 = 124, n2 = 142
## Null hypothesis: r1.jk is equal to r2.hm
## Alternative hypothesis: r1.jk is not equal to r2.hm (two-sided)
## Alpha: 0.05
##
## fisher1925: Fisher's z (1925)
##   z = 1.9839, p-value = 0.0473
##   Null hypothesis rejected
##
## zou2007: Zou's (2007) confidence interval
##   95% confidence interval for r1.jk - r2.hm: 0.0027 0.4747
##   Null hypothesis rejected (Interval does not include 0)
##
## Comparison: GI_Pruden ~ Parent_satisfaction
##
##   Results of a comparison of two correlations based on independent groups
##
## Comparison between r1.jk = 0.128 and r2.hm = -0.17
## Difference: r1.jk - r2.hm = 0.298
## Group sizes: n1 = 124, n2 = 142
## Null hypothesis: r1.jk is equal to r2.hm
## Alternative hypothesis: r1.jk is not equal to r2.hm (two-sided)
## Alpha: 0.05
##
## fisher1925: Fisher's z (1925)
##   z = 2.4159, p-value = 0.0157
##   Null hypothesis rejected
##
## zou2007: Zou's (2007) confidence interval
##   95% confidence interval for r1.jk - r2.hm: 0.0560 0.5282
##   Null hypothesis rejected (Interval does not include 0)
##
## Comparison: GI_Person ~ Parent_affection
##
##   Results of a comparison of two correlations based on independent groups
##
## Comparison between r1.jk = -0.145 and r2.hm = -0.444
## Difference: r1.jk - r2.hm = 0.299
## Group sizes: n1 = 124, n2 = 142
## Null hypothesis: r1.jk is equal to r2.hm
## Alternative hypothesis: r1.jk is not equal to r2.hm (two-sided)
## Alpha: 0.05
##
```

```
## fisher1925: Fisher's z (1925)
##   z = 2.6636, p-value = 0.0077
##   Null hypothesis rejected
##
## zou2007: Zou's (2007) confidence interval
##   95% confidence interval for r1.jk - r2.hm: 0.0783 0.5148
##   Null hypothesis rejected (Interval does not include 0)
##
## Comparison: GI_Person ~ Parent_reassurance
##
##   Results of a comparison of two correlations based on independent groups
##
## Comparison between r1.jk = -0.249 and r2.hm = -0.6
## Difference: r1.jk - r2.hm = 0.351
## Group sizes: n1 = 124, n2 = 142
## Null hypothesis: r1.jk is equal to r2.hm
## Alternative hypothesis: r1.jk is not equal to r2.hm (two-sided)
## Alpha: 0.05
##
## fisher1925: Fisher's z (1925)
##   z = 3.5292, p-value = 0.0004
##   Null hypothesis rejected
##
## zou2007: Zou's (2007) confidence interval
##   95% confidence interval for r1.jk - r2.hm: 0.1541 0.5488
##   Null hypothesis rejected (Interval does not include 0)
##
## Comparison: GI_Person ~ Parent_satisfaction
##
##   Results of a comparison of two correlations based on independent groups
##
## Comparison between r1.jk = -0.135 and r2.hm = -0.624
## Difference: r1.jk - r2.hm = 0.489
## Group sizes: n1 = 124, n2 = 142
## Null hypothesis: r1.jk is equal to r2.hm
## Alternative hypothesis: r1.jk is not equal to r2.hm (two-sided)
## Alpha: 0.05
##
## fisher1925: Fisher's z (1925)
##   z = 4.7912, p-value = 0.0000
##   Null hypothesis rejected
##
## zou2007: Zou's (2007) confidence interval
##   95% confidence interval for r1.jk - r2.hm: 0.2861 0.6884
##   Null hypothesis rejected (Interval does not include 0)
```

Create dataframe for US sample

```
dGNRV_US = data.frame(datafile_US$DDPGI_Moral_Mean,datafile_US$DDPGI_Conven_Mean,
                      datafile_US$DDPGI_Pruden_Mean,datafile_US$DDPGI_Person_Mean,
                      datafile_US$Parent_conflict_Mean,datafile_US$Parent_antag_Mean,
                      datafile_US$Parent_affection_Mean,datafile_US$Parent_reassurance_Mean,
                      datafile_US$Parent_satisfaction_Mean)
```

Estimated density plot in diagonal

```
my_fn <- function(data, mapping, ...){
  p <- ggplot(data = dGNRV_US, mapping = mapping) +
    geom_point() + geom_smooth(method=lm, fill="purple", color="purple", ...)
  p }
```

Output r coefficients and P values

Parental Guilt Induction is labeled as 'GI' for conciseness.

```
# Calculate correlation and p-values
dGNRV_US.corr <- psych::corr.test(dGNRV_US)
# Round p-values
rounded_p_values <- round(dGNRV_US.corr$p, 3)
# Replace very small p-values with "< 0.0001"
formatted_p_values <- ifelse(rounded_p_values < 0.001, "< 0.001",
                             format(rounded_p_values, nsmall = 3,
                                    scientific = FALSE))
# Shorten variable labels by removing "datafile_" and "_Mean"
shorten_labels <- function(x) {
  gsub("datafile_", "", gsub("_Mean", "", x))
}
# Apply the function to row and column names
rownames(dGNRV_US.corr$r) <- shorten_labels(rownames(dGNRV_US.corr$r))
colnames(dGNRV_US.corr$r) <- shorten_labels(colnames(dGNRV_US.corr$r))
# Create a combined matrix with r and p values
combined_results <- matrix("", nrow = nrow(dGNRV_US.corr$r), ncol = ncol(dGNRV_US.corr$r))
combined_results[,] <- paste("r:", round(dGNRV_US.corr$r, 3), "\np:", formatted_p_values)
# Set row and column names
rownames(combined_results) <- gsub("US.|\\DDP", "",rownames(dGNRV_US.corr$r))
colnames(combined_results) <- gsub("US.|\\DDP", "",colnames(dGNRV_US.corr$r))
# Output the combined results using pander
pander(combined_results)
```

Table continues below

	GI_Moral	GI_Conven
**GI_Moral**	r: 1 p: < 0.001	r: 0.671 p: < 0.001
**GI_Conven**	r: 0.671 p: < 0.001	r: 1 p: < 0.001

	GI__Moral	GI__Conven
**GI__Pruden**	r: 0.635 p: < 0.001	r: 0.61 p: < 0.001
**GI__Person**	r: 0.285 p: 0.001	r: 0.495 p: < 0.001
**Parent__conflict**	r: -0.04 p: 0.637	r: 0.095 p: 0.263
**Parent__antag**	r: 0.027 p: 0.747	r: 0.149 p: 0.077
**Parent__affection**	r: -0.069 p: 0.414	r: -0.165 p: 0.049
**Parent__reassurance**	r: -0.091 p: 0.281	r: -0.221 p: 0.008
**Parent__satisfaction**	r: -0.129 p: 0.126	r: -0.245 p: 0.003

Table continues below

	GI__Pruden	GI__Person
**GI__Moral**	r: 0.635 p: < 0.001	r: 0.285 p: 0.010
**GI__Conven**	r: 0.61 p: < 0.001	r: 0.495 p: < 0.001
**GI__Pruden**	r: 1 p: < 0.001	r: 0.439 p: < 0.001
**GI__Person**	r: 0.439 p: < 0.001	r: 1 p: < 0.001
**Parent__conflict**	r: 0.06 p: 0.479	r: 0.38 p: < 0.001
**Parent__antag**	r: 0.078 p: 0.357	r: 0.393 p: < 0.001
**Parent__affection**	r: -0.119 p: 0.158	r: -0.444 p: < 0.001
**Parent__reassurance**	r: -0.22 p: 0.008	r: -0.6 p: < 0.001
**Parent__satisfaction**	r: -0.17 p: 0.043	r: -0.624 p: < 0.001

Table continues below

	Parent__conflict	Parent__antag
**GI__Moral**	r: -0.04 p: 1.000	r: 0.027 p: 1.000
**GI__Conven**	r: 0.095 p: 1.000	r: 0.149 p: 0.767
**GI__Pruden**	r: 0.06 p: 1.000	r: 0.078 p: 1.000
**GI__Person**	r: 0.38 p: < 0.001	r: 0.393 p: < 0.001
**Parent__conflict**	r: 1 p: < 0.001	r: 0.819 p: < 0.001
**Parent__antag**	r: 0.819 p: < 0.001	r: 1 p: < 0.001
**Parent__affection**	r: -0.232 p: 0.005	r: -0.279 p: 0.001
**Parent__reassurance**	r: -0.401 p: < 0.001	r: -0.417 p: < 0.001
**Parent__satisfaction**	r: -0.456 p: < 0.001	r: -0.467 p: < 0.001

Table continues below

	Parent__affection	Parent__reassurance
**GI__Moral**	r: -0.069 p: 1.000	r: -0.091 p: 1.000
**GI__Conven**	r: -0.165 p: 0.543	r: -0.221 p: 0.116
**GI__Pruden**	r: -0.119 p: 1.000	r: -0.22 p: 0.116
**GI__Person**	r: -0.444 p: < 0.001	r: -0.6 p: < 0.001
**Parent__conflict**	r: -0.232 p: 0.081	r: -0.401 p: < 0.001
**Parent__antag**	r: -0.279 p: 0.013	r: -0.417 p: < 0.001
**Parent__affection**	r: 1 p: < 0.001	r: 0.742 p: < 0.001
**Parent__reassurance**	r: 0.742 p: < 0.001	r: 1 p: < 0.001
**Parent__satisfaction**	r: 0.718 p: < 0.001	r: 0.74 p: < 0.001

	Parent_satisfaction
**GI_Moral**	r: -0.129 p: 1.000
**GI_Conven**	r: -0.245 p: 0.052
**GI_Pruden**	r: -0.17 p: 0.522
**GI_Person**	r: -0.624 p: < 0.001
**Parent_conflict**	r: -0.456 p: < 0.001
**Parent_antag**	r: -0.467 p: < 0.001
**Parent_affection**	r: 0.718 p: < 0.001
**Parent_reassurance**	r: 0.74 p: < 0.001
**Parent_satisfaction**	r: 1 p: < 0.001

Create correlation and density plot for US sample

```
ggpairs(dGNRV_US,lower = list(continuous=my_fn))
```

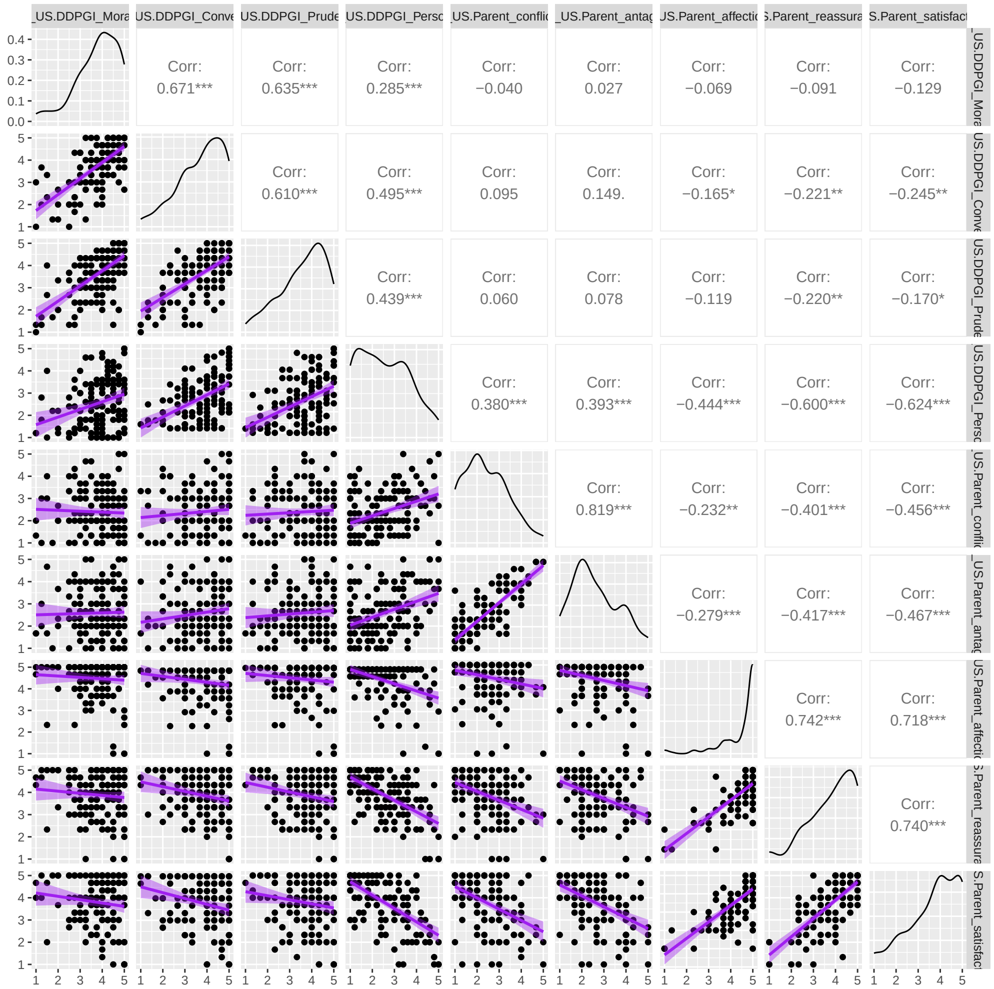

Correlations and Density Plots for DDPGI and NRI-SPV- US Sample

Domain-Differentiated Pride Induction (DDPPI) and Adolescent-Parent Relationship Quality (NRI-SPV)

This section showcases the code used to create the correlation and density plots for the factors of the DDPPI and NRI-SPV scales.

Create dataframe for HK sample

```
dPNRV_HK = data.frame(datafile_HK$DDPPI_Moral_Mean,datafile_HK$DDPPI_Conven_Mean,
                      datafile_HK$DDPPI_Pruden_Mean,datafile_HK$DDPPI_Person_Mean,
                      datafile_HK$Parent_conflict_Mean,datafile_HK$Parent_antag_Mean,
                      datafile_HK$Parent_affection_Mean,datafile_HK$Parent_reassurance_Mean,
                      datafile_HK$Parent_satisfaction_Mean)
```

Estimated density plot in diagonal

```
my_fn <- function(data, mapping, ...){
  p <- ggplot(data =dPNRV_HK, mapping = mapping) +
    geom_point() + geom_smooth(method=lm, fill="blue", color="blue", ...)
  p }
```

Output r coefficients and P values

Parental Pride Induction is labeled 'PI' in the output for conciseness.

```
# Calculate correlation and p-values
dPNRV_HK.corr <- psych::corr.test(dPNRV_HK)
# Round p-values
rounded_p_values <- round(dPNRV_HK.corr$p, 3)
# Replace very small p-values with "< 0.0001"
formatted_p_values <- ifelse(rounded_p_values < 0.001, "< 0.001",
                             format(rounded_p_values, nsmall = 3,
                                    scientific = FALSE))
# Shorten variable labels by removing "datafile_" and "_Mean"
shorten_labels <- function(x) {
  gsub("datafile_", "", gsub("_Mean", "", x))
}
# Apply the function to row and column names
rownames(dPNRV_HK.corr$r) <- shorten_labels(rownames(dPNRV_HK.corr$r))
colnames(dPNRV_HK.corr$r) <- shorten_labels(colnames(dPNRV_HK.corr$r))
# Create a combined matrix with r and p values
combined_results <- matrix("", nrow = nrow(dPNRV_HK.corr$r), ncol = ncol(dPNRV_HK.corr$r))
combined_results[,] <- paste("r:", round(dPNRV_HK.corr$r, 3), "\np:", formatted_p_values)
# Set row and column names
rownames(combined_results) <- gsub("HK.|\\DDP", "", rownames(dPNRV_HK.corr$r))
colnames(combined_results) <- gsub("HK.|\\DDP", "", colnames(dPNRV_HK.corr$r))
# Output the combined results using pander
pander(combined_results)
```

Table continues below

	PI_Moral	PI_Conven
**PI_Moral**	r: 1 p: < 0.001	r: 0.804 p: < 0.001
**PI_Conven**	r: 0.804 p: < 0.001	r: 1 p: < 0.001
**PI_Pruden**	r: 0.745 p: < 0.001	r: 0.739 p: < 0.001
**PI_Person**	r: 0.751 p: < 0.001	r: 0.724 p: < 0.001
**Parent_conflict**	r: -0.076 p: 0.399	r: -0.071 p: 0.433
**Parent_antag**	r: -0.165 p: 0.067	r: -0.091 p: 0.313
**Parent_affection**	r: 0.477 p: < 0.001	r: 0.418 p: < 0.001
**Parent_reassurance**	r: 0.48 p: < 0.001	r: 0.363 p: < 0.001
**Parent_satisfaction**	r: 0.392 p: < 0.001	r: 0.372 p: < 0.001

Table continues below

	PI_Pruden	PI_Person
**PI_Moral**	r: 0.745 p: < 0.001	r: 0.751 p: < 0.001
**PI_Conven**	r: 0.739 p: < 0.001	r: 0.724 p: < 0.001
**PI_Pruden**	r: 1 p: < 0.001	r: 0.661 p: < 0.001
**PI_Person**	r: 0.661 p: < 0.001	r: 1 p: < 0.001
**Parent_conflict**	r: 0.055 p: 0.545	r: 0.063 p: 0.486
**Parent_antag**	r: 0.017 p: 0.847	r: -0.086 p: 0.345
**Parent_affection**	r: 0.393 p: < 0.001	r: 0.385 p: < 0.001
**Parent_reassurance**	r: 0.36 p: < 0.001	r: 0.382 p: < 0.001
**Parent_satisfaction**	r: 0.239 p: 0.007	r: 0.267 p: 0.003

Table continues below

	Parent_conflict	Parent_antag
**PI_Moral**	r: -0.076 p: 1.000	r: -0.165 p: 0.674
**PI_Conven**	r: -0.071 p: 1.000	r: -0.091 p: 1.000
**PI_Pruden**	r: 0.055 p: 1.000	r: 0.017 p: 1.000
**PI_Person**	r: 0.063 p: 1.000	r: -0.086 p: 1.000
**Parent_conflict**	r: 1 p: < 0.001	r: 0.816 p: < 0.001
**Parent_antag**	r: 0.816 p: < 0.001	r: 1 p: < 0.001
**Parent_affection**	r: -0.162 p: 0.073	r: -0.12 p: 0.184
**Parent_reassurance**	r: -0.293 p: 0.001	r: -0.309 p: < 0.001
**Parent_satisfaction**	r: -0.417 p: < 0.001	r: -0.414 p: < 0.001

Table continues below

	Parent_affection	Parent_reassurance
**PI_Moral**	r: 0.477 p: < 0.001	r: 0.48 p: < 0.001
**PI_Conven**	r: 0.418 p: < 0.001	r: 0.363 p: 0.001
**PI_Pruden**	r: 0.393 p: < 0.001	r: 0.36 p: 0.001
**PI_Person**	r: 0.385 p: < 0.001	r: 0.382 p: < 0.001
**Parent_conflict**	r: -0.162 p: 0.674	r: -0.293 p: 0.012
**Parent_antag**	r: -0.12 p: 1.000	r: -0.309 p: 0.007

	Parent__affection	Parent__reassurance
**Parent__affection**	r: 1 p: < 0.001	r: 0.748 p: < 0.001
**Parent__reassurance**	r: 0.748 p: < 0.001	r: 1 p: < 0.001
**Parent__satisfaction**	r: 0.63 p: < 0.001	r: 0.664 p: < 0.001

	Parent__satisfaction
**PI__Moral**	r: 0.392 p: < 0.001
**PI__Conven**	r: 0.372 p: < 0.001
**PI__Pruden**	r: 0.239 p: 0.082
**PI__Person**	r: 0.267 p: 0.033
**Parent__conflict**	r: -0.417 p: < 0.001
**Parent__antag**	r: -0.414 p: < 0.001
**Parent__affection**	r: 0.63 p: < 0.001
**Parent__reassurance**	r: 0.664 p: < 0.001
**Parent__satisfaction**	r: 1 p: < 0.001

Create correlation and density plot for HK sample

```
ggpairs(dPNRV_HK,lower = list(continuous=my_fn))
```

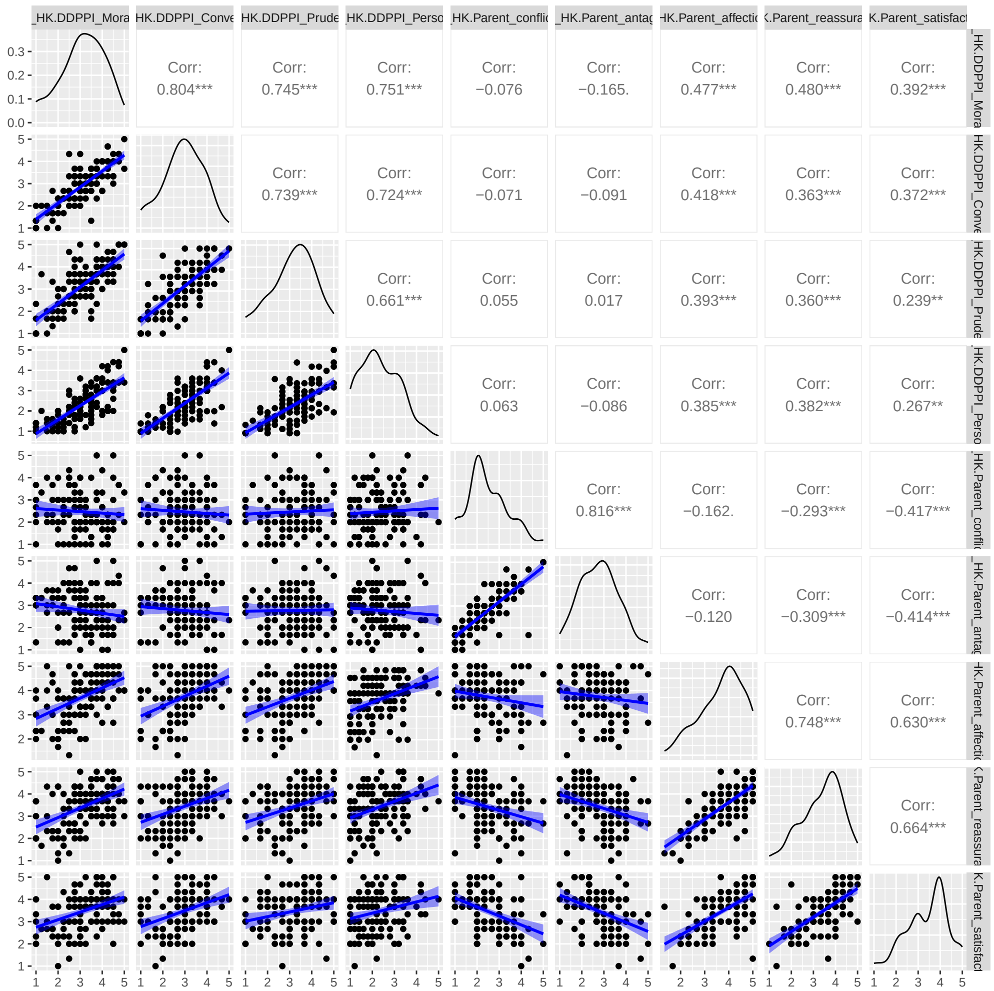

Correlations and Density Plots for DDPPI and NRI-SPV- Hong Kong Sample

Create dataframe for US sample

```
dPNRV_US = data.frame(datafile_US$DDPPI_Moral_Mean,datafile_US$DDPPI_Conven_Mean,
                      datafile_US$DDPPI_Pruden_Mean,datafile_US$DDPPI_Person_Mean,
                      datafile_US$Parent_conflict_Mean,datafile_US$Parent_antag_Mean,
                      datafile_US$Parent_affection_Mean,datafile_US$Parent_reassurance_Mean,
                      datafile_US$Parent_satisfaction_Mean)
```

Estimated density plot in diagonal

```
my_fn <- function(data, mapping, ...){
  p <- ggplot(data = dPNRV_US, mapping = mapping) +
    geom_point() + geom_smooth(method=lm, fill="purple", color="purple", ...)
  p }
```

Output r coefficients and P values

Parental Pride Induction is labeled as 'PI' in the output for conciseness.

```
# Calculate correlation and p-values
dPNRV_US.corr <- psych::corr.test(dPNRV_US)
# Round p-values
rounded_p_values <- round(dPNRV_US.corr$p, 3)
# Replace very small p-values with "< 0.0001"
formatted_p_values <- ifelse(rounded_p_values < 0.001, "< 0.001",
                             format(rounded_p_values, nsmall = 3,
                                    scientific = FALSE))
# Shorten variable labels by removing "datafile_" and "_Mean"
shorten_labels <- function(x) {
  gsub("datafile_", "", gsub("_Mean", "", x))
}
# Apply the function to row and column names
rownames(dPNRV_US.corr$r) <- shorten_labels(rownames(dPNRV_US.corr$r))
colnames(dPNRV_US.corr$r) <- shorten_labels(colnames(dPNRV_US.corr$r))
# Create a combined matrix with r and p values
combined_results <- matrix("", nrow = nrow(dPNRV_US.corr$r), ncol = ncol(dPNRV_US.corr$r))
combined_results[,] <- paste("r:", round(dPNRV_US.corr$r, 3), "\np:", formatted_p_values)
# Set row and column names
rownames(combined_results) <- gsub("US.|\\DDP", "", rownames(dPNRV_US.corr$r))
colnames(combined_results) <- gsub("US.|\\DDP", "",  colnames(dPNRV_US.corr$r))
# Output the combined results using pander
pander(combined_results)
```

Table continues below

	PI_Moral	PI_Conven
**PI_Moral**	r: 1 p: < 0.001	r: 0.835 p: < 0.001
**PI_Conven**	r: 0.835 p: < 0.001	r: 1 p: < 0.001

	PI__Moral	PI__Conven
**PI__Pruden**	r: 0.771 p: < 0.001	r: 0.675 p: < 0.001
**PI__Person**	r: 0.712 p: < 0.001	r: 0.708 p: < 0.001
**Parent__conflict**	r: -0.295 p: < 0.001	r: -0.237 p: 0.005
**Parent__antag**	r: -0.323 p: < 0.001	r: -0.218 p: 0.009
**Parent__affection**	r: 0.538 p: < 0.001	r: 0.527 p: < 0.001
**Parent__reassurance**	r: 0.654 p: < 0.001	r: 0.586 p: < 0.001
**Parent__satisfaction**	r: 0.586 p: < 0.001	r: 0.51 p: < 0.001

Table continues below

	PI__Pruden	PI__Person
**PI__Moral**	r: 0.771 p: < 0.001	r: 0.712 p: < 0.001
**PI__Conven**	r: 0.675 p: < 0.001	r: 0.708 p: < 0.001
**PI__Pruden**	r: 1 p: < 0.001	r: 0.651 p: < 0.001
**PI__Person**	r: 0.651 p: < 0.001	r: 1 p: < 0.001
**Parent__conflict**	r: -0.241 p: 0.004	r: -0.261 p: 0.002
**Parent__antag**	r: -0.318 p: < 0.001	r: -0.295 p: < 0.001
**Parent__affection**	r: 0.468 p: < 0.001	r: 0.499 p: < 0.001
**Parent__reassurance**	r: 0.567 p: < 0.001	r: 0.618 p: < 0.001
**Parent__satisfaction**	r: 0.512 p: < 0.001	r: 0.495 p: < 0.001

Table continues below

	Parent__conflict	Parent__antag
**PI__Moral**	r: -0.295 p: 0.003	r: -0.323 p: 0.001
**PI__Conven**	r: -0.237 p: 0.016	r: -0.218 p: 0.016
**PI__Pruden**	r: -0.241 p: 0.016	r: -0.318 p: 0.001
**PI__Person**	r: -0.261 p: 0.009	r: -0.295 p: 0.003
**Parent__conflict**	r: 1 p: < 0.001	r: 0.819 p: < 0.001
**Parent__antag**	r: 0.819 p: < 0.001	r: 1 p: < 0.001
**Parent__affection**	r: -0.232 p: 0.005	r: -0.279 p: 0.001
**Parent__reassurance**	r: -0.401 p: < 0.001	r: -0.417 p: < 0.001
**Parent__satisfaction**	r: -0.456 p: < 0.001	r: -0.467 p: < 0.001

Table continues below

	Parent__affection	Parent__reassurance
**PI__Moral**	r: 0.538 p: < 0.001	r: 0.654 p: < 0.001
**PI__Conven**	r: 0.527 p: < 0.001	r: 0.586 p: < 0.001
**PI__Pruden**	r: 0.468 p: < 0.001	r: 0.567 p: < 0.001
**PI__Person**	r: 0.499 p: < 0.001	r: 0.618 p: < 0.001
**Parent__conflict**	r: -0.232 p: 0.016	r: -0.401 p: < 0.001
**Parent__antag**	r: -0.279 p: 0.005	r: -0.417 p: < 0.001
**Parent__affection**	r: 1 p: < 0.001	r: 0.742 p: < 0.001
**Parent__reassurance**	r: 0.742 p: < 0.001	r: 1 p: < 0.001
**Parent__satisfaction**	r: 0.718 p: < 0.001	r: 0.74 p: < 0.001

	Parent_satisfaction
**PI_Moral**	r: 0.586 p: < 0.001
**PI_Conven**	r: 0.51 p: < 0.001
**PI_Pruden**	r: 0.512 p: < 0.001
**PI_Person**	r: 0.495 p: < 0.001
**Parent_conflict**	r: -0.456 p: < 0.001
**Parent_antag**	r: -0.467 p: < 0.001
**Parent_affection**	r: 0.718 p: < 0.001
**Parent_reassurance**	r: 0.74 p: < 0.001
**Parent_satisfaction**	r: 1 p: < 0.001

Create correlation and density plot for US sample

```
ggpairs(dPNRV_US,lower = list(continuous=my_fn))
```

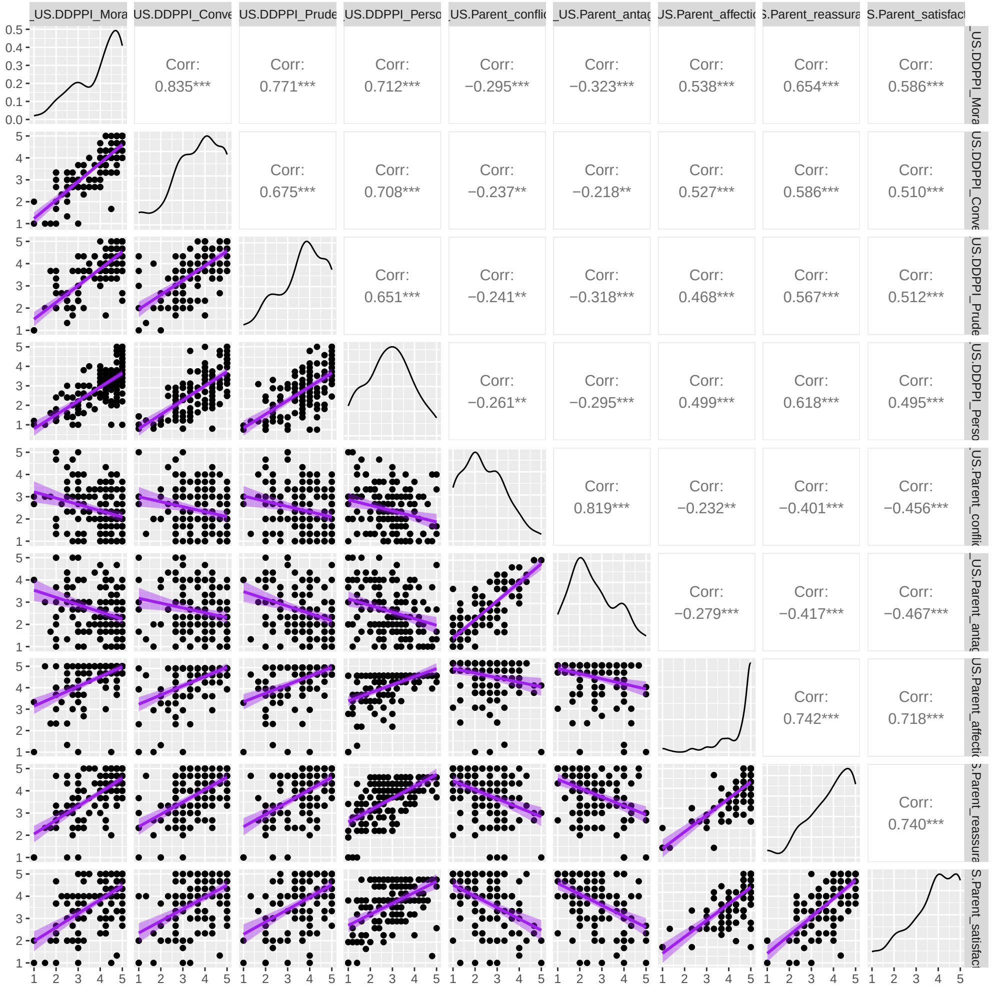

Correlations and Density Plots for DDPPI and NRI-SPV- US Sample

Domain-Differentiated Guilt Induction (DDPGI) and Domain-Differentiated Pride Induction (DDPPI)

This section showcases the code used to create the correlation and density plots for the factors of the DDPGI and DDPPI scales.

Create dataframe for HK sample

```
dPG_HK = data.frame(datafile_HK$DDPGI_Moral_Mean,datafile_HK$DDPGI_Conven_Mean,
                      datafile_HK$DDPGI_Pruden_Mean,datafile_HK$DDPGI_Person_Mean,
                      datafile_HK$DDPPI_Moral_Mean,datafile_HK$DDPPI_Conven_Mean,
                      datafile_HK$DDPPI_Pruden_Mean,datafile_HK$DDPPI_Person_Mean)
```

Estimated density plot in diagonal

```
my_fn <- function(data, mapping, ...){
  p <- ggplot(data =dPG_HK, mapping = mapping) +
    geom_point() + geom_smooth(method=lm, fill="blue", color="blue", ...)
  p }
```

Output r coefficients and P values

Parental Guilt Induction and Parental Pride Induction are labeled as 'GI' and 'PI' in the output for conciseness.

```
# Calculate correlation and p-values
dPG_HK.corr <- psych::corr.test(dPG_HK)
# Round p-values
rounded_p_values <- round(dPG_HK.corr$p, 3)
# Replace very small p-values with "< 0.0001"
formatted_p_values <- ifelse(rounded_p_values < 0.001, "< 0.001",
                              format(rounded_p_values, nsmall = 3,
                                     scientific = FALSE))
# Shorten variable labels by removing "datafile_" and "_Mean"
shorten_labels <- function(x) {
  gsub("datafile_", "", gsub("_Mean", "", x))
}
# Apply the function to row and column names
rownames(dPG_HK.corr$r) <- shorten_labels(rownames(dPG_HK.corr$r))
colnames(dPG_HK.corr$r) <- shorten_labels(colnames(dPG_HK.corr$r))
# Create a combined matrix with r and p values
combined_results <- matrix("", nrow = nrow(dPG_HK.corr$r), ncol = ncol(dPG_HK.corr$r))
combined_results[,] <- paste("r:", round(dPG_HK.corr$r, 3), "\np:", formatted_p_values)
# Set row and column names
rownames(combined_results) <- gsub("HK\\.DDP", "",rownames(dPG_HK.corr$r))
colnames(combined_results) <- gsub("HK\\.DDP", "",colnames(dPG_HK.corr$r))
# Output the combined results using pander
pander(combined_results)
```

Table continues below

	GI_Moral	GI_Conven
**GI_Moral**	r: 1 p: < 0.001	r: 0.608 p: < 0.001
**GI_Conven**	r: 0.608 p: < 0.001	r: 1 p: < 0.001
**GI_Pruden**	r: 0.804 p: < 0.001	r: 0.494 p: < 0.001
**GI_Person**	r: 0.379 p: < 0.001	r: 0.618 p: < 0.001
**PI_Moral**	r: 0.256 p: 0.004	r: 0.17 p: 0.059
**PI_Conven**	r: 0.281 p: 0.002	r: 0.208 p: 0.020
**PI_Pruden**	r: 0.233 p: 0.009	r: 0.187 p: 0.038
**PI_Person**	r: 0.089 p: 0.325	r: 0.043 p: 0.638

Table continues below

	GI_Pruden	GI_Person
**GI_Moral**	r: 0.804 p: < 0.001	r: 0.379 p: < 0.001
**GI_Conven**	r: 0.494 p: < 0.001	r: 0.618 p: < 0.001
**GI_Pruden**	r: 1 p: < 0.001	r: 0.294 p: 0.016
**GI_Person**	r: 0.294 p: 0.001	r: 1 p: < 0.001
**PI_Moral**	r: 0.243 p: 0.007	r: 0.025 p: 0.787
**PI_Conven**	r: 0.289 p: 0.001	r: 0.044 p: 0.627
**PI_Pruden**	r: 0.229 p: 0.011	r: -0.079 p: 0.383
**PI_Person**	r: 0.099 p: 0.275	r: 0.071 p: 0.435

Table continues below

	PI_Moral	PI_Conven
**GI_Moral**	r: 0.256 p: 0.057	r: 0.281 p: 0.024
**GI_Conven**	r: 0.17 p: 0.469	r: 0.208 p: 0.204
**GI_Pruden**	r: 0.243 p: 0.086	r: 0.289 p: 0.018
**GI_Person**	r: 0.025 p: 1.000	r: 0.044 p: 1.000
**PI_Moral**	r: 1 p: < 0.001	r: 0.804 p: < 0.001
**PI_Conven**	r: 0.804 p: < 0.001	r: 1 p: < 0.001
**PI_Pruden**	r: 0.745 p: < 0.001	r: 0.739 p: < 0.001
**PI_Person**	r: 0.751 p: < 0.001	r: 0.724 p: < 0.001

	PI_Pruden	PI_Person
**GI_Moral**	r: 0.233 p: 0.112	r: 0.089 p: 1.000
**GI_Conven**	r: 0.187 p: 0.338	r: 0.043 p: 1.000
**GI_Pruden**	r: 0.229 p: 0.117	r: 0.099 p: 1.000
**GI_Person**	r: -0.079 p: 1.000	r: 0.071 p: 1.000
**PI_Moral**	r: 0.745 p: < 0.001	r: 0.751 p: < 0.001
**PI_Conven**	r: 0.739 p: < 0.001	r: 0.724 p: < 0.001
**PI_Pruden**	r: 1 p: < 0.001	r: 0.661 p: < 0.001
**PI_Person**	r: 0.661 p: < 0.001	r: 1 p: < 0.001

Create correlation and density plot for HK sample

```
ggpairs(dPG_HK,lower = list(continuous=my_fn))
```

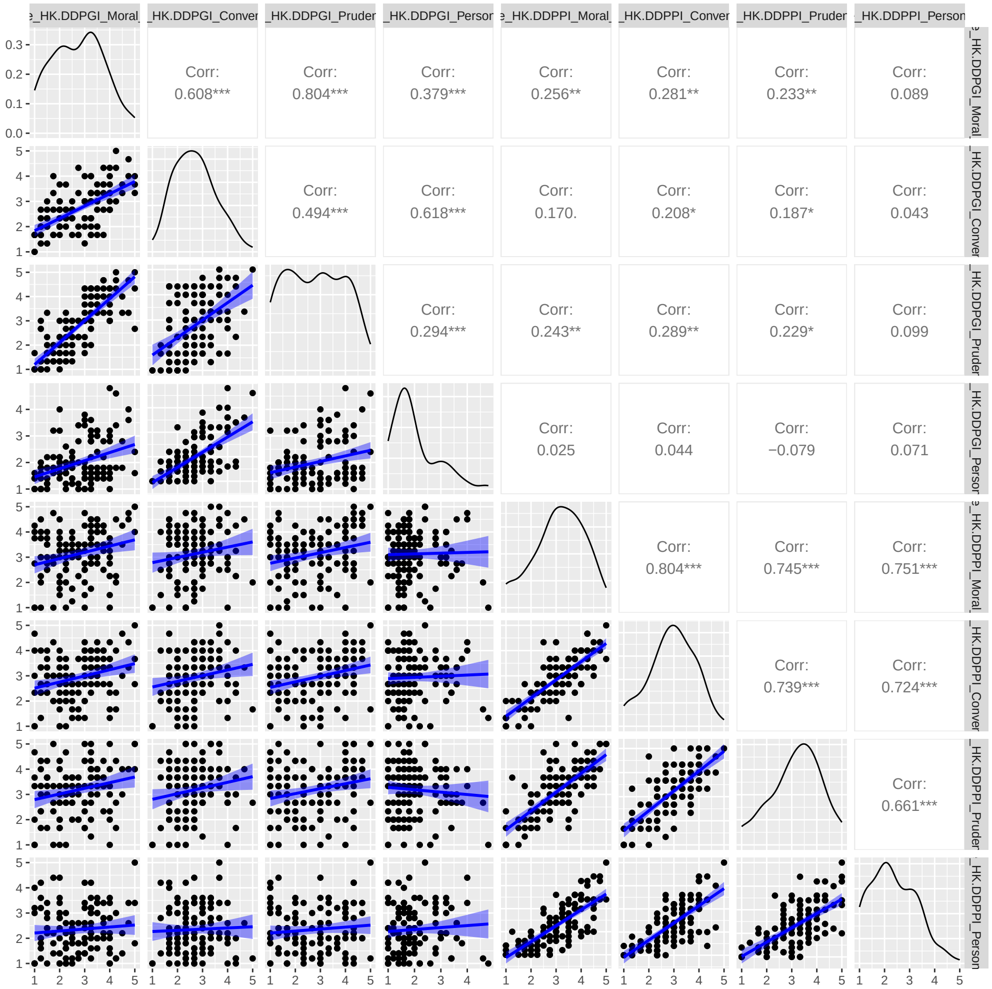

Correlations and Density Plots for DDPGI and DDPPI- Hong Kong Sample

Create dataframe for US sample

```
dPG_US = data.frame(datafile_US$DDPGI_Moral_Mean,datafile_US$DDPGI_Conven_Mean,
                      datafile_US$DDPGI_Pruden_Mean,datafile_US$DDPGI_Person_Mean,
                      datafile_US$DDPPI_Moral_Mean,datafile_US$DDPPI_Conven_Mean,
                      datafile_US$DDPPI_Pruden_Mean,datafile_US$DDPPI_Person_Mean)
```

Estimated density plot in diagonal

```
my_fn <- function(data, mapping, ...){
  p <- ggplot(data = dPG_US, mapping = mapping) +
    geom_point() + geom_smooth(method=lm, fill="purple", color="purple", ...)
  p }
```

Output r coefficients and P values

Parental Guilt Induction and Parental Pride Induction are labeled as 'GI' and 'PI' in the output for conciseness.

```
# Calculate correlation and p-values
dPG_US.corr <- psych::corr.test(dPG_US)
# Round p-values
rounded_p_values <- round(dPG_US.corr$p, 3)
# Replace very small p-values with "< 0.0001"
formatted_p_values <- ifelse(rounded_p_values < 0.001, "< 0.001",
                              format(rounded_p_values, nsmall = 3,
                                     scientific = FALSE))
# Shorten variable labels by removing "datafile_" and "_Mean"
shorten_labels <- function(x) {
  gsub("datafile_", "", gsub("_Mean", "", x))
}
# Apply the function to row and column names
rownames(dPG_US.corr$r) <- shorten_labels(rownames(dPG_US.corr$r))
colnames(dPG_US.corr$r) <- shorten_labels(colnames(dPG_US.corr$r))
# Create a combined matrix with r and p values
combined_results <- matrix("", nrow = nrow(dPG_US.corr$r), ncol = ncol(dPG_US.corr$r))
combined_results[,] <- paste("r:", round(dPG_US.corr$r, 3), "\np:", formatted_p_values)
# Set row and column names
rownames(combined_results) <- gsub("US\\.DDP", "", rownames(dPG_US.corr$r))
colnames(combined_results) <- gsub("US\\.DDP", "", colnames(dPG_US.corr$r))
# Output the combined results using pander
pander(combined_results)
```

Table continues below

	GI_Moral	GI_Conven
**GI_Moral**	r: 1 p: < 0.001	r: 0.671 p: < 0.001
**GI_Conven**	r: 0.671 p: < 0.001	r: 1 p: < 0.001

	GI_Moral	GI_Conven
**GI_Pruden**	r: 0.635 p: < 0.001	r: 0.61 p: < 0.001
**GI_Person**	r: 0.285 p: 0.001	r: 0.495 p: < 0.001
**PI_Moral**	r: 0.108 p: 0.202	r: -0.07 p: 0.410
**PI_Conven**	r: 0.158 p: 0.060	r: 0.024 p: 0.780
**PI_Pruden**	r: 0.099 p: 0.240	r: -0.082 p: 0.332
**PI_Person**	r: -0.027 p: 0.753	r: -0.182 p: 0.031

Table continues below

	GI_Pruden	GI_Person
**GI_Moral**	r: 0.635 p: < 0.001	r: 0.285 p: 0.008
**GI_Conven**	r: 0.61 p: < 0.001	r: 0.495 p: < 0.001
**GI_Pruden**	r: 1 p: < 0.001	r: 0.439 p: < 0.001
**GI_Person**	r: 0.439 p: < 0.001	r: 1 p: < 0.001
**PI_Moral**	r: -0.019 p: 0.826	r: -0.412 p: < 0.001
**PI_Conven**	r: 0.016 p: 0.854	r: -0.288 p: 0.001
**PI_Pruden**	r: 0.017 p: 0.840	r: -0.319 p: < 0.001
**PI_Person**	r: -0.121 p: 0.151	r: -0.411 p: < 0.001

Table continues below

	PI_Moral	PI_Conven
**GI_Moral**	r: 0.108 p: 1.000	r: 0.158 p: 0.665
**GI_Conven**	r: -0.07 p: 1.000	r: 0.024 p: 1.000
**GI_Pruden**	r: -0.019 p: 1.000	r: 0.016 p: 1.000
**GI_Person**	r: -0.412 p: < 0.001	r: -0.288 p: 0.007
**PI_Moral**	r: 1 p: < 0.001	r: 0.835 p: < 0.001
**PI_Conven**	r: 0.835 p: < 0.001	r: 1 p: < 0.001
**PI_Pruden**	r: 0.771 p: < 0.001	r: 0.675 p: < 0.001
**PI_Person**	r: 0.712 p: < 0.001	r: 0.708 p: < 0.001

	PI_Pruden	PI_Person
**GI_Moral**	r: 0.099 p: 1.000	r: -0.027 p: 1.000
**GI_Conven**	r: -0.082 p: 1.000	r: -0.182 p: 0.367
**GI_Pruden**	r: 0.017 p: 1.000	r: -0.121 p: 1.000
**GI_Person**	r: -0.319 p: 0.002	r: -0.411 p: < 0.001
**PI_Moral**	r: 0.771 p: < 0.001	r: 0.712 p: < 0.001
**PI_Conven**	r: 0.675 p: < 0.001	r: 0.708 p: < 0.001
**PI_Pruden**	r: 1 p: < 0.001	r: 0.651 p: < 0.001
**PI_Person**	r: 0.651 p: < 0.001	r: 1 p: < 0.001

Create correlation and density plot for US sample

```
ggpairs(dPG_US,lower = list(continuous=my_fn))
```

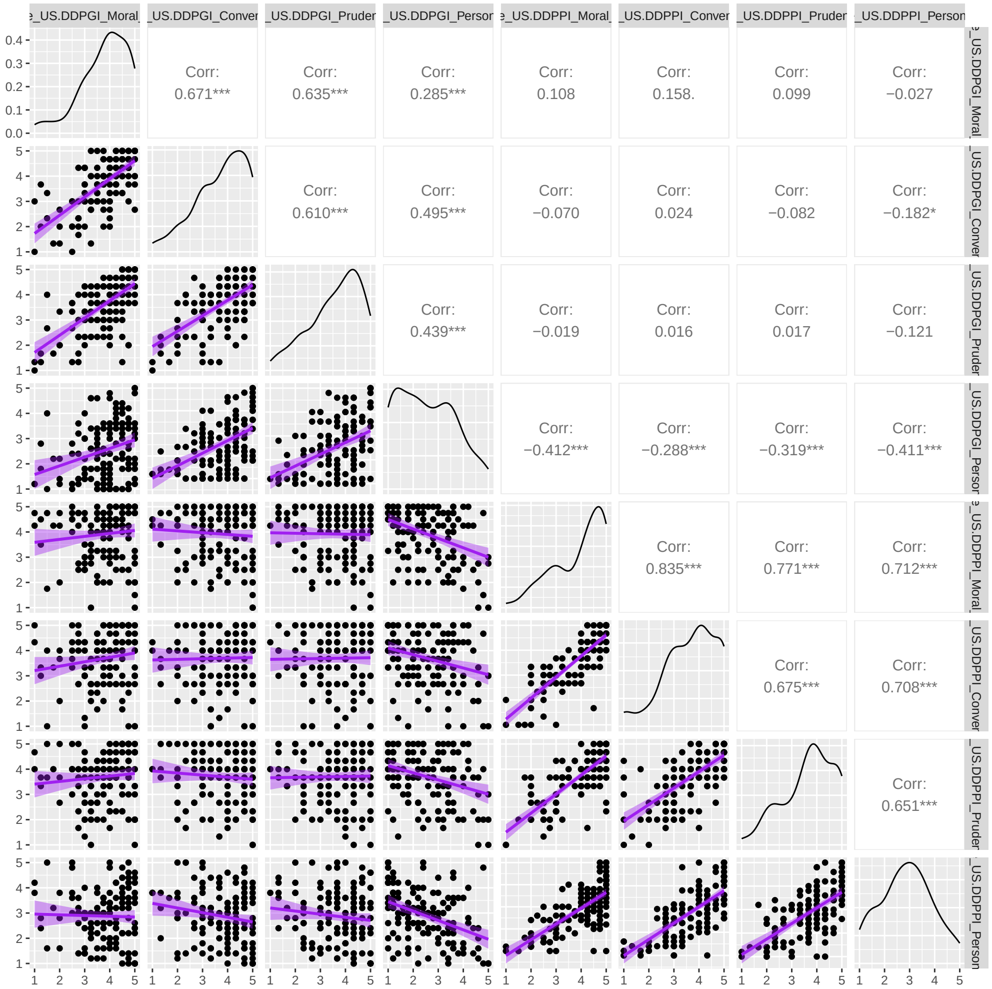

Correlations and Density Plots for DDPGI and DDPPI- US Sample
