# Supplementary material for: How parents leverage guilt and pride: A comparison of parental guilt and pride induction in Hong Kong and the United States
Source: J Res Adolesc. 2025 Dec 10;35(4):e70107. doi: 10.1111/jora.70107 (PMC12696220; doi:10.1111/jora.70107)
Supplement: Supplementary file 5 — Data S4: [file JORA-35-0-s001.pdf]

Measurement Invariance

Corresponding Author

2024-10-25

All measures used in the study were originally developed in English. The English measures were then translated into Chinese and subsequently back-translated into English by bilingual speakers. We utilized multi-group confirmatory factor analyses (MGCFA) to estimate reliabilities and measurement invariance using the semTools package (version 0.5-6; Jorgensen et al., 2016) in R 4.2.1 (R Core Team, 2022). Composite reliability coefficients were computed. All models were estimated using the maximum likelihood method.

Parental guilt and pride induction were measured using the Domain-Differentiated Guilt Induction scale (DDPGI; Rote et al., 2022) and the Domain-Differentiated Pride Induction scale (DDPPI), an adaptation of DDPGI. Each scale assesses 15 behaviors divided into four domains: moral (4 items), conventional (3 items), prudential (3 items), and personal (5 items).

Adolescent-parent relationship quality was mesured using 15 items from the Networks of Relationships Inventory, Social Provision Version (NRI-SPV; Furman & Buhrmester, 1985). The scale measured five features of adolescents' relationship quality with their parents, including two negative interaction features (i.e., conflict and antagonism) and three support features (i.e., affection, reassurance, and satisfaction), each feature consisting of three items.

Load packages

```
library(lavaan)
library(semTools)

options(max.print=999999)
```

Import data

```
datafile <- read.csv("D:\\R WM\\gipca data csv_with newly added data.csv")
```

Specify the model parameters for DDPGI

Modification indices suggested the presence of correlated residuals, and thus, residuals for four items across two paths were allowed to correlate.

```
model_DDPGI <- '
#latent factors
Moral =~ DDPGI_Moral_1 +DDPGI_Moral_2+ DDPGI_Moral_3 + DDPGI_Moral_4
Conventional =~ DDPGI_Conven_5+ DDPGI_Conven_6+ DDPGI_Conven_7
```

```
Prudential =~ DDPGI_Pruden_8+   DDPGI_Pruden_9+ DDPGI_Pruden_10
Personal =~ DDPGI_Person_11+    DDPGI_Person_12+    DDPGI_Person_13+
DDPGI_Person_14+    DDPGI_Person_15

#correlated residuals
DDPGI_Moral_4 ~~ DDPGI_Pruden_10
DDPGI_Conven_7 ~~ DDPGI_Person_15
'
```

Run a MGCFA for DDPGI: Configural Model

```
cfa.model <- cfa(model_DDPGI, data=datafile,
                 estimator = "ML", group = "Region")
```

Print summary of MGCFA for DDPGI: Configural Model

We used criteria of $CFI > .90$, $RMSEA < .08$, and $SRMR < .08$, which indicate a satisfactory model fit (Hu & Bentler, 1999; Kenny et al., 2015; MacCallum et al., 1996).

```
summary(cfa.model, fit.measures = TRUE, standardized = TRUE, ci = FALSE)
```

```
## lavaan 0.6.17 ended normally after 60 iterations
##
##   Estimator                                         ML
##   Optimization method                           NLMINB
##   Number of model parameters                       106
##
##   Number of observations per group:
##     HK                                             124
##     US                                             142
##
## Model Test User Model:
##
##   Test statistic                               240.549
##   Degrees of freedom                               164
##   P-value (Chi-square)                           0.000
##   Test statistic for each group:
##     HK                                         124.543
##     US                                         116.006
##
## Model Test Baseline Model:
##
##   Test statistic                              1888.802
##   Degrees of freedom                               210
##   P-value                                        0.000
##
## User Model versus Baseline Model:
##
##   Comparative Fit Index (CFI)                    0.954
##   Tucker-Lewis Index (TLI)                       0.942
```

```
##
## Loglikelihood and Information Criteria:
##
##   Loglikelihood user model (H0)              -5711.993
##   Loglikelihood unrestricted model (H1)      -5591.719
##
##   Akaike (AIC)                               11635.986
##   Bayesian (BIC)                             12015.837
##   Sample-size adjusted Bayesian (SABIC)      11679.757
##
## Root Mean Square Error of Approximation:
##
##   RMSEA                                          0.059
##   90 Percent confidence interval - lower         0.042
##   90 Percent confidence interval - upper         0.075
##   P-value H_0: RMSEA <= 0.050                    0.172
##   P-value H_0: RMSEA >= 0.080                    0.013
##
## Standardized Root Mean Square Residual:
##
##   SRMR                                           0.067
##
## Parameter Estimates:
##
##   Standard errors                             Standard
##   Information                                 Expected
##   Information saturated (h1) model          Structured
##
##
## Group 1 [HK]:
##
## Latent Variables:
##                    Estimate  Std.Err  z-value  P(>|z|)   Std.lv  Std.all
##   Moral =~
##     DDPGI_Moral_1     1.000                               0.736    0.617
##     DDPGI_Moral_2     0.948    0.161    5.873    0.000    0.697    0.632
##     DDPGI_Moral_3     1.569    0.216    7.264    0.000    1.154    0.859
##     DDPGI_Moral_4     1.515    0.231    6.560    0.000    1.115    0.733
##   Conventional =~
##     DDPGI_Conven_5    1.000                               0.749    0.667
##     DDPGI_Conven_6    1.021    0.153    6.678    0.000    0.765    0.679
##     DDPGI_Conven_7    0.600    0.135    4.454    0.000    0.449    0.432
##   Prudential =~
##     DDPGI_Pruden_8    1.000                               1.002    0.753
##     DDPGI_Pruden_9    0.866    0.120    7.210    0.000    0.868    0.672
##     DDPGI_Prudn_10    1.009    0.160    6.301    0.000    1.012    0.593
##   Personal =~
##     DDPGI_Persn_11    1.000                               0.901    0.772
##     DDPGI_Persn_12    0.912    0.121    7.528    0.000    0.822    0.713
##     DDPGI_Persn_13    0.604    0.095    6.370    0.000    0.544    0.606
##     DDPGI_Persn_14    0.791    0.110    7.186    0.000    0.712    0.681
##     DDPGI_Persn_15    0.842    0.118    7.112    0.000    0.758    0.669
##
## Covariances:
```

```
##                      Estimate  Std.Err  z-value  P(>|z|)   Std.lv  Std.all
##  .DDPGI_Moral_4 ~~
##   .DDPGI_Prudn_10     0.948    0.175    5.407    0.000    0.948    0.666
##  .DDPGI_Conven_7 ~~
##   .DDPGI_Persn_15     0.254    0.082    3.109    0.002    0.254    0.321
##   Moral ~~
##     Conventional      0.478    0.104    4.591    0.000    0.869    0.869
##     Prudential        0.720    0.142    5.060    0.000    0.976    0.976
##     Personal          0.319    0.088    3.629    0.000    0.482    0.482
##   Conventional ~~
##     Prudential        0.646    0.131    4.930    0.000    0.860    0.860
##     Personal          0.556    0.113    4.902    0.000    0.824    0.824
##   Prudential ~~
##     Personal          0.453    0.119    3.803    0.000    0.502    0.502
##
## Intercepts:
##                    Estimate  Std.Err  z-value  P(>|z|)   Std.lv  Std.all
##    .DDPGI_Moral_1     2.427    0.107   22.666    0.000    2.427    2.036
##    .DDPGI_Moral_2     2.839    0.099   28.667    0.000    2.839    2.574
##    .DDPGI_Moral_3     2.871    0.121   23.790    0.000    2.871    2.136
##    .DDPGI_Moral_4     2.855    0.137   20.892    0.000    2.855    1.876
##    .DDPGI_Conven_5    2.734    0.101   27.123    0.000    2.734    2.436
##    .DDPGI_Conven_6    2.710    0.101   26.769    0.000    2.710    2.404
##    .DDPGI_Conven_7    2.621    0.093   28.096    0.000    2.621    2.523
##    .DDPGI_Pruden_8    2.798    0.120   23.397    0.000    2.798    2.101
##    .DDPGI_Pruden_9    2.839    0.116   24.479    0.000    2.839    2.198
##    .DDPGI_Prudn_10    2.702    0.153   17.626    0.000    2.702    1.583
##    .DDPGI_Persn_11    2.040    0.105   19.473    0.000    2.040    1.749
##    .DDPGI_Persn_12    2.161    0.104   20.878    0.000    2.161    1.875
##    .DDPGI_Persn_13    1.653    0.081   20.491    0.000    1.653    1.840
##    .DDPGI_Persn_14    1.774    0.094   18.887    0.000    1.774    1.696
##    .DDPGI_Persn_15    2.331    0.102   22.887    0.000    2.331    2.055
##
## Variances:
##                    Estimate  Std.Err  z-value  P(>|z|)   Std.lv  Std.all
##    .DDPGI_Moral_1     0.881    0.121    7.262    0.000    0.881    0.620
##    .DDPGI_Moral_2     0.730    0.101    7.210    0.000    0.730    0.600
##    .DDPGI_Moral_3     0.474    0.095    4.976    0.000    0.474    0.262
##    .DDPGI_Moral_4     1.073    0.160    6.701    0.000    1.073    0.463
##    .DDPGI_Conven_5    0.699    0.107    6.556    0.000    0.699    0.555
##    .DDPGI_Conven_6    0.686    0.106    6.439    0.000    0.686    0.540
##    .DDPGI_Conven_7    0.877    0.115    7.607    0.000    0.877    0.813
##    .DDPGI_Pruden_8    0.769    0.128    6.005    0.000    0.769    0.434
##    .DDPGI_Pruden_9    0.914    0.134    6.795    0.000    0.914    0.548
##    .DDPGI_Prudn_10    1.890    0.262    7.200    0.000    1.890    0.649
##    .DDPGI_Persn_11    0.550    0.096    5.713    0.000    0.550    0.404
##    .DDPGI_Persn_12    0.653    0.103    6.362    0.000    0.653    0.492
##    .DDPGI_Persn_13    0.511    0.073    7.034    0.000    0.511    0.633
##    .DDPGI_Persn_14    0.587    0.089    6.615    0.000    0.587    0.537
##    .DDPGI_Persn_15    0.711    0.106    6.700    0.000    0.711    0.553
##     Moral             0.541    0.149    3.623    0.000    1.000    1.000
##     Conventional      0.561    0.146    3.851    0.000    1.000    1.000
##     Prudential        1.005    0.219    4.582    0.000    1.000    1.000
##     Personal          0.811    0.171    4.731    0.000    1.000    1.000
```

```
##
##
## Group 2 [US]:
##
## Latent Variables:
##                    Estimate  Std.Err  z-value  P(>|z|)   Std.lv  Std.all
##   Moral =~
##     DDPGI_Moral_1     1.000                               0.787    0.656
##     DDPGI_Moral_2     0.876    0.138    6.359    0.000    0.690    0.614
##     DDPGI_Moral_3     1.205    0.148    8.157    0.000    0.949    0.850
##     DDPGI_Moral_4     1.214    0.164    7.421    0.000    0.956    0.739
##   Conventional =~
##     DDPGI_Conven_5    1.000                               0.801    0.654
##     DDPGI_Conven_6    1.204    0.159    7.563    0.000    0.964    0.755
##     DDPGI_Conven_7    0.966    0.151    6.411    0.000    0.773    0.617
##   Prudential =~
##     DDPGI_Pruden_8    1.000                               0.928    0.756
##     DDPGI_Pruden_9    0.747    0.119    6.292    0.000    0.693    0.576
##     DDPGI_Prudn_10    0.976    0.140    6.995    0.000    0.905    0.640
##   Personal =~
##     DDPGI_Persn_11    1.000                               1.018    0.703
##     DDPGI_Persn_12    0.983    0.129    7.624    0.000    1.000    0.711
##     DDPGI_Persn_13    0.878    0.118    7.451    0.000    0.894    0.693
##     DDPGI_Persn_14    1.122    0.134    8.362    0.000    1.143    0.791
##     DDPGI_Persn_15    1.137    0.138    8.217    0.000    1.157    0.774
##
## Covariances:
##                      Estimate  Std.Err  z-value  P(>|z|)   Std.lv  Std.all
##  .DDPGI_Moral_4 ~~
##    .DDPGI_Prudn_10     0.212    0.096    2.213    0.027    0.212    0.224
##  .DDPGI_Conven_7 ~~
##    .DDPGI_Persn_15     0.075    0.092    0.812    0.417    0.075    0.080
##   Moral ~~
##     Conventional       0.557    0.111    5.035    0.000    0.884    0.884
##     Prudential         0.587    0.115    5.126    0.000    0.804    0.804
##     Personal           0.276    0.090    3.065    0.002    0.345    0.345
##   Conventional ~~
##     Prudential         0.675    0.126    5.363    0.000    0.908    0.908
##     Personal           0.504    0.115    4.387    0.000    0.618    0.618
##   Prudential ~~
##     Personal           0.547    0.125    4.392    0.000    0.580    0.580
##
## Intercepts:
##                    Estimate  Std.Err  z-value  P(>|z|)   Std.lv  Std.all
##    .DDPGI_Moral_1     3.725    0.101   37.011    0.000    3.725    3.106
##    .DDPGI_Moral_2     3.542    0.094   37.570    0.000    3.542    3.153
##    .DDPGI_Moral_3     4.021    0.094   42.927    0.000    4.021    3.602
##    .DDPGI_Moral_4     3.901    0.109   35.941    0.000    3.901    3.016
##    .DDPGI_Conven_5    3.859    0.103   37.532    0.000    3.859    3.150
##    .DDPGI_Conven_6    3.739    0.107   34.910    0.000    3.739    2.930
##    .DDPGI_Conven_7    3.641    0.105   34.628    0.000    3.641    2.906
##    .DDPGI_Pruden_8    3.873    0.103   37.622    0.000    3.873    3.157
##    .DDPGI_Pruden_9    3.535    0.101   35.033    0.000    3.535    2.940
##    .DDPGI_Prudn_10    3.465    0.119   29.181    0.000    3.465    2.449
```

```
##    .DDPGI_Persn_11    2.599    0.122   21.371    0.000    2.599    1.793
##    .DDPGI_Persn_12    2.676    0.118   22.665    0.000    2.676    1.902
##    .DDPGI_Persn_13    2.077    0.108   19.197    0.000    2.077    1.611
##    .DDPGI_Persn_14    2.585    0.121   21.314    0.000    2.585    1.789
##    .DDPGI_Persn_15    2.754    0.126   21.933    0.000    2.754    1.841
##
## Variances:
##                   Estimate  Std.Err  z-value  P(>|z|)   Std.lv  Std.all
##    .DDPGI_Moral_1    0.819    0.110    7.473    0.000    0.819    0.569
##    .DDPGI_Moral_2    0.786    0.103    7.669    0.000    0.786    0.623
##    .DDPGI_Moral_3    0.346    0.068    5.085    0.000    0.346    0.278
##    .DDPGI_Moral_4    0.760    0.110    6.890    0.000    0.760    0.454
##    .DDPGI_Conven_5   0.860    0.116    7.413    0.000    0.860    0.573
##    .DDPGI_Conven_6   0.700    0.110    6.365    0.000    0.700    0.430
##    .DDPGI_Conven_7   0.972    0.128    7.613    0.000    0.972    0.619
##    .DDPGI_Pruden_8   0.645    0.112    5.776    0.000    0.645    0.428
##    .DDPGI_Pruden_9   0.966    0.128    7.545    0.000    0.966    0.668
##    .DDPGI_Prudn_10   1.182    0.165    7.172    0.000    1.182    0.590
##    .DDPGI_Persn_11   1.063    0.149    7.149    0.000    1.063    0.506
##    .DDPGI_Persn_12   0.979    0.138    7.085    0.000    0.979    0.494
##    .DDPGI_Persn_13   0.863    0.120    7.214    0.000    0.863    0.519
##    .DDPGI_Persn_14   0.782    0.126    6.223    0.000    0.782    0.375
##    .DDPGI_Persn_15   0.899    0.139    6.458    0.000    0.899    0.402
##     Moral            0.620    0.149    4.154    0.000    1.000    1.000
##     Conventional     0.641    0.156    4.109    0.000    1.000    1.000
##     Prudential       0.860    0.181    4.762    0.000    1.000    1.000
##     Personal         1.036    0.229    4.529    0.000    1.000    1.000
```

Run a MGCFA for DDPGI: Metric Model

```
cfa.metric <- cfa(model_DDPGI, data=datafile,
                  estimator = "ML", group = "Region",

                  group.equal= "loadings", parameterization = "delta")
```

Print summary of MGCFA for DDPGI: Metric Model

We used criteria of $CFI > .90$, $RMSEA < .08$, and $SRMR < .08$, which indicate a satisfactory model fit (Hu & Bentler, 1999; Kenny et al., 2015; MacCallum et al., 1996).

```
summary(cfa.metric, fit.measures = TRUE, standardized = TRUE, ci = FALSE)
```

```
## lavaan 0.6.17 ended normally after 56 iterations
##
##   Estimator                                         ML
##   Optimization method                           NLMINB
##   Number of model parameters                       106
##   Number of equality constraints                    11
##
##   Number of observations per group:
```

```
##     HK                                             124
##     US                                             142
##
## Model Test User Model:
##
##   Test statistic                               253.084
##   Degrees of freedom                               175
##   P-value (Chi-square)                           0.000
##   Test statistic for each group:
##     HK                                         131.781
##     US                                         121.303
##
## Model Test Baseline Model:
##
##   Test statistic                              1888.802
##   Degrees of freedom                               210
##   P-value                                        0.000
##
## User Model versus Baseline Model:
##
##   Comparative Fit Index (CFI)                    0.953
##   Tucker-Lewis Index (TLI)                       0.944
##
## Loglikelihood and Information Criteria:
##
##   Loglikelihood user model (H0)              -5718.261
##   Loglikelihood unrestricted model (H1)      -5591.719
##
##   Akaike (AIC)                               11626.522
##   Bayesian (BIC)                             11966.954
##   Sample-size adjusted Bayesian (SABIC)      11665.750
##
## Root Mean Square Error of Approximation:
##
##   RMSEA                                          0.058
##   90 Percent confidence interval - lower         0.041
##   90 Percent confidence interval - upper         0.073
##   P-value H_0: RMSEA <= 0.050                    0.202
##   P-value H_0: RMSEA >= 0.080                    0.007
##
## Standardized Root Mean Square Residual:
##
##   SRMR                                           0.075
##
## Parameter Estimates:
##
##   Standard errors                             Standard
##   Information                                 Expected
##   Information saturated (h1) model          Structured
##
##
## Group 1 [HK]:
##
## Latent Variables:
```

```
##                    Estimate  Std.Err  z-value  P(>|z|)   Std.lv  Std.all
##   Moral =~
##     DDPGI_M           1.000                               0.810    0.656
##     DDPGI_M (.p2.)    0.910    0.105    8.666    0.000    0.737    0.657
##     DDPGI_M (.p3.)    1.367    0.126   10.851    0.000    1.108    0.841
##     DDPGI_M (.p4.)    1.352    0.136    9.940    0.000    1.095    0.723
##   Conventional =~
##     DDPGI_C           1.000                               0.679    0.625
##     DDPGI_C (.p6.)    1.127    0.112   10.085    0.000    0.765    0.678
##     DDPGI_C (.p7.)    0.814    0.102    7.957    0.000    0.553    0.508
##   Prudential =~
##     DDPGI_P           1.000                               1.024    0.763
##     DDPGI_P (.p9.)    0.808    0.084    9.582    0.000    0.827    0.651
##     DDPGI_P (.10.)    0.982    0.103    9.490    0.000    1.006    0.589
##   Personal =~
##     DDPGI_P           1.000                               0.805    0.722
##     DDPGI_P (.12.)    0.946    0.089   10.667    0.000    0.762    0.681
##     DDPGI_P (.13.)    0.750    0.076    9.908    0.000    0.604    0.650
##     DDPGI_P (.14.)    0.967    0.087   11.147    0.000    0.778    0.715
##     DDPGI_P (.15.)    1.009    0.091   11.067    0.000    0.812    0.698
##
## Covariances:
##                    Estimate  Std.Err  z-value  P(>|z|)   Std.lv  Std.all
##  .DDPGI_Moral_4 ~~
##    .DDPGI_Prudn_10    0.966    0.176    5.484    0.000    0.966    0.668
##  .DDPGI_Conven_7 ~~
##    .DDPGI_Persn_15    0.249    0.082    3.022    0.003    0.249    0.319
##   Moral ~~
##     Conventional      0.482    0.092    5.240    0.000    0.876    0.876
##     Prudential        0.811    0.139    5.847    0.000    0.977    0.977
##     Personal          0.318    0.081    3.912    0.000    0.487    0.487
##   Conventional ~~
##     Prudential        0.595    0.114    5.239    0.000    0.856    0.856
##     Personal          0.452    0.087    5.198    0.000    0.827    0.827
##   Prudential ~~
##     Personal          0.411    0.105    3.900    0.000    0.499    0.499
##
## Intercepts:
##                    Estimate  Std.Err  z-value  P(>|z|)   Std.lv  Std.all
##    .DDPGI_Moral_1     2.427    0.111   21.896    0.000    2.427    1.966
##    .DDPGI_Moral_2     2.839    0.101   28.151    0.000    2.839    2.528
##    .DDPGI_Moral_3     2.871    0.118   24.285    0.000    2.871    2.181
##    .DDPGI_Moral_4     2.855    0.136   20.969    0.000    2.855    1.883
##    .DDPGI_Conven_5    2.734    0.098   28.006    0.000    2.734    2.515
##    .DDPGI_Conven_6    2.710    0.101   26.749    0.000    2.710    2.402
##    .DDPGI_Conven_7    2.621    0.098   26.849    0.000    2.621    2.411
##    .DDPGI_Pruden_8    2.798    0.121   23.205    0.000    2.798    2.084
##    .DDPGI_Pruden_9    2.839    0.114   24.888    0.000    2.839    2.235
##    .DDPGI_Prudn_10    2.702    0.153   17.624    0.000    2.702    1.583
##    .DDPGI_Persn_11    2.040    0.100   20.389    0.000    2.040    1.831
##    .DDPGI_Persn_12    2.161    0.100   21.511    0.000    2.161    1.932
##    .DDPGI_Persn_13    1.653    0.083   19.831    0.000    1.653    1.781
##    .DDPGI_Persn_14    1.774    0.098   18.160    0.000    1.774    1.631
##    .DDPGI_Persn_15    2.331    0.104   22.322    0.000    2.331    2.005
```

```
##
## Variances:
##                    Estimate  Std.Err  z-value  P(>|z|)   Std.lv  Std.all
##    .DDPGI_Moral_1     0.867    0.121    7.145    0.000    0.867    0.569
##    .DDPGI_Moral_2     0.717    0.100    7.141    0.000    0.717    0.569
##    .DDPGI_Moral_3     0.506    0.093    5.447    0.000    0.506    0.292
##    .DDPGI_Moral_4     1.098    0.161    6.824    0.000    1.098    0.478
##    .DDPGI_Conven_5    0.721    0.104    6.926    0.000    0.721    0.610
##    .DDPGI_Conven_6    0.687    0.106    6.459    0.000    0.687    0.540
##    .DDPGI_Conven_7    0.876    0.118    7.452    0.000    0.876    0.742
##    .DDPGI_Pruden_8    0.754    0.128    5.882    0.000    0.754    0.418
##    .DDPGI_Pruden_9    0.929    0.134    6.945    0.000    0.929    0.576
##    .DDPGI_Prudn_10    1.902    0.263    7.238    0.000    1.902    0.653
##    .DDPGI_Persn_11    0.594    0.093    6.393    0.000    0.594    0.478
##    .DDPGI_Persn_12    0.671    0.100    6.712    0.000    0.671    0.536
##    .DDPGI_Persn_13    0.497    0.072    6.886    0.000    0.497    0.577
##    .DDPGI_Persn_14    0.578    0.089    6.461    0.000    0.578    0.488
##    .DDPGI_Persn_15    0.693    0.105    6.598    0.000    0.693    0.512
##     Moral             0.657    0.139    4.721    0.000    1.000    1.000
##     Conventional      0.461    0.109    4.237    0.000    1.000    1.000
##     Prudential        1.049    0.206    5.086    0.000    1.000    1.000
##     Personal          0.648    0.124    5.239    0.000    1.000    1.000
##
##
## Group 2 [US]:
##
## Latent Variables:
##                    Estimate  Std.Err  z-value  P(>|z|)   Std.lv  Std.all
##   Moral =~
##     DDPGI_M           1.000                               0.715    0.615
##     DDPGI_M (.p2.)    0.910    0.105    8.666    0.000    0.651    0.590
##     DDPGI_M (.p3.)    1.367    0.126   10.851    0.000    0.978    0.861
##     DDPGI_M (.p4.)    1.352    0.136    9.940    0.000    0.967    0.743
##   Conventional =~
##     DDPGI_C           1.000                               0.855    0.679
##     DDPGI_C (.p6.)    1.127    0.112   10.085    0.000    0.963    0.755
##     DDPGI_C (.p7.)    0.814    0.102    7.957    0.000    0.696    0.572
##   Prudential =~
##     DDPGI_P           1.000                               0.909    0.746
##     DDPGI_P (.p9.)    0.808    0.084    9.582    0.000    0.734    0.600
##     DDPGI_P (.10.)    0.982    0.103    9.490    0.000    0.892    0.633
##   Personal =~
##     DDPGI_P           1.000                               1.116    0.739
##     DDPGI_P (.12.)    0.946    0.089   10.667    0.000    1.056    0.733
##     DDPGI_P (.13.)    0.750    0.076    9.908    0.000    0.837    0.666
##     DDPGI_P (.14.)    0.967    0.087   11.147    0.000    1.079    0.767
##     DDPGI_P (.15.)    1.009    0.091   11.067    0.000    1.125    0.761
##
## Covariances:
##                     Estimate  Std.Err  z-value  P(>|z|)   Std.lv  Std.all
##  .DDPGI_Moral_4 ~~
##    .DDPGI_Prudn_10     0.215    0.096    2.238    0.025    0.215    0.226
##  .DDPGI_Conven_7 ~~
##    .DDPGI_Persn_15     0.085    0.093    0.913    0.361    0.085    0.088
```

```
##   Moral ~~
##     Conventional       0.542    0.096    5.626    0.000    0.886    0.886
##     Prudential         0.524    0.095    5.498    0.000    0.806    0.806
##     Personal           0.278    0.086    3.209    0.001    0.348    0.348
##   Conventional ~~
##     Prudential         0.709    0.121    5.866    0.000    0.913    0.913
##     Personal           0.583    0.123    4.729    0.000    0.612    0.612
##   Prudential ~~
##     Personal           0.594    0.127    4.678    0.000    0.586    0.586
##
## Intercepts:
##                    Estimate  Std.Err  z-value  P(>|z|)   Std.lv  Std.all
##    .DDPGI_Moral_1     3.725    0.098   38.173    0.000    3.725    3.203
##    .DDPGI_Moral_2     3.542    0.093   38.236    0.000    3.542    3.209
##    .DDPGI_Moral_3     4.021    0.095   42.225    0.000    4.021    3.543
##    .DDPGI_Moral_4     3.901    0.109   35.723    0.000    3.901    2.998
##    .DDPGI_Conven_5    3.859    0.106   36.562    0.000    3.859    3.068
##    .DDPGI_Conven_6    3.739    0.107   34.928    0.000    3.739    2.931
##    .DDPGI_Conven_7    3.641    0.102   35.701    0.000    3.641    2.996
##    .DDPGI_Pruden_8    3.873    0.102   37.905    0.000    3.873    3.181
##    .DDPGI_Pruden_9    3.535    0.103   34.455    0.000    3.535    2.891
##    .DDPGI_Prudn_10    3.465    0.118   29.286    0.000    3.465    2.458
##    .DDPGI_Persn_11    2.599    0.127   20.500    0.000    2.599    1.720
##    .DDPGI_Persn_12    2.676    0.121   22.132    0.000    2.676    1.857
##    .DDPGI_Persn_13    2.077    0.105   19.702    0.000    2.077    1.653
##    .DDPGI_Persn_14    2.585    0.118   21.899    0.000    2.585    1.838
##    .DDPGI_Persn_15    2.754    0.124   22.193    0.000    2.754    1.862
##
## Variances:
##                    Estimate  Std.Err  z-value  P(>|z|)   Std.lv  Std.all
##    .DDPGI_Moral_1     0.841    0.109    7.722    0.000    0.841    0.622
##    .DDPGI_Moral_2     0.795    0.102    7.814    0.000    0.795    0.652
##    .DDPGI_Moral_3     0.332    0.067    4.929    0.000    0.332    0.258
##    .DDPGI_Moral_4     0.759    0.109    6.953    0.000    0.759    0.448
##    .DDPGI_Conven_5    0.852    0.117    7.255    0.000    0.852    0.538
##    .DDPGI_Conven_6    0.700    0.109    6.396    0.000    0.700    0.430
##    .DDPGI_Conven_7    0.993    0.127    7.819    0.000    0.993    0.672
##    .DDPGI_Pruden_8    0.657    0.108    6.074    0.000    0.657    0.443
##    .DDPGI_Pruden_9    0.956    0.128    7.495    0.000    0.956    0.640
##    .DDPGI_Prudn_10    1.191    0.163    7.294    0.000    1.191    0.599
##    .DDPGI_Persn_11    1.037    0.150    6.911    0.000    1.037    0.454
##    .DDPGI_Persn_12    0.961    0.138    6.956    0.000    0.961    0.463
##    .DDPGI_Persn_13    0.879    0.118    7.429    0.000    0.879    0.557
##    .DDPGI_Persn_14    0.815    0.123    6.610    0.000    0.815    0.412
##    .DDPGI_Persn_15    0.919    0.138    6.675    0.000    0.919    0.421
##     Moral             0.512    0.107    4.803    0.000    1.000    1.000
##     Conventional      0.730    0.152    4.790    0.000    1.000    1.000
##     Prudential        0.826    0.159    5.202    0.000    1.000    1.000
##     Personal          1.245    0.225    5.521    0.000    1.000    1.000
```

Compare configural and metric models

Metric invariance can be rejected if the change in $CFI \geq -.010$ supplemented with the change in $RMSEA \geq .015$ or with the change in $SRMR \geq .030$ (Chen, 2007).

```
Comparison1Like<-compareFit(cfa.model, cfa.metric)
summary(Comparison1Like)
```

```
## #################### Nested Model Comparison #########################
##
## Chi-Squared Difference Test
##
##             Df   AIC   BIC  Chisq Chisq diff    RMSEA Df diff Pr(>Chisq)
## cfa.model  164 11636 12016 240.55
## cfa.metric 175 11626 11967 253.08     12.536 0.032397      11     0.3248
##
## ####################### Model Fit Indices ###########################
##               chisq  df pvalue rmsea   cfi   tli  srmr        aic        bic
## cfa.model  240.549† 164   .000 .059  .954† .942  .067† 11635.986  12015.837
## cfa.metric 253.084  175   .000 .058† .953  .944† .075  11626.522† 11966.954†
##
## ################## Differences in Fit Indices #######################
##                         df  rmsea    cfi   tli  srmr    aic     bic
## cfa.metric - cfa.model 11 -0.001 -0.001 0.003 0.008 -9.464 -48.883
```

Run a MGCFA for DDPGI: Scalar Model

```
cfa.scalar <- cfa(model_DDPGI, data=datafile,
                  estimator = "ML", group = "Region",

                  group.equal = c("loadings", "intercepts"))
```

Print summary of MGCFA for DDPGI: Scalar Model

We used criteria of $CFI > .90$, $RMSEA < .08$, and $SRMR < .08$, which indicate a satisfactory model fit (Hu & Bentler, 1999; Kenny et al., 2015; MacCallum et al., 1996).

```
summary(cfa.scalar , fit.measures = TRUE, standardized = TRUE, ci = FALSE)
```

```
## lavaan 0.6.17 ended normally after 72 iterations
##
##   Estimator                                         ML
##   Optimization method                           NLMINB
##   Number of model parameters                       110
##   Number of equality constraints                    26
##
##   Number of observations per group:
##     HK                                             124
##     US                                             142
##
```

```
## Model Test User Model:
##
##   Test statistic                               277.191
##   Degrees of freedom                               186
##   P-value (Chi-square)                           0.000
##   Test statistic for each group:
##     HK                                         147.666
##     US                                         129.525
##
## Model Test Baseline Model:
##
##   Test statistic                              1888.802
##   Degrees of freedom                               210
##   P-value                                        0.000
##
## User Model versus Baseline Model:
##
##   Comparative Fit Index (CFI)                    0.946
##   Tucker-Lewis Index (TLI)                       0.939
##
## Loglikelihood and Information Criteria:
##
##   Loglikelihood user model (H0)              -5730.314
##   Loglikelihood unrestricted model (H1)      -5591.719
##
##   Akaike (AIC)                               11628.629
##   Bayesian (BIC)                             11929.643
##   Sample-size adjusted Bayesian (SABIC)      11663.315
##
## Root Mean Square Error of Approximation:
##
##   RMSEA                                          0.061
##   90 Percent confidence interval - lower         0.045
##   90 Percent confidence interval - upper         0.075
##   P-value H_0: RMSEA <= 0.050                    0.121
##   P-value H_0: RMSEA >= 0.080                    0.013
##
## Standardized Root Mean Square Residual:
##
##   SRMR                                           0.080
##
## Parameter Estimates:
##
##   Standard errors                             Standard
##   Information                                 Expected
##   Information saturated (h1) model          Structured
##
##
## Group 1 [HK]:
##
## Latent Variables:
##                    Estimate  Std.Err  z-value  P(>|z|)   Std.lv  Std.all
##   Moral =~
##     DDPGI_M           1.000                               0.908    0.692
```

```
##     DDPGI_M (.p2.)    0.777    0.074   10.527    0.000    0.705    0.639
##     DDPGI_M (.p3.)    1.187    0.085   13.903    0.000    1.077    0.828
##     DDPGI_M (.p4.)    1.157    0.094   12.333    0.000    1.050    0.703
##   Conventional =~
##     DDPGI_C           1.000                                0.687    0.629
##     DDPGI_C (.p6.)    1.043    0.084   12.380    0.000    0.716    0.649
##     DDPGI_C (.p7.)    0.857    0.082   10.475    0.000    0.589    0.530
##   Prudential =~
##     DDPGI_P           1.000                                1.046    0.772
##     DDPGI_P (.p9.)    0.767    0.071   10.844    0.000    0.802    0.638
##     DDPGI_P (.10.)    0.914    0.087   10.506    0.000    0.957    0.567
##   Personal =~
##     DDPGI_P           1.000                                0.801    0.720
##     DDPGI_P (.12.)    0.946    0.084   11.209    0.000    0.757    0.679
##     DDPGI_P (.13.)    0.751    0.072   10.409    0.000    0.601    0.648
##     DDPGI_P (.14.)    1.013    0.085   11.937    0.000    0.811    0.728
##     DDPGI_P (.15.)    0.984    0.086   11.433    0.000    0.788    0.684
##
## Covariances:
##                     Estimate  Std.Err  z-value  P(>|z|)   Std.lv  Std.all
##  .DDPGI_Moral_4 ~~
##    .DDPGI_Prudn_10     0.994    0.178    5.581    0.000    0.994    0.673
##  .DDPGI_Conven_7 ~~
##    .DDPGI_Persn_15     0.249    0.083    2.990    0.003    0.249    0.314
##   Moral ~~
##     Conventional       0.554    0.099    5.570    0.000    0.888    0.888
##     Prudential         0.929    0.151    6.155    0.000    0.977    0.977
##     Personal           0.360    0.089    4.040    0.000    0.495    0.495
##   Conventional ~~
##     Prudential         0.619    0.115    5.398    0.000    0.862    0.862
##     Personal           0.457    0.086    5.314    0.000    0.831    0.831
##   Prudential ~~
##     Personal           0.424    0.107    3.965    0.000    0.506    0.506
##
## Intercepts:
##                   Estimate  Std.Err  z-value  P(>|z|)   Std.lv  Std.all
##    .DDPGI_M (.43.)    2.585    0.107   24.085    0.000    2.585    1.971
##    .DDPGI_M (.44.)    2.797    0.089   31.345    0.000    2.797    2.536
##    .DDPGI_M (.45.)    2.838    0.112   25.262    0.000    2.838    2.181
##    .DDPGI_M (.46.)    2.769    0.120   23.097    0.000    2.769    1.854
##    .DDPGI_C (.47.)    2.751    0.092   29.975    0.000    2.751    2.519
##    .DDPGI_C (.48.)    2.653    0.093   28.415    0.000    2.653    2.402
##    .DDPGI_C (.49.)    2.655    0.089   29.667    0.000    2.655    2.391
##    .DDPGI_P (.50.)    2.842    0.117   24.352    0.000    2.842    2.095
##    .DDPGI_P (.51.)    2.802    0.102   27.452    0.000    2.802    2.227
##    .DDPGI_P (.52.)    2.593    0.128   20.296    0.000    2.593    1.536
##    .DDPGI_P (.53.)    2.029    0.093   21.827    0.000    2.029    1.823
##    .DDPGI_P (.54.)    2.143    0.091   23.536    0.000    2.143    1.921
##    .DDPGI_P (.55.)    1.647    0.076   21.774    0.000    1.647    1.776
##    .DDPGI_P (.56.)    1.871    0.093   20.179    0.000    1.871    1.679
##    .DDPGI_P (.57.)    2.266    0.094   24.159    0.000    2.266    1.966
##
## Variances:
##                   Estimate  Std.Err  z-value  P(>|z|)   Std.lv  Std.all
```

```
##    .DDPGI_Moral_1     0.895    0.128    6.980    0.000    0.895    0.521
##    .DDPGI_Moral_2     0.719    0.100    7.205    0.000    0.719    0.591
##    .DDPGI_Moral_3     0.533    0.094    5.679    0.000    0.533    0.315
##    .DDPGI_Moral_4     1.127    0.163    6.927    0.000    1.127    0.505
##    .DDPGI_Conven_5    0.721    0.105    6.887    0.000    0.721    0.604
##    .DDPGI_Conven_6    0.706    0.105    6.729    0.000    0.706    0.579
##    .DDPGI_Conven_7    0.886    0.120    7.392    0.000    0.886    0.719
##    .DDPGI_Pruden_8    0.744    0.130    5.707    0.000    0.744    0.405
##    .DDPGI_Pruden_9    0.939    0.134    7.012    0.000    0.939    0.593
##    .DDPGI_Prudn_10    1.934    0.265    7.309    0.000    1.934    0.679
##    .DDPGI_Persn_11    0.597    0.093    6.416    0.000    0.597    0.482
##    .DDPGI_Persn_12    0.670    0.100    6.723    0.000    0.670    0.539
##    .DDPGI_Persn_13    0.498    0.072    6.895    0.000    0.498    0.580
##    .DDPGI_Persn_14    0.584    0.092    6.352    0.000    0.584    0.470
##    .DDPGI_Persn_15    0.708    0.106    6.698    0.000    0.708    0.533
##     Moral             0.824    0.155    5.332    0.000    1.000    1.000
##     Conventional      0.472    0.106    4.445    0.000    1.000    1.000
##     Prudential        1.095    0.209    5.242    0.000    1.000    1.000
##     Personal          0.641    0.121    5.319    0.000    1.000    1.000
##
##
## Group 2 [US]:
##
## Latent Variables:
##                    Estimate  Std.Err  z-value  P(>|z|)   Std.lv  Std.all
##   Moral =~
##     DDPGI_M           1.000                               0.812    0.664
##     DDPGI_M (.p2.)    0.777    0.074   10.527    0.000    0.630    0.575
##     DDPGI_M (.p3.)    1.187    0.085   13.903    0.000    0.963    0.856
##     DDPGI_M (.p4.)    1.157    0.094   12.333    0.000    0.939    0.732
##   Conventional =~
##     DDPGI_C           1.000                               0.877    0.692
##     DDPGI_C (.p6.)    1.043    0.084   12.380    0.000    0.915    0.732
##     DDPGI_C (.p7.)    0.857    0.082   10.475    0.000    0.752    0.607
##   Prudential =~
##     DDPGI_P           1.000                               0.935    0.758
##     DDPGI_P (.p9.)    0.767    0.071   10.844    0.000    0.717    0.591
##     DDPGI_P (.10.)    0.914    0.087   10.506    0.000    0.855    0.615
##   Personal =~
##     DDPGI_P           1.000                               1.109    0.737
##     DDPGI_P (.12.)    0.946    0.084   11.209    0.000    1.049    0.730
##     DDPGI_P (.13.)    0.751    0.072   10.409    0.000    0.832    0.664
##     DDPGI_P (.14.)    1.013    0.085   11.937    0.000    1.123    0.780
##     DDPGI_P (.15.)    0.984    0.086   11.433    0.000    1.091    0.746
##
## Covariances:
##                     Estimate  Std.Err  z-value  P(>|z|)   Std.lv  Std.all
##  .DDPGI_Moral_4 ~~
##    .DDPGI_Prudn_10     0.218    0.096    2.284    0.022    0.218    0.228
##  .DDPGI_Conven_7 ~~
##    .DDPGI_Persn_15     0.082    0.093    0.887    0.375    0.082    0.086
##   Moral ~~
##     Conventional       0.630    0.104    6.040    0.000    0.884    0.884
##     Prudential         0.610    0.106    5.775    0.000    0.803    0.803
```

```
##     Personal          0.312    0.096    3.242    0.001    0.346    0.346
##   Conventional ~~
##     Prudential        0.738    0.122    6.050    0.000    0.900    0.900
##     Personal          0.597    0.123    4.844    0.000    0.614    0.614
##   Prudential ~~
##     Personal          0.608    0.129    4.724    0.000    0.586    0.586
##
## Intercepts:
##                    Estimate  Std.Err  z-value  P(>|z|)   Std.lv  Std.all
##    .DDPGI_M (.43.)    2.585    0.107   24.085    0.000    2.585    2.116
##    .DDPGI_M (.44.)    2.797    0.089   31.345    0.000    2.797    2.552
##    .DDPGI_M (.45.)    2.838    0.112   25.262    0.000    2.838    2.523
##    .DDPGI_M (.46.)    2.769    0.120   23.097    0.000    2.769    2.158
##    .DDPGI_C (.47.)    2.751    0.092   29.975    0.000    2.751    2.169
##    .DDPGI_C (.48.)    2.653    0.093   28.415    0.000    2.653    2.121
##    .DDPGI_C (.49.)    2.655    0.089   29.667    0.000    2.655    2.142
##    .DDPGI_P (.50.)    2.842    0.117   24.352    0.000    2.842    2.302
##    .DDPGI_P (.51.)    2.802    0.102   27.452    0.000    2.802    2.309
##    .DDPGI_P (.52.)    2.593    0.128   20.296    0.000    2.593    1.864
##    .DDPGI_P (.53.)    2.029    0.093   21.827    0.000    2.029    1.348
##    .DDPGI_P (.54.)    2.143    0.091   23.536    0.000    2.143    1.493
##    .DDPGI_P (.55.)    1.647    0.076   21.774    0.000    1.647    1.313
##    .DDPGI_P (.56.)    1.871    0.093   20.179    0.000    1.871    1.299
##    .DDPGI_P (.57.)    2.266    0.094   24.159    0.000    2.266    1.549
##     Moral             1.012    0.129    7.845    0.000    1.247    1.247
##     Cnvntnl           1.091    0.127    8.599    0.000    1.244    1.244
##     Prudntl           0.999    0.148    6.768    0.000    1.068    1.068
##     Personl           0.587    0.132    4.450    0.000    0.530    0.530
##
## Variances:
##                    Estimate  Std.Err  z-value  P(>|z|)   Std.lv  Std.all
##    .DDPGI_Moral_1     0.834    0.111    7.516    0.000    0.834    0.559
##    .DDPGI_Moral_2     0.804    0.102    7.865    0.000    0.804    0.669
##    .DDPGI_Moral_3     0.338    0.066    5.125    0.000    0.338    0.267
##    .DDPGI_Moral_4     0.764    0.108    7.070    0.000    0.764    0.464
##    .DDPGI_Conven_5    0.839    0.117    7.152    0.000    0.839    0.522
##    .DDPGI_Conven_6    0.727    0.108    6.729    0.000    0.727    0.465
##    .DDPGI_Conven_7    0.970    0.126    7.681    0.000    0.970    0.632
##    .DDPGI_Pruden_8    0.649    0.111    5.855    0.000    0.649    0.426
##    .DDPGI_Pruden_9    0.959    0.127    7.541    0.000    0.959    0.651
##    .DDPGI_Prudn_10    1.204    0.163    7.405    0.000    1.204    0.622
##    .DDPGI_Persn_11    1.037    0.150    6.935    0.000    1.037    0.458
##    .DDPGI_Persn_12    0.962    0.138    6.983    0.000    0.962    0.466
##    .DDPGI_Persn_13    0.880    0.118    7.444    0.000    0.880    0.559
##    .DDPGI_Persn_14    0.812    0.126    6.457    0.000    0.812    0.392
##    .DDPGI_Persn_15    0.950    0.139    6.841    0.000    0.950    0.444
##     Moral             0.659    0.120    5.506    0.000    1.000    1.000
##     Conventional      0.769    0.148    5.187    0.000    1.000    1.000
##     Prudential        0.875    0.163    5.369    0.000    1.000    1.000
##     Personal          1.229    0.218    5.627    0.000    1.000    1.000
```

Compare metric and scalar models

Scalar invariance can be rejected if the change in $CFI \geq -.010$ supplemented with the change in $RMSEA \geq .015$ or with the change in $SRMR \geq .010$ (Chen, 2007).

```
Comparison2Like<-compareFit(cfa.metric, cfa.scalar)
summary(Comparison2Like)
```

```
## ################### Nested Model Comparison #########################
##
## Chi-Squared Difference Test
##
##             Df   AIC   BIC  Chisq Chisq diff    RMSEA Df diff Pr(>Chisq)
## cfa.metric 175 11626 11967 253.08
## cfa.scalar 186 11629 11930 277.19     24.107 0.094653      11    0.01229 *
## ---
## Signif. codes:  0 '***' 0.001 '**' 0.01 '*' 0.05 '.' 0.1 ' ' 1
##
## ####################### Model Fit Indices ###########################
##               chisq  df pvalue rmsea   cfi   tli  srmr        aic        bic
## cfa.metric 253.084† 175   .000 .058† .953† .944† .075† 11626.522† 11966.954
## cfa.scalar 277.191  186   .000 .061  .946  .939  .080  11628.629  11929.643†
##
## ################## Differences in Fit Indices #######################
##                         df rmsea    cfi    tli  srmr   aic     bic
## cfa.scalar - cfa.metric 11 0.003 -0.008 -0.006 0.005 2.107 -37.311
```

Run a MGCFA for DDPGI: Residual Model

```
cfa.residual <- cfa(model_DDPGI, data=datafile,
                    estimator = "ML", group = "Region",

                    group.equal = c("loadings", "intercepts", "residuals"))
```

Print summary of MGCFA for DDPGI: Residual Model

We used criteria of $CFI > .90$, $RMSEA < .08$, and $SRMR < .08$, which indicate a satisfactory model fit (Hu & Bentler, 1999; Kenny et al., 2015; MacCallum et al., 1996).

```
summary(cfa.residual, fit.measures = TRUE, standardized = TRUE, ci = FALSE)
```

```
## lavaan 0.6.17 ended normally after 80 iterations
##
##   Estimator                                         ML
##   Optimization method                           NLMINB
##   Number of model parameters                       110
##   Number of equality constraints                    41
##
##   Number of observations per group:
##     HK                                             124
```

```
##     US                                             142
##
## Model Test User Model:
##
##   Test statistic                               313.352
##   Degrees of freedom                               201
##   P-value (Chi-square)                           0.000
##   Test statistic for each group:
##     HK                                         170.056
##     US                                         143.296
##
## Model Test Baseline Model:
##
##   Test statistic                              1888.802
##   Degrees of freedom                               210
##   P-value                                        0.000
##
## User Model versus Baseline Model:
##
##   Comparative Fit Index (CFI)                    0.933
##   Tucker-Lewis Index (TLI)                       0.930
##
## Loglikelihood and Information Criteria:
##
##   Loglikelihood user model (H0)              -5748.395
##   Loglikelihood unrestricted model (H1)      -5591.719
##
##   Akaike (AIC)                               11634.790
##   Bayesian (BIC)                             11882.051
##   Sample-size adjusted Bayesian (SABIC)      11663.282
##
## Root Mean Square Error of Approximation:
##
##   RMSEA                                          0.065
##   90 Percent confidence interval - lower         0.051
##   90 Percent confidence interval - upper         0.078
##   P-value H_0: RMSEA <= 0.050                    0.044
##   P-value H_0: RMSEA >= 0.080                    0.032
##
## Standardized Root Mean Square Residual:
##
##   SRMR                                           0.085
##
## Parameter Estimates:
##
##   Standard errors                             Standard
##   Information                                 Expected
##   Information saturated (h1) model          Structured
##
##
## Group 1 [HK]:
##
## Latent Variables:
##                    Estimate  Std.Err  z-value  P(>|z|)   Std.lv  Std.all
```

```
##   Moral =~
##     DDPGI_M           1.000                               0.916    0.699
##     DDPGI_M (.p2.)    0.781    0.075   10.375    0.000    0.715    0.631
##     DDPGI_M (.p3.)    1.209    0.087   13.834    0.000    1.107    0.864
##     DDPGI_M (.p4.)    1.177    0.095   12.341    0.000    1.079    0.754
##   Conventional =~
##     DDPGI_C           1.000                               0.677    0.609
##     DDPGI_C (.p6.)    1.042    0.084   12.414    0.000    0.706    0.639
##     DDPGI_C (.p7.)    0.862    0.082   10.547    0.000    0.584    0.517
##   Prudential =~
##     DDPGI_P           1.000                               1.057    0.782
##     DDPGI_P (.p9.)    0.775    0.071   10.851    0.000    0.819    0.644
##     DDPGI_P (.10.)    0.926    0.088   10.554    0.000    0.979    0.623
##   Personal =~
##     DDPGI_P           1.000                               0.756    0.636
##     DDPGI_P (.12.)    0.959    0.086   11.146    0.000    0.725    0.622
##     DDPGI_P (.13.)    0.787    0.075   10.553    0.000    0.595    0.581
##     DDPGI_P (.14.)    1.049    0.087   12.013    0.000    0.793    0.689
##     DDPGI_P (.15.)    1.013    0.089   11.446    0.000    0.766    0.641
##
## Covariances:
##                     Estimate  Std.Err  z-value  P(>|z|)   Std.lv  Std.all
##  .DDPGI_Moral_4 ~~
##    .DDPGI_Prudn_10     0.713    0.102    6.997    0.000    0.713    0.618
##  .DDPGI_Conven_7 ~~
##    .DDPGI_Persn_15     0.292    0.083    3.492    0.000    0.292    0.329
##   Moral ~~
##     Conventional       0.542    0.099    5.489    0.000    0.873    0.873
##     Prudential         0.934    0.151    6.167    0.000    0.964    0.964
##     Personal           0.339    0.086    3.933    0.000    0.489    0.489
##   Conventional ~~
##     Prudential         0.609    0.114    5.335    0.000    0.851    0.851
##     Personal           0.444    0.084    5.273    0.000    0.867    0.867
##   Prudential ~~
##     Personal           0.401    0.104    3.871    0.000    0.502    0.502
##
## Intercepts:
##                   Estimate  Std.Err  z-value  P(>|z|)   Std.lv  Std.all
##    .DDPGI_M (.43.)    2.589    0.108   24.058    0.000    2.589    1.975
##    .DDPGI_M (.44.)    2.800    0.091   30.858    0.000    2.800    2.471
##    .DDPGI_M (.45.)    2.843    0.112   25.328    0.000    2.843    2.218
##    .DDPGI_M (.46.)    2.782    0.119   23.341    0.000    2.782    1.945
##    .DDPGI_C (.47.)    2.752    0.093   29.714    0.000    2.752    2.475
##    .DDPGI_C (.48.)    2.653    0.093   28.505    0.000    2.653    2.403
##    .DDPGI_C (.49.)    2.651    0.090   29.429    0.000    2.651    2.348
##    .DDPGI_P (.50.)    2.844    0.117   24.358    0.000    2.844    2.102
##    .DDPGI_P (.51.)    2.801    0.103   27.134    0.000    2.801    2.203
##    .DDPGI_P (.52.)    2.610    0.126   20.784    0.000    2.610    1.661
##    .DDPGI_P (.53.)    2.032    0.094   21.677    0.000    2.032    1.709
##    .DDPGI_P (.54.)    2.142    0.091   23.494    0.000    2.142    1.839
##    .DDPGI_P (.55.)    1.638    0.079   20.870    0.000    1.638    1.600
##    .DDPGI_P (.56.)    1.885    0.093   20.198    0.000    1.885    1.637
##    .DDPGI_P (.57.)    2.253    0.094   23.918    0.000    2.253    1.886
##
```

```
## Variances:
##                    Estimate  Std.Err  z-value  P(>|z|)   Std.lv  Std.all
##    .DDPGI_M (.18.)    0.879    0.086   10.252    0.000    0.879    0.511
##    .DDPGI_M (.19.)    0.772    0.072   10.650    0.000    0.772    0.601
##    .DDPGI_M (.20.)    0.417    0.056    7.458    0.000    0.417    0.254
##    .DDPGI_M (.21.)    0.882    0.089    9.879    0.000    0.882    0.431
##    .DDPGI_C (.22.)    0.778    0.079    9.885    0.000    0.778    0.629
##    .DDPGI_C (.23.)    0.721    0.076    9.507    0.000    0.721    0.591
##    .DDPGI_C (.24.)    0.933    0.088   10.646    0.000    0.933    0.732
##    .DDPGI_P (.25.)    0.711    0.086    8.236    0.000    0.711    0.389
##    .DDPGI_P (.26.)    0.946    0.092   10.240    0.000    0.946    0.585
##    .DDPGI_P (.27.)    1.508    0.143   10.530    0.000    1.508    0.611
##    .DDPGI_P (.28.)    0.841    0.088    9.525    0.000    0.841    0.595
##    .DDPGI_P (.29.)    0.831    0.086    9.667    0.000    0.831    0.613
##    .DDPGI_P (.30.)    0.695    0.069   10.035    0.000    0.695    0.663
##    .DDPGI_P (.31.)    0.697    0.079    8.860    0.000    0.697    0.526
##    .DDPGI_P (.32.)    0.840    0.089    9.490    0.000    0.840    0.589
##     Moral             0.839    0.156    5.366    0.000    1.000    1.000
##     Cnvntnl           0.459    0.104    4.396    0.000    1.000    1.000
##     Prudntl           1.118    0.208    5.384    0.000    1.000    1.000
##     Personl           0.572    0.114    5.035    0.000    1.000    1.000
##
##
## Group 2 [US]:
##
## Latent Variables:
##                    Estimate  Std.Err  z-value  P(>|z|)   Std.lv  Std.all
##   Moral =~
##     DDPGI_M           1.000                               0.791    0.645
##     DDPGI_M (.p2.)    0.781    0.075   10.375    0.000    0.617    0.575
##     DDPGI_M (.p3.)    1.209    0.087   13.834    0.000    0.956    0.829
##     DDPGI_M (.p4.)    1.177    0.095   12.341    0.000    0.931    0.704
##   Conventional =~
##     DDPGI_C           1.000                               0.883    0.707
##     DDPGI_C (.p6.)    1.042    0.084   12.414    0.000    0.920    0.735
##     DDPGI_C (.p7.)    0.862    0.082   10.547    0.000    0.761    0.619
##   Prudential =~
##     DDPGI_P           1.000                               0.909    0.733
##     DDPGI_P (.p9.)    0.775    0.071   10.851    0.000    0.704    0.586
##     DDPGI_P (.10.)    0.926    0.088   10.554    0.000    0.842    0.566
##   Personal =~
##     DDPGI_P           1.000                               1.097    0.767
##     DDPGI_P (.12.)    0.959    0.086   11.146    0.000    1.052    0.756
##     DDPGI_P (.13.)    0.787    0.075   10.553    0.000    0.863    0.719
##     DDPGI_P (.14.)    1.049    0.087   12.013    0.000    1.151    0.809
##     DDPGI_P (.15.)    1.013    0.089   11.446    0.000    1.112    0.772
##
## Covariances:
##                     Estimate  Std.Err  z-value  P(>|z|)   Std.lv  Std.all
##  .DDPGI_Moral_4 ~~
##    .DDPGI_Prudn_10     0.319    0.108    2.961    0.003    0.319    0.277
##  .DDPGI_Conven_7 ~~
##    .DDPGI_Persn_15     0.064    0.085    0.752    0.452    0.064    0.072
##   Moral ~~
```

```
##     Conventional       0.622    0.103    6.057    0.000    0.891    0.891
##     Prudential         0.598    0.104    5.770    0.000    0.832    0.832
##     Personal           0.300    0.093    3.234    0.001    0.346    0.346
##   Conventional ~~
##     Prudential         0.729    0.121    6.044    0.000    0.909    0.909
##     Personal           0.582    0.120    4.844    0.000    0.601    0.601
##   Prudential ~~
##     Personal           0.587    0.125    4.708    0.000    0.589    0.589
##
## Intercepts:
##                    Estimate  Std.Err  z-value  P(>|z|)   Std.lv  Std.all
##    .DDPGI_M (.43.)    2.589    0.108   24.058    0.000    2.589    2.111
##    .DDPGI_M (.44.)    2.800    0.091   30.858    0.000    2.800    2.607
##    .DDPGI_M (.45.)    2.843    0.112   25.328    0.000    2.843    2.465
##    .DDPGI_M (.46.)    2.782    0.119   23.341    0.000    2.782    2.104
##    .DDPGI_C (.47.)    2.752    0.093   29.714    0.000    2.752    2.206
##    .DDPGI_C (.48.)    2.653    0.093   28.505    0.000    2.653    2.119
##    .DDPGI_C (.49.)    2.651    0.090   29.429    0.000    2.651    2.156
##    .DDPGI_P (.50.)    2.844    0.117   24.358    0.000    2.844    2.293
##    .DDPGI_P (.51.)    2.801    0.103   27.134    0.000    2.801    2.332
##    .DDPGI_P (.52.)    2.610    0.126   20.784    0.000    2.610    1.752
##    .DDPGI_P (.53.)    2.032    0.094   21.677    0.000    2.032    1.421
##    .DDPGI_P (.54.)    2.142    0.091   23.494    0.000    2.142    1.539
##    .DDPGI_P (.55.)    1.638    0.079   20.870    0.000    1.638    1.365
##    .DDPGI_P (.56.)    1.885    0.093   20.198    0.000    1.885    1.326
##    .DDPGI_P (.57.)    2.253    0.094   23.918    0.000    2.253    1.564
##     Moral             0.995    0.128    7.752    0.000    1.258    1.258
##     Cnvntnl           1.091    0.127    8.614    0.000    1.236    1.236
##     Prudntl           0.990    0.147    6.730    0.000    1.089    1.089
##     Personl           0.574    0.129    4.454    0.000    0.523    0.523
##
## Variances:
##                    Estimate  Std.Err  z-value  P(>|z|)   Std.lv  Std.all
##    .DDPGI_M (.18.)    0.879    0.086   10.252    0.000    0.879    0.584
##    .DDPGI_M (.19.)    0.772    0.072   10.650    0.000    0.772    0.669
##    .DDPGI_M (.20.)    0.417    0.056    7.458    0.000    0.417    0.314
##    .DDPGI_M (.21.)    0.882    0.089    9.879    0.000    0.882    0.504
##    .DDPGI_C (.22.)    0.778    0.079    9.885    0.000    0.778    0.500
##    .DDPGI_C (.23.)    0.721    0.076    9.507    0.000    0.721    0.460
##    .DDPGI_C (.24.)    0.933    0.088   10.646    0.000    0.933    0.617
##    .DDPGI_P (.25.)    0.711    0.086    8.236    0.000    0.711    0.463
##    .DDPGI_P (.26.)    0.946    0.092   10.240    0.000    0.946    0.656
##    .DDPGI_P (.27.)    1.508    0.143   10.530    0.000    1.508    0.680
##    .DDPGI_P (.28.)    0.841    0.088    9.525    0.000    0.841    0.411
##    .DDPGI_P (.29.)    0.831    0.086    9.667    0.000    0.831    0.429
##    .DDPGI_P (.30.)    0.695    0.069   10.035    0.000    0.695    0.483
##    .DDPGI_P (.31.)    0.697    0.079    8.860    0.000    0.697    0.345
##    .DDPGI_P (.32.)    0.840    0.089    9.490    0.000    0.840    0.405
##     Moral             0.625    0.115    5.422    0.000    1.000    1.000
##     Cnvntnl           0.779    0.147    5.312    0.000    1.000    1.000
##     Prudntl           0.827    0.158    5.227    0.000    1.000    1.000
##     Personl           1.204    0.211    5.701    0.000    1.000    1.000
```

Compare scalar and residual models

Residual invariance can be rejected if the change in $CFI \geq -.010$ supplemented with the change in $RMSEA \geq .015$ or with the change in $SRMR \geq .010$ (Chen, 2007).

```
Comparison3Like<-compareFit(cfa.scalar,cfa.residual)
summary(Comparison3Like)
```

```
## #################### Nested Model Comparison #########################
##
## Chi-Squared Difference Test
##
##               Df   AIC   BIC  Chisq Chisq diff   RMSEA Df diff Pr(>Chisq)
## cfa.scalar   186 11629 11930 277.19
## cfa.residual 201 11635 11882 313.35     36.161 0.10299      15   0.001676 **
## ---
## Signif. codes:  0 '***' 0.001 '**' 0.01 '*' 0.05 '.' 0.1 ' ' 1
##
## ####################### Model Fit Indices ###########################
##                 chisq  df pvalue rmsea   cfi   tli  srmr        aic        bic
## cfa.scalar   277.191† 186   .000 .061† .946† .939† .080† 11628.629† 11929.643
## cfa.residual 313.352  201   .000 .065  .933  .930  .085  11634.790  11882.051†
##
## ################## Differences in Fit Indices #######################
##                             df rmsea    cfi    tli  srmr   aic     bic
## cfa.residual - cfa.scalar 15 0.004 -0.013 -0.009 0.005 6.161 -47.591
```

Compute composite reliability for DDPGI

```
compRelSEM(cfa.scalar,return.total = TRUE)
```

```
##   group Moral Conventional Prudential Personal total
## 1    HK 0.833        0.651      0.658     0.83 0.932
## 2    US 0.797        0.710      0.688     0.85 0.905
```

Specify the model parameters for DDPPI

Modification indices suggested the presence of correlated residuals, and thus, residuals for four items across two paths were allowed to correlate.

```
model_DDPPI <- '
#latent factors
Moral =~ DDPPI_Moral_1 +DDPPI_Moral_2+ DDPPI_Moral_3 + DDPPI_Moral_4
Conventional =~ DDPPI_Conven_5+ DDPPI_Conven_6+ DDPPI_Conven_7
Prudential =~ DDPPI_Pruden_8+   DDPPI_Pruden_9+ DDPPI_Pruden_10
Personal =~ DDPPI_Person_11+    DDPPI_Person_12+    DDPPI_Person_13+
DDPPI_Person_14+    DDPPI_Person_15

#correlated residuals
DDPPI_Moral_4 ~~ DDPPI_Pruden_10
```

```
DDPPI_Conven_7 ~~ DDPPI_Person_15
'
```

Run a MGCFA for DDPPI: Configural Model

```
cfa.model <- cfa(model_DDPPI, data=datafile,
                 estimator = "ML", group = "Region")
```

Print summary of MGCFA for DDPPI: Configural Model

We used criteria of $CFI > .90$, $RMSEA < .08$, and $SRMR < .08$, which indicate a satisfactory model fit (Hu & Bentler, 1999; Kenny et al., 2015; MacCallum et al., 1996).

```
summary(cfa.model, fit.measures = TRUE, standardized = TRUE, ci = FALSE)
```

```
## lavaan 0.6.17 ended normally after 60 iterations
##
##   Estimator                                         ML
##   Optimization method                           NLMINB
##   Number of model parameters                       106
##
##   Number of observations per group:
##     HK                                             124
##     US                                             142
##
## Model Test User Model:
##
##   Test statistic                               243.310
##   Degrees of freedom                               164
##   P-value (Chi-square)                           0.000
##   Test statistic for each group:
##     HK                                         136.044
##     US                                         107.266
##
## Model Test Baseline Model:
##
##   Test statistic                              2567.202
##   Degrees of freedom                               210
##   P-value                                        0.000
##
## User Model versus Baseline Model:
##
##   Comparative Fit Index (CFI)                    0.966
##   Tucker-Lewis Index (TLI)                       0.957
##
## Loglikelihood and Information Criteria:
##
##   Loglikelihood user model (H0)              -5215.381
##   Loglikelihood unrestricted model (H1)      -5093.726
##
```

```
##   Akaike (AIC)                               10642.762
##   Bayesian (BIC)                             11022.612
##   Sample-size adjusted Bayesian (SABIC)      10686.533
##
## Root Mean Square Error of Approximation:
##
##   RMSEA                                          0.060
##   90 Percent confidence interval - lower         0.044
##   90 Percent confidence interval - upper         0.076
##   P-value H_0: RMSEA <= 0.050                    0.145
##   P-value H_0: RMSEA >= 0.080                    0.017
##
## Standardized Root Mean Square Residual:
##
##   SRMR                                           0.042
##
## Parameter Estimates:
##
##   Standard errors                             Standard
##   Information                                 Expected
##   Information saturated (h1) model          Structured
##
##
## Group 1 [HK]:
##
## Latent Variables:
##                    Estimate  Std.Err  z-value  P(>|z|)   Std.lv  Std.all
##   Moral =~
##     DDPPI_Moral_1     1.000                               0.811    0.721
##     DDPPI_Moral_2     1.244    0.129    9.610    0.000    1.009    0.875
##     DDPPI_Moral_3     1.243    0.138    9.010    0.000    1.008    0.821
##     DDPPI_Moral_4     1.032    0.135    7.656    0.000    0.837    0.701
##   Conventional =~
##     DDPPI_Conven_5    1.000                               0.876    0.720
##     DDPPI_Conven_6    0.949    0.113    8.407    0.000    0.831    0.726
##     DDPPI_Conven_7    0.598    0.101    5.920    0.000    0.523    0.516
##   Prudential =~
##     DDPPI_Pruden_8    1.000                               0.719    0.639
##     DDPPI_Pruden_9    1.288    0.178    7.221    0.000    0.926    0.782
##     DDPPI_Prudn_10    1.208    0.201    6.014    0.000    0.869    0.622
##   Personal =~
##     DDPPI_Persn_11    1.000                               0.901    0.787
##     DDPPI_Persn_12    0.895    0.112    8.026    0.000    0.806    0.699
##     DDPPI_Persn_13    0.801    0.106    7.557    0.000    0.722    0.664
##     DDPPI_Persn_14    0.955    0.116    8.205    0.000    0.860    0.712
##     DDPPI_Persn_15    1.020    0.107    9.540    0.000    0.918    0.808
##
## Covariances:
##                     Estimate  Std.Err  z-value  P(>|z|)   Std.lv  Std.all
##  .DDPPI_Moral_4 ~~
##    .DDPPI_Prudn_10    -0.032    0.090   -0.358    0.720   -0.032   -0.035
##  .DDPPI_Conven_7 ~~
##    .DDPPI_Persn_15    -0.017    0.058   -0.296    0.767   -0.017   -0.030
##   Moral ~~
```

```
##     Conventional       0.746    0.131    5.713    0.000    1.051    1.051
##     Prudential         0.551    0.109    5.074    0.000    0.946    0.946
##     Personal           0.639    0.116    5.494    0.000    0.876    0.876
##   Conventional ~~
##     Prudential         0.655    0.125    5.252    0.000    1.041    1.041
##     Personal           0.730    0.130    5.614    0.000    0.926    0.926
##   Prudential ~~
##     Personal           0.540    0.109    4.964    0.000    0.834    0.834
##
## Intercepts:
##                    Estimate  Std.Err  z-value  P(>|z|)   Std.lv  Std.all
##    .DDPPI_Moral_1     3.040    0.101   30.107    0.000    3.040    2.704
##    .DDPPI_Moral_2     3.024    0.104   29.200    0.000    3.024    2.622
##    .DDPPI_Moral_3     3.185    0.110   28.906    0.000    3.185    2.596
##    .DDPPI_Moral_4     3.274    0.107   30.551    0.000    3.274    2.744
##    .DDPPI_Conven_5    3.218    0.109   29.485    0.000    3.218    2.648
##    .DDPPI_Conven_6    2.734    0.103   26.616    0.000    2.734    2.390
##    .DDPPI_Conven_7    2.855    0.091   31.368    0.000    2.855    2.817
##    .DDPPI_Pruden_8    3.629    0.101   35.921    0.000    3.629    3.226
##    .DDPPI_Pruden_9    2.895    0.106   27.244    0.000    2.895    2.447
##    .DDPPI_Prudn_10    3.032    0.125   24.172    0.000    3.032    2.171
##    .DDPPI_Persn_11    2.306    0.103   22.446    0.000    2.306    2.016
##    .DDPPI_Persn_12    2.508    0.104   24.212    0.000    2.508    2.174
##    .DDPPI_Persn_13    2.137    0.098   21.881    0.000    2.137    1.965
##    .DDPPI_Persn_14    2.484    0.108   22.896    0.000    2.484    2.056
##    .DDPPI_Persn_15    2.298    0.102   22.529    0.000    2.298    2.023
##
## Variances:
##                    Estimate  Std.Err  z-value  P(>|z|)   Std.lv  Std.all
##    .DDPPI_Moral_1     0.607    0.084    7.263    0.000    0.607    0.480
##    .DDPPI_Moral_2     0.312    0.053    5.854    0.000    0.312    0.235
##    .DDPPI_Moral_3     0.491    0.074    6.660    0.000    0.491    0.326
##    .DDPPI_Moral_4     0.724    0.099    7.322    0.000    0.724    0.508
##    .DDPPI_Conven_5    0.710    0.100    7.073    0.000    0.710    0.481
##    .DDPPI_Conven_6    0.618    0.088    7.022    0.000    0.618    0.472
##    .DDPPI_Conven_7    0.753    0.096    7.824    0.000    0.753    0.733
##    .DDPPI_Pruden_8    0.749    0.105    7.144    0.000    0.749    0.592
##    .DDPPI_Pruden_9    0.543    0.094    5.789    0.000    0.543    0.388
##    .DDPPI_Prudn_10    1.197    0.166    7.212    0.000    1.197    0.613
##    .DDPPI_Persn_11    0.498    0.078    6.377    0.000    0.498    0.381
##    .DDPPI_Persn_12    0.681    0.097    7.006    0.000    0.681    0.512
##    .DDPPI_Persn_13    0.662    0.092    7.160    0.000    0.662    0.560
##    .DDPPI_Persn_14    0.720    0.104    6.938    0.000    0.720    0.493
##    .DDPPI_Persn_15    0.447    0.073    6.125    0.000    0.447    0.346
##     Moral             0.658    0.145    4.550    0.000    1.000    1.000
##     Conventional      0.767    0.170    4.502    0.000    1.000    1.000
##     Prudential        0.517    0.137    3.777    0.000    1.000    1.000
##     Personal          0.811    0.160    5.054    0.000    1.000    1.000
##
##
## Group 2 [US]:
##
## Latent Variables:
##                    Estimate  Std.Err  z-value  P(>|z|)   Std.lv  Std.all
```

```
##   Moral =~
##     DDPPI_Moral_1     1.000                               1.008    0.842
##     DDPPI_Moral_2     1.058    0.081   13.113    0.000    1.066    0.861
##     DDPPI_Moral_3     1.058    0.076   13.974    0.000    1.066    0.893
##     DDPPI_Moral_4     0.748    0.077    9.658    0.000    0.753    0.706
##   Conventional =~
##     DDPPI_Conven_5    1.000                               0.810    0.734
##     DDPPI_Conven_6    1.271    0.126   10.098    0.000    1.029    0.845
##     DDPPI_Conven_7    1.209    0.135    8.939    0.000    0.980    0.754
##   Prudential =~
##     DDPPI_Pruden_8    1.000                               0.775    0.757
##     DDPPI_Pruden_9    1.156    0.139    8.304    0.000    0.896    0.703
##     DDPPI_Prudn_10    1.194    0.150    7.954    0.000    0.926    0.677
##   Personal =~
##     DDPPI_Persn_11    1.000                               1.027    0.779
##     DDPPI_Persn_12    0.955    0.102    9.372    0.000    0.981    0.767
##     DDPPI_Persn_13    0.942    0.105    9.006    0.000    0.967    0.740
##     DDPPI_Persn_14    0.889    0.102    8.753    0.000    0.912    0.722
##     DDPPI_Persn_15    0.825    0.104    7.895    0.000    0.847    0.660
##
## Covariances:
##                      Estimate  Std.Err  z-value  P(>|z|)   Std.lv  Std.all
##  .DDPPI_Moral_4 ~~
##    .DDPPI_Prudn_10    -0.063    0.070   -0.900    0.368   -0.063   -0.082
##  .DDPPI_Conven_7 ~~
##    .DDPPI_Persn_15     0.006    0.077    0.076    0.939    0.006    0.007
##   Moral ~~
##     Conventional       0.784    0.123    6.377    0.000    0.960    0.960
##     Prudential         0.740    0.115    6.412    0.000    0.947    0.947
##     Personal           0.828    0.138    6.024    0.000    0.801    0.801
##   Conventional ~~
##     Prudential         0.552    0.096    5.767    0.000    0.879    0.879
##     Personal           0.714    0.123    5.814    0.000    0.858    0.858
##   Prudential ~~
##     Personal           0.637    0.113    5.634    0.000    0.801    0.801
##
## Intercepts:
##                    Estimate  Std.Err  z-value  P(>|z|)   Std.lv  Std.all
##    .DDPPI_Moral_1     3.838    0.100   38.226    0.000    3.838    3.208
##    .DDPPI_Moral_2     3.732    0.104   35.910    0.000    3.732    3.014
##    .DDPPI_Moral_3     3.937    0.100   39.289    0.000    3.937    3.297
##    .DDPPI_Moral_4     4.169    0.090   46.562    0.000    4.169    3.907
##    .DDPPI_Conven_5    3.965    0.093   42.826    0.000    3.965    3.594
##    .DDPPI_Conven_6    3.718    0.102   36.372    0.000    3.718    3.052
##    .DDPPI_Conven_7    3.401    0.109   31.172    0.000    3.401    2.616
##    .DDPPI_Pruden_8    4.204    0.086   48.895    0.000    4.204    4.103
##    .DDPPI_Pruden_9    3.366    0.107   31.453    0.000    3.366    2.639
##    .DDPPI_Prudn_10    3.542    0.115   30.854    0.000    3.542    2.589
##    .DDPPI_Persn_11    3.035    0.111   27.434    0.000    3.035    2.302
##    .DDPPI_Persn_12    2.803    0.107   26.103    0.000    2.803    2.191
##    .DDPPI_Persn_13    2.599    0.110   23.714    0.000    2.599    1.990
##    .DDPPI_Persn_14    3.099    0.106   29.228    0.000    3.099    2.453
##    .DDPPI_Persn_15    2.852    0.108   26.479    0.000    2.852    2.222
##
```

```
## Variances:
##                    Estimate  Std.Err  z-value  P(>|z|)   Std.lv  Std.all
##    .DDPPI_Moral_1     0.416    0.058    7.136    0.000    0.416    0.291
##    .DDPPI_Moral_2     0.397    0.058    6.902    0.000    0.397    0.259
##    .DDPPI_Moral_3     0.288    0.046    6.289    0.000    0.288    0.202
##    .DDPPI_Moral_4     0.571    0.072    7.893    0.000    0.571    0.501
##    .DDPPI_Conven_5    0.561    0.074    7.538    0.000    0.561    0.461
##    .DDPPI_Conven_6    0.424    0.068    6.235    0.000    0.424    0.286
##    .DDPPI_Conven_7    0.731    0.099    7.410    0.000    0.731    0.432
##    .DDPPI_Pruden_8    0.449    0.067    6.714    0.000    0.449    0.428
##    .DDPPI_Pruden_9    0.823    0.113    7.259    0.000    0.823    0.506
##    .DDPPI_Prudn_10    1.014    0.137    7.422    0.000    1.014    0.542
##    .DDPPI_Persn_11    0.684    0.101    6.777    0.000    0.684    0.394
##    .DDPPI_Persn_12    0.675    0.098    6.902    0.000    0.675    0.412
##    .DDPPI_Persn_13    0.771    0.108    7.133    0.000    0.771    0.452
##    .DDPPI_Persn_14    0.764    0.105    7.267    0.000    0.764    0.479
##    .DDPPI_Persn_15    0.930    0.122    7.601    0.000    0.930    0.565
##     Moral             1.016    0.165    6.137    0.000    1.000    1.000
##     Conventional      0.656    0.132    4.956    0.000    1.000    1.000
##     Prudential        0.601    0.120    5.022    0.000    1.000    1.000
##     Personal          1.054    0.199    5.299    0.000    1.000    1.000
```

Run a MGCFA for DDPPI: Metric Model

```r
cfa.metric <- cfa(model_DDPPI, data=datafile,
                  estimator = "ML", group = "Region",

                  group.equal= "loadings", parameterization = "delta")
```

Print summary of MGCFA for DDPPI: Metric Model

We used criteria of $CFI > .90$, $RMSEA < .08$, and $SRMR < .08$, which indicate a satisfactory model fit (Hu & Bentler, 1999; Kenny et al., 2015; MacCallum et al., 1996).

```r
summary(cfa.metric, fit.measures = TRUE, standardized = TRUE, ci = FALSE)
```

```
## lavaan 0.6.17 ended normally after 54 iterations
##
##   Estimator                                         ML
##   Optimization method                           NLMINB
##   Number of model parameters                       106
##   Number of equality constraints                    11
##
##   Number of observations per group:
##     HK                                             124
##     US                                             142
##
## Model Test User Model:
##
##   Test statistic                               265.510
```

```
##   Degrees of freedom                               175
##   P-value (Chi-square)                           0.000
##   Test statistic for each group:
##     HK                                         149.599
##     US                                         115.911
##
## Model Test Baseline Model:
##
##   Test statistic                              2567.202
##   Degrees of freedom                               210
##   P-value                                        0.000
##
## User Model versus Baseline Model:
##
##   Comparative Fit Index (CFI)                    0.962
##   Tucker-Lewis Index (TLI)                       0.954
##
## Loglikelihood and Information Criteria:
##
##   Loglikelihood user model (H0)              -5226.481
##   Loglikelihood unrestricted model (H1)      -5093.726
##
##   Akaike (AIC)                               10642.961
##   Bayesian (BIC)                             10983.393
##   Sample-size adjusted Bayesian (SABIC)      10682.190
##
## Root Mean Square Error of Approximation:
##
##   RMSEA                                          0.062
##   90 Percent confidence interval - lower         0.047
##   90 Percent confidence interval - upper         0.077
##   P-value H_0: RMSEA <= 0.050                    0.094
##   P-value H_0: RMSEA >= 0.080                    0.024
##
## Standardized Root Mean Square Residual:
##
##   SRMR                                           0.065
##
## Parameter Estimates:
##
##   Standard errors                             Standard
##   Information                                 Expected
##   Information saturated (h1) model          Structured
##
##
## Group 1 [HK]:
##
## Latent Variables:
##                    Estimate  Std.Err  z-value  P(>|z|)   Std.lv  Std.all
##   Moral =~
##     DDPPI_M           1.000                               0.895    0.755
##     DDPPI_M (.p2.)    1.119    0.068   16.403    0.000    1.002    0.872
##     DDPPI_M (.p3.)    1.118    0.068   16.529    0.000    1.001    0.821
##     DDPPI_M (.p4.)    0.840    0.068   12.395    0.000    0.752    0.657
```

```
##   Conventional =~
##     DDPPI_C           1.000                               0.723    0.644
##     DDPPI_C (.p6.)    1.148    0.087   13.147    0.000    0.831    0.726
##     DDPPI_C (.p7.)    0.951    0.088   10.784    0.000    0.688    0.611
##   Prudential =~
##     DDPPI_P           1.000                               0.743    0.652
##     DDPPI_P (.p9.)    1.215    0.109   11.172    0.000    0.902    0.773
##     DDPPI_P (.10.)    1.195    0.120    9.955    0.000    0.887    0.630
##   Personal =~
##     DDPPI_P           1.000                               0.906    0.788
##     DDPPI_P (.12.)    0.933    0.076   12.361    0.000    0.846    0.719
##     DDPPI_P (.13.)    0.876    0.075   11.688    0.000    0.793    0.698
##     DDPPI_P (.14.)    0.922    0.077   11.997    0.000    0.835    0.702
##     DDPPI_P (.15.)    0.932    0.075   12.441    0.000    0.844    0.776
##
## Covariances:
##                    Estimate  Std.Err  z-value  P(>|z|)   Std.lv  Std.all
##  .DDPPI_Moral_4 ~~
##    .DDPPI_Prudn_10    -0.032    0.091   -0.354    0.723   -0.032   -0.034
##  .DDPPI_Conven_7 ~~
##    .DDPPI_Persn_15    -0.037    0.060   -0.613    0.540   -0.037   -0.060
##   Moral ~~
##     Conventional       0.683    0.106    6.424    0.000    1.055    1.055
##     Prudential         0.628    0.104    6.029    0.000    0.945    0.945
##     Personal           0.709    0.114    6.197    0.000    0.874    0.874
##   Conventional ~~
##     Prudential         0.559    0.094    5.926    0.000    1.041    1.041
##     Personal           0.620    0.103    6.030    0.000    0.947    0.947
##   Prudential ~~
##     Personal           0.564    0.101    5.612    0.000    0.839    0.839
##
## Intercepts:
##                  Estimate  Std.Err  z-value  P(>|z|)   Std.lv  Std.all
##    .DDPPI_Moral_1     3.040    0.107   28.541    0.000    3.040    2.563
##    .DDPPI_Moral_2     3.024    0.103   29.321    0.000    3.024    2.633
##    .DDPPI_Moral_3     3.185    0.109   29.114    0.000    3.185    2.615
##    .DDPPI_Moral_4     3.274    0.103   31.883    0.000    3.274    2.863
##    .DDPPI_Conven_5    3.218    0.101   31.917    0.000    3.218    2.866
##    .DDPPI_Conven_6    2.734    0.103   26.614    0.000    2.734    2.390
##    .DDPPI_Conven_7    2.855    0.101   28.257    0.000    2.855    2.538
##    .DDPPI_Pruden_8    3.629    0.102   35.451    0.000    3.629    3.184
##    .DDPPI_Pruden_9    2.895    0.105   27.599    0.000    2.895    2.478
##    .DDPPI_Prudn_10    3.032    0.126   23.992    0.000    3.032    2.155
##    .DDPPI_Persn_11    2.306    0.103   22.352    0.000    2.306    2.007
##    .DDPPI_Persn_12    2.508    0.106   23.741    0.000    2.508    2.132
##    .DDPPI_Persn_13    2.137    0.102   20.933    0.000    2.137    1.880
##    .DDPPI_Persn_14    2.484    0.107   23.254    0.000    2.484    2.088
##    .DDPPI_Persn_15    2.298    0.098   23.531    0.000    2.298    2.113
##
## Variances:
##                  Estimate  Std.Err  z-value  P(>|z|)   Std.lv  Std.all
##    .DDPPI_Moral_1     0.606    0.085    7.127    0.000    0.606    0.431
##    .DDPPI_Moral_2     0.316    0.053    5.915    0.000    0.316    0.239
##    .DDPPI_Moral_3     0.483    0.073    6.667    0.000    0.483    0.326
```

```
##    .DDPPI_Moral_4     0.743    0.100    7.446    0.000    0.743    0.568
##    .DDPPI_Conven_5    0.737    0.098    7.552    0.000    0.737    0.585
##    .DDPPI_Conven_6    0.618    0.088    7.033    0.000    0.618    0.473
##    .DDPPI_Conven_7    0.792    0.103    7.663    0.000    0.792    0.626
##    .DDPPI_Pruden_8    0.748    0.105    7.098    0.000    0.748    0.576
##    .DDPPI_Pruden_9    0.550    0.092    5.973    0.000    0.550    0.403
##    .DDPPI_Prudn_10    1.193    0.166    7.188    0.000    1.193    0.602
##    .DDPPI_Persn_11    0.500    0.078    6.431    0.000    0.500    0.378
##    .DDPPI_Persn_12    0.669    0.096    6.952    0.000    0.669    0.483
##    .DDPPI_Persn_13    0.663    0.094    7.055    0.000    0.663    0.513
##    .DDPPI_Persn_14    0.718    0.102    7.037    0.000    0.718    0.507
##    .DDPPI_Persn_15    0.471    0.072    6.524    0.000    0.471    0.398
##     Moral             0.801    0.136    5.896    0.000    1.000    1.000
##     Conventional      0.523    0.110    4.768    0.000    1.000    1.000
##     Prudential        0.552    0.117    4.717    0.000    1.000    1.000
##     Personal          0.821    0.143    5.748    0.000    1.000    1.000
##
##
## Group 2 [US]:
##
## Latent Variables:
##                    Estimate  Std.Err  z-value  P(>|z|)   Std.lv  Std.all
##   Moral =~
##     DDPPI_M           1.000                               0.958    0.827
##     DDPPI_M (.p2.)    1.119    0.068   16.403    0.000    1.072    0.862
##     DDPPI_M (.p3.)    1.118    0.068   16.529    0.000    1.071    0.894
##     DDPPI_M (.p4.)    0.840    0.068   12.395    0.000    0.805    0.730
##   Conventional =~
##     DDPPI_C           1.000                               0.896    0.768
##     DDPPI_C (.p6.)    1.148    0.087   13.147    0.000    1.029    0.845
##     DDPPI_C (.p7.)    0.951    0.088   10.784    0.000    0.852    0.697
##   Prudential =~
##     DDPPI_P           1.000                               0.761    0.749
##     DDPPI_P (.p9.)    1.215    0.109   11.172    0.000    0.924    0.715
##     DDPPI_P (.10.)    1.195    0.120    9.955    0.000    0.909    0.669
##   Personal =~
##     DDPPI_P           1.000                               1.017    0.775
##     DDPPI_P (.12.)    0.933    0.076   12.361    0.000    0.950    0.753
##     DDPPI_P (.13.)    0.876    0.075   11.688    0.000    0.891    0.707
##     DDPPI_P (.14.)    0.922    0.077   11.997    0.000    0.938    0.733
##     DDPPI_P (.15.)    0.932    0.075   12.441    0.000    0.948    0.704
##
## Covariances:
##                     Estimate  Std.Err  z-value  P(>|z|)   Std.lv  Std.all
##  .DDPPI_Moral_4 ~~
##    .DDPPI_Prudn_10    -0.064    0.070   -0.916    0.359   -0.064   -0.084
##  .DDPPI_Conven_7 ~~
##    .DDPPI_Persn_15    -0.002    0.078   -0.022    0.982   -0.002   -0.002
##   Moral ~~
##     Conventional       0.826    0.121    6.825    0.000    0.961    0.961
##     Prudential         0.692    0.104    6.668    0.000    0.949    0.949
##     Personal           0.784    0.124    6.326    0.000    0.804    0.804
##   Conventional ~~
##     Prudential         0.601    0.097    6.165    0.000    0.881    0.881
```

```
##     Personal           0.784    0.124    6.306    0.000    0.860    0.860
##   Prudential ~~
##     Personal           0.624    0.105    5.952    0.000    0.806    0.806
##
## Intercepts:
##                    Estimate  Std.Err  z-value  P(>|z|)   Std.lv  Std.all
##    .DDPPI_Moral_1     3.838    0.097   39.450    0.000    3.838    3.311
##    .DDPPI_Moral_2     3.732    0.104   35.770    0.000    3.732    3.002
##    .DDPPI_Moral_3     3.937    0.101   39.136    0.000    3.937    3.284
##    .DDPPI_Moral_4     4.169    0.093   45.035    0.000    4.169    3.779
##    .DDPPI_Conven_5    3.965    0.098   40.514    0.000    3.965    3.400
##    .DDPPI_Conven_6    3.718    0.102   36.374    0.000    3.718    3.052
##    .DDPPI_Conven_7    3.401    0.103   33.161    0.000    3.401    2.783
##    .DDPPI_Pruden_8    4.204    0.085   49.318    0.000    4.204    4.139
##    .DDPPI_Pruden_9    3.366    0.109   31.013    0.000    3.366    2.603
##    .DDPPI_Prudn_10    3.542    0.114   31.080    0.000    3.542    2.608
##    .DDPPI_Persn_11    3.035    0.110   27.539    0.000    3.035    2.311
##    .DDPPI_Persn_12    2.803    0.106   26.493    0.000    2.803    2.223
##    .DDPPI_Persn_13    2.599    0.106   24.556    0.000    2.599    2.061
##    .DDPPI_Persn_14    3.099    0.107   28.859    0.000    3.099    2.422
##    .DDPPI_Persn_15    2.852    0.113   25.257    0.000    2.852    2.119
##
## Variances:
##                    Estimate  Std.Err  z-value  P(>|z|)   Std.lv  Std.all
##    .DDPPI_Moral_1     0.426    0.058    7.310    0.000    0.426    0.317
##    .DDPPI_Moral_2     0.396    0.057    6.908    0.000    0.396    0.256
##    .DDPPI_Moral_3     0.289    0.046    6.302    0.000    0.289    0.201
##    .DDPPI_Moral_4     0.569    0.073    7.827    0.000    0.569    0.468
##    .DDPPI_Conven_5    0.557    0.076    7.306    0.000    0.557    0.410
##    .DDPPI_Conven_6    0.425    0.068    6.232    0.000    0.425    0.287
##    .DDPPI_Conven_7    0.768    0.099    7.730    0.000    0.768    0.514
##    .DDPPI_Pruden_8    0.453    0.066    6.847    0.000    0.453    0.439
##    .DDPPI_Pruden_9    0.819    0.114    7.201    0.000    0.819    0.489
##    .DDPPI_Prudn_10    1.019    0.136    7.499    0.000    1.019    0.552
##    .DDPPI_Persn_11    0.690    0.100    6.890    0.000    0.690    0.400
##    .DDPPI_Persn_12    0.688    0.097    7.080    0.000    0.688    0.433
##    .DDPPI_Persn_13    0.796    0.107    7.412    0.000    0.796    0.501
##    .DDPPI_Persn_14    0.758    0.105    7.239    0.000    0.758    0.463
##    .DDPPI_Persn_15    0.913    0.123    7.428    0.000    0.913    0.504
##     Moral             0.918    0.143    6.426    0.000    1.000    1.000
##     Conventional      0.803    0.141    5.692    0.000    1.000    1.000
##     Prudential        0.579    0.108    5.368    0.000    1.000    1.000
##     Personal          1.035    0.173    5.977    0.000    1.000    1.000
```

Compare configural and metric models

Metric invariance can be rejected if the change in $CFI \geq -.010$ supplemented with the change in $RMSEA \geq .015$ or with the change in $SRMR \geq .030$ (Chen, 2007).

```
Comparison1Like<-compareFit(cfa.model, cfa.metric)
summary(Comparison1Like)
```

```
## ################### Nested Model Comparison #########################
```

```
##
## Chi-Squared Difference Test
##
##              Df   AIC   BIC  Chisq Chisq diff    RMSEA Df diff Pr(>Chisq)
## cfa.model  164 10643 11023 243.31
## cfa.metric 175 10643 10983 265.51       22.2 0.087494      11    0.02287 *
## ---
## Signif. codes:  0 '***' 0.001 '**' 0.01 '*' 0.05 '.' 0.1 ' ' 1
##
## ####################### Model Fit Indices ###########################
##                 chisq  df pvalue rmsea   cfi   tli  srmr        aic        bic
## cfa.model  243.310† 164   .000 .060† .966† .957† .042† 10642.762† 11022.612
## cfa.metric 265.510  175   .000 .062  .962  .954  .065  10642.961  10983.393†
##
## ################## Differences in Fit Indices #######################
##                         df rmsea    cfi    tli  srmr aic     bic
## cfa.metric - cfa.model 11 0.002 -0.005 -0.003 0.023 0.2 -39.219
```

Run a MGCFA for DDPPI: Scalar Model

```r
cfa.scalar <- cfa(model_DDPPI, data=datafile,
                  estimator = "ML", group = "Region",

                  group.equal = c("loadings", "intercepts"))
```

Print summary of MGCFA for DDPPI: Scalar Model

We used criteria of $CFI > .90$, $RMSEA < .08$, and $SRMR < .08$, which indicate a satisfactory model fit (Hu & Bentler, 1999; Kenny et al., 2015; MacCallum et al., 1996).

```r
summary(cfa.scalar , fit.measures = TRUE, standardized = TRUE, ci = FALSE)
```

```
## lavaan 0.6.17 ended normally after 85 iterations
##
##   Estimator                                         ML
##   Optimization method                           NLMINB
##   Number of model parameters                       110
##   Number of equality constraints                    26
##
##   Number of observations per group:
##     HK                                             124
##     US                                             142
##
## Model Test User Model:
##
##   Test statistic                               288.826
##   Degrees of freedom                               186
##   P-value (Chi-square)                           0.000
##   Test statistic for each group:
##     HK                                         159.004
```

```
##     US                                         129.822
##
## Model Test Baseline Model:
##
##   Test statistic                              2567.202
##   Degrees of freedom                               210
##   P-value                                        0.000
##
## User Model versus Baseline Model:
##
##   Comparative Fit Index (CFI)                    0.956
##   Tucker-Lewis Index (TLI)                       0.951
##
## Loglikelihood and Information Criteria:
##
##   Loglikelihood user model (H0)              -5238.139
##   Loglikelihood unrestricted model (H1)      -5093.726
##
##   Akaike (AIC)                               10644.278
##   Bayesian (BIC)                             10945.292
##   Sample-size adjusted Bayesian (SABIC)      10678.964
##
## Root Mean Square Error of Approximation:
##
##   RMSEA                                          0.064
##   90 Percent confidence interval - lower         0.050
##   90 Percent confidence interval - upper         0.079
##   P-value H_0: RMSEA <= 0.050                    0.055
##   P-value H_0: RMSEA >= 0.080                    0.035
##
## Standardized Root Mean Square Residual:
##
##   SRMR                                           0.069
##
## Parameter Estimates:
##
##   Standard errors                             Standard
##   Information                                 Expected
##   Information saturated (h1) model          Structured
##
##
## Group 1 [HK]:
##
## Latent Variables:
##                    Estimate  Std.Err  z-value  P(>|z|)   Std.lv  Std.all
##   Moral =~
##     DDPPI_M           1.000                               0.908    0.759
##     DDPPI_M (.p2.)    1.082    0.061   17.625    0.000    0.983    0.865
##     DDPPI_M (.p3.)    1.092    0.061   17.809    0.000    0.992    0.818
##     DDPPI_M (.p4.)    0.881    0.064   13.838    0.000    0.800    0.678
##   Conventional =~
##     DDPPI_C           1.000                               0.725    0.645
##     DDPPI_C (.p6.)    1.176    0.081   14.548    0.000    0.852    0.733
##     DDPPI_C (.p7.)    0.905    0.079   11.472    0.000    0.656    0.594
```

```
##   Prudential =~
##     DDPPI_P            1.000                                0.762    0.661
##     DDPPI_P (.p9.)     1.164    0.099   11.778    0.000    0.887    0.764
##     DDPPI_P (.10.)     1.156    0.110   10.505    0.000    0.881    0.627
##   Personal =~
##     DDPPI_P            1.000                                0.929    0.796
##     DDPPI_P (.12.)     0.869    0.069   12.632    0.000    0.807    0.696
##     DDPPI_P (.13.)     0.848    0.069   12.329    0.000    0.787    0.695
##     DDPPI_P (.14.)     0.911    0.071   12.821    0.000    0.846    0.707
##     DDPPI_P (.15.)     0.913    0.069   13.255    0.000    0.848    0.778
##
## Covariances:
##                     Estimate  Std.Err  z-value  P(>|z|)   Std.lv  Std.all
##  .DDPPI_Moral_4 ~~
##    .DDPPI_Prudn_10    -0.036    0.092   -0.388    0.698   -0.036   -0.037
##  .DDPPI_Conven_7 ~~
##    .DDPPI_Persn_15    -0.032    0.060   -0.529    0.597   -0.032   -0.052
##   Moral ~~
##     Conventional       0.694    0.106    6.525    0.000    1.054    1.054
##     Prudential         0.657    0.107    6.132    0.000    0.949    0.949
##     Personal           0.738    0.118    6.275    0.000    0.875    0.875
##   Conventional ~~
##     Prudential         0.577    0.096    6.037    0.000    1.044    1.044
##     Personal           0.636    0.104    6.110    0.000    0.945    0.945
##   Prudential ~~
##     Personal           0.593    0.104    5.681    0.000    0.838    0.838
##
## Intercepts:
##                    Estimate  Std.Err  z-value  P(>|z|)   Std.lv  Std.all
##    .DDPPI_M (.43.)    3.077    0.097   31.637    0.000    3.077    2.572
##    .DDPPI_M (.44.)    2.980    0.098   30.258    0.000    2.980    2.625
##    .DDPPI_M (.45.)    3.149    0.102   30.940    0.000    3.149    2.596
##    .DDPPI_M (.46.)    3.419    0.092   37.106    0.000    3.419    2.897
##    .DDPPI_C (.47.)    3.208    0.090   35.640    0.000    3.208    2.856
##    .DDPPI_C (.48.)    2.788    0.098   28.448    0.000    2.788    2.398
##    .DDPPI_C (.49.)    2.778    0.087   31.837    0.000    2.778    2.517
##    .DDPPI_P (.50.)    3.694    0.091   40.408    0.000    3.694    3.203
##    .DDPPI_P (.51.)    2.859    0.099   28.856    0.000    2.859    2.463
##    .DDPPI_P (.52.)    3.013    0.110   27.323    0.000    3.013    2.145
##    .DDPPI_P (.53.)    2.367    0.099   23.929    0.000    2.367    2.030
##    .DDPPI_P (.54.)    2.391    0.093   25.711    0.000    2.391    2.062
##    .DDPPI_P (.55.)    2.116    0.091   23.136    0.000    2.116    1.869
##    .DDPPI_P (.56.)    2.521    0.097   26.087    0.000    2.521    2.106
##    .DDPPI_P (.57.)    2.304    0.093   24.891    0.000    2.304    2.114
##
## Variances:
##                    Estimate  Std.Err  z-value  P(>|z|)   Std.lv  Std.all
##    .DDPPI_Moral_1     0.606    0.085    7.103    0.000    0.606    0.424
##    .DDPPI_Moral_2     0.324    0.054    6.041    0.000    0.324    0.251
##    .DDPPI_Moral_3     0.488    0.073    6.697    0.000    0.488    0.331
##    .DDPPI_Moral_4     0.753    0.102    7.393    0.000    0.753    0.541
##    .DDPPI_Conven_5    0.736    0.098    7.535    0.000    0.736    0.584
##    .DDPPI_Conven_6    0.625    0.090    6.946    0.000    0.625    0.463
##    .DDPPI_Conven_7    0.787    0.102    7.701    0.000    0.787    0.647
```

```
##    .DDPPI_Pruden_8    0.750    0.106    7.053    0.000    0.750    0.563
##    .DDPPI_Pruden_9    0.560    0.092    6.095    0.000    0.560    0.416
##    .DDPPI_Prudn_10    1.197    0.166    7.206    0.000    1.197    0.607
##    .DDPPI_Persn_11    0.497    0.078    6.349    0.000    0.497    0.366
##    .DDPPI_Persn_12    0.694    0.098    7.068    0.000    0.694    0.516
##    .DDPPI_Persn_13    0.662    0.094    7.067    0.000    0.662    0.516
##    .DDPPI_Persn_14    0.717    0.102    7.014    0.000    0.717    0.500
##    .DDPPI_Persn_15    0.468    0.072    6.507    0.000    0.468    0.394
##     Moral             0.825    0.137    6.025    0.000    1.000    1.000
##     Conventional      0.525    0.108    4.863    0.000    1.000    1.000
##     Prudential        0.581    0.120    4.822    0.000    1.000    1.000
##     Personal          0.862    0.147    5.873    0.000    1.000    1.000
##
##
## Group 2 [US]:
##
## Latent Variables:
##                    Estimate  Std.Err  z-value  P(>|z|)   Std.lv  Std.all
##   Moral =~
##     DDPPI_M           1.000                               0.970    0.830
##     DDPPI_M (.p2.)    1.082    0.061   17.625    0.000    1.049    0.855
##     DDPPI_M (.p3.)    1.092    0.061   17.809    0.000    1.059    0.891
##     DDPPI_M (.p4.)    0.881    0.064   13.838    0.000    0.854    0.746
##   Conventional =~
##     DDPPI_C           1.000                               0.890    0.766
##     DDPPI_C (.p6.)    1.176    0.081   14.548    0.000    1.047    0.850
##     DDPPI_C (.p7.)    0.905    0.079   11.472    0.000    0.806    0.671
##   Prudential =~
##     DDPPI_P           1.000                               0.777    0.758
##     DDPPI_P (.p9.)    1.164    0.099   11.778    0.000    0.904    0.705
##     DDPPI_P (.10.)    1.156    0.110   10.505    0.000    0.898    0.665
##   Personal =~
##     DDPPI_P           1.000                               1.041    0.782
##     DDPPI_P (.12.)    0.869    0.069   12.632    0.000    0.905    0.729
##     DDPPI_P (.13.)    0.848    0.069   12.329    0.000    0.883    0.702
##     DDPPI_P (.14.)    0.911    0.071   12.821    0.000    0.949    0.736
##     DDPPI_P (.15.)    0.913    0.069   13.255    0.000    0.951    0.706
##
## Covariances:
##                      Estimate  Std.Err  z-value  P(>|z|)   Std.lv  Std.all
##  .DDPPI_Moral_4 ~~
##    .DDPPI_Prudn_10    -0.071    0.071   -0.994    0.320   -0.071   -0.092
##  .DDPPI_Conven_7 ~~
##    .DDPPI_Persn_15    -0.006    0.079   -0.079    0.937   -0.006   -0.007
##   Moral ~~
##     Conventional       0.829    0.120    6.937    0.000    0.961    0.961
##     Prudential         0.716    0.106    6.780    0.000    0.950    0.950
##     Personal           0.815    0.127    6.417    0.000    0.807    0.807
##   Conventional ~~
##     Prudential         0.610    0.097    6.264    0.000    0.882    0.882
##     Personal           0.801    0.125    6.413    0.000    0.864    0.864
##   Prudential ~~
##     Personal           0.650    0.108    6.020    0.000    0.804    0.804
##
```

```
## Intercepts:
##                    Estimate  Std.Err  z-value  P(>|z|)   Std.lv  Std.all
##    .DDPPI_M (.43.)    3.077    0.097   31.637    0.000    3.077    2.634
##    .DDPPI_M (.44.)    2.980    0.098   30.258    0.000    2.980    2.428
##    .DDPPI_M (.45.)    3.149    0.102   30.940    0.000    3.149    2.648
##    .DDPPI_M (.46.)    3.419    0.092   37.106    0.000    3.419    2.987
##    .DDPPI_C (.47.)    3.208    0.090   35.640    0.000    3.208    2.761
##    .DDPPI_C (.48.)    2.788    0.098   28.448    0.000    2.788    2.262
##    .DDPPI_C (.49.)    2.778    0.087   31.837    0.000    2.778    2.312
##    .DDPPI_P (.50.)    3.694    0.091   40.408    0.000    3.694    3.602
##    .DDPPI_P (.51.)    2.859    0.099   28.856    0.000    2.859    2.228
##    .DDPPI_P (.52.)    3.013    0.110   27.323    0.000    3.013    2.229
##    .DDPPI_P (.53.)    2.367    0.099   23.929    0.000    2.367    1.777
##    .DDPPI_P (.54.)    2.391    0.093   25.711    0.000    2.391    1.928
##    .DDPPI_P (.55.)    2.116    0.091   23.136    0.000    2.116    1.684
##    .DDPPI_P (.56.)    2.521    0.097   26.087    0.000    2.521    1.957
##    .DDPPI_P (.57.)    2.304    0.093   24.891    0.000    2.304    1.710
##     Moral             0.739    0.125    5.892    0.000    0.762    0.762
##     Cnvntnl           0.764    0.118    6.463    0.000    0.858    0.858
##     Prudntl           0.476    0.112    4.232    0.000    0.612    0.612
##     Personl           0.596    0.133    4.479    0.000    0.572    0.572
##
## Variances:
##                    Estimate  Std.Err  z-value  P(>|z|)   Std.lv  Std.all
##    .DDPPI_Moral_1     0.424    0.058    7.274    0.000    0.424    0.311
##    .DDPPI_Moral_2     0.405    0.058    7.008    0.000    0.405    0.269
##    .DDPPI_Moral_3     0.292    0.046    6.364    0.000    0.292    0.207
##    .DDPPI_Moral_4     0.581    0.075    7.762    0.000    0.581    0.443
##    .DDPPI_Conven_5    0.557    0.076    7.319    0.000    0.557    0.413
##    .DDPPI_Conven_6    0.423    0.069    6.093    0.000    0.423    0.278
##    .DDPPI_Conven_7    0.794    0.101    7.832    0.000    0.794    0.550
##    .DDPPI_Pruden_8    0.448    0.067    6.736    0.000    0.448    0.426
##    .DDPPI_Pruden_9    0.829    0.114    7.285    0.000    0.829    0.503
##    .DDPPI_Prudn_10    1.021    0.136    7.525    0.000    1.021    0.558
##    .DDPPI_Persn_11    0.690    0.101    6.811    0.000    0.690    0.389
##    .DDPPI_Persn_12    0.721    0.099    7.263    0.000    0.721    0.468
##    .DDPPI_Persn_13    0.800    0.108    7.435    0.000    0.800    0.507
##    .DDPPI_Persn_14    0.760    0.105    7.212    0.000    0.760    0.458
##    .DDPPI_Persn_15    0.909    0.123    7.415    0.000    0.909    0.501
##     Moral             0.940    0.143    6.586    0.000    1.000    1.000
##     Conventional      0.793    0.136    5.845    0.000    1.000    1.000
##     Prudential        0.604    0.110    5.495    0.000    1.000    1.000
##     Personal          1.084    0.177    6.115    0.000    1.000    1.000
```

Compare metric and scalar models

Scalar invariance can be rejected if the change in $CFI \geq -.010$ supplemented with the change in $RMSEA \geq .015$ or with the change in $SRMR \geq .010$ (Chen, 2007).

```
Comparison2Like<-compareFit(cfa.metric, cfa.scalar)
summary(Comparison2Like)
```

```
## ################### Nested Model Comparison #########################
```

```
##
## Chi-Squared Difference Test
##
##             Df   AIC   BIC  Chisq Chisq diff    RMSEA Df diff Pr(>Chisq)
## cfa.metric 175 10643 10983 265.51
## cfa.scalar 186 10644 10945 288.83     23.317 0.091754      11    0.01594 *
## ---
## Signif. codes:  0 '***' 0.001 '**' 0.01 '*' 0.05 '.' 0.1 ' ' 1
##
## ####################### Model Fit Indices ###########################
##               chisq  df pvalue rmsea   cfi   tli  srmr        aic        bic
## cfa.metric 265.510† 175   .000 .062† .962† .954† .065† 10642.961† 10983.393
## cfa.scalar 288.826  186   .000 .064  .956  .951  .069  10644.278  10945.292†
##
## ################## Differences in Fit Indices #######################
##                         df rmsea    cfi    tli  srmr   aic     bic
## cfa.scalar - cfa.metric 11 0.002 -0.005 -0.003 0.004 1.317 -38.102
```

Run a MGCFA for DDPPI: Residual Model

```r
cfa.residual <- cfa(model_DDPPI, data=datafile,
                    estimator = "ML", group = "Region",

                    group.equal = c("loadings", "intercepts", "residuals"))
```

Print summary of MGCFA for DDPPI: Residual Model

We used criteria of $CFI > .90$, $RMSEA < .08$, and $SRMR < .08$, which indicate a satisfactory model fit (Hu & Bentler, 1999; Kenny et al., 2015; MacCallum et al., 1996).

```r
summary(cfa.residual, fit.measures = TRUE, standardized = TRUE, ci = FALSE)
```

```
## lavaan 0.6.17 ended normally after 82 iterations
##
##   Estimator                                         ML
##   Optimization method                           NLMINB
##   Number of model parameters                       110
##   Number of equality constraints                    41
##
##   Number of observations per group:
##     HK                                             124
##     US                                             142
##
## Model Test User Model:
##
##   Test statistic                               328.995
##   Degrees of freedom                               201
##   P-value (Chi-square)                           0.000
##   Test statistic for each group:
##     HK                                         179.571
```

```
##     US                                       149.423
##
## Model Test Baseline Model:
##
##   Test statistic                             2567.202
##   Degrees of freedom                              210
##   P-value                                       0.000
##
## User Model versus Baseline Model:
##
##   Comparative Fit Index (CFI)                   0.946
##   Tucker-Lewis Index (TLI)                      0.943
##
## Loglikelihood and Information Criteria:
##
##   Loglikelihood user model (H0)             -5258.223
##   Loglikelihood unrestricted model (H1)     -5093.726
##
##   Akaike (AIC)                              10654.447
##   Bayesian (BIC)                            10901.708
##   Sample-size adjusted Bayesian (SABIC)     10682.939
##
## Root Mean Square Error of Approximation:
##
##   RMSEA                                         0.069
##   90 Percent confidence interval - lower        0.055
##   90 Percent confidence interval - upper        0.082
##   P-value H_0: RMSEA <= 0.050                   0.013
##   P-value H_0: RMSEA >= 0.080                   0.092
##
## Standardized Root Mean Square Residual:
##
##   SRMR                                          0.072
##
## Parameter Estimates:
##
##   Standard errors                            Standard
##   Information                                Expected
##   Information saturated (h1) model         Structured
##
##
## Group 1 [HK]:
##
## Latent Variables:
##                    Estimate  Std.Err  z-value  P(>|z|)   Std.lv  Std.all
##   Moral =~
##     DDPPI_M           1.000                               0.906    0.786
##     DDPPI_M (.p2.)    1.093    0.063   17.453    0.000    0.990    0.854
##     DDPPI_M (.p3.)    1.100    0.064   17.301    0.000    0.997    0.848
##     DDPPI_M (.p4.)    0.897    0.066   13.696    0.000    0.813    0.707
##   Conventional =~
##     DDPPI_C           1.000                               0.753    0.685
##     DDPPI_C (.p6.)    1.159    0.081   14.389    0.000    0.873    0.770
##     DDPPI_C (.p7.)    0.900    0.078   11.523    0.000    0.677    0.608
```

```
##   Prudential =~
##     DDPPI_P           1.000                               0.754    0.698
##     DDPPI_P (.p9.)    1.177    0.101   11.621    0.000    0.888    0.728
##     DDPPI_P (.10.)    1.161    0.113   10.238    0.000    0.875    0.638
##   Personal =~
##     DDPPI_P           1.000                               0.912    0.760
##     DDPPI_P (.12.)    0.875    0.069   12.672    0.000    0.798    0.690
##     DDPPI_P (.13.)    0.851    0.069   12.239    0.000    0.776    0.668
##     DDPPI_P (.14.)    0.915    0.071   12.816    0.000    0.835    0.697
##     DDPPI_P (.15.)    0.890    0.070   12.718    0.000    0.811    0.693
##
## Covariances:
##                     Estimate  Std.Err  z-value  P(>|z|)   Std.lv  Std.all
##  .DDPPI_Moral_4 ~~
##    .DDPPI_Prudn_10    -0.036    0.084   -0.429    0.668   -0.036   -0.042
##  .DDPPI_Conven_7 ~~
##    .DDPPI_Persn_15    -0.035    0.072   -0.490    0.624   -0.035   -0.047
##   Moral ~~
##     Conventional       0.694    0.107    6.512    0.000    1.017    1.017
##     Prudential         0.645    0.104    6.177    0.000    0.944    0.944
##     Personal           0.727    0.116    6.250    0.000    0.880    0.880
##   Conventional ~~
##     Prudential         0.578    0.095    6.081    0.000    1.017    1.017
##     Personal           0.650    0.106    6.142    0.000    0.946    0.946
##   Prudential ~~
##     Personal           0.588    0.103    5.719    0.000    0.854    0.854
##
## Intercepts:
##                    Estimate  Std.Err  z-value  P(>|z|)   Std.lv  Std.all
##    .DDPPI_M (.43.)    3.074    0.096   32.017    0.000    3.074    2.668
##    .DDPPI_M (.44.)    2.974    0.100   29.811    0.000    2.974    2.566
##    .DDPPI_M (.45.)    3.155    0.101   31.289    0.000    3.155    2.686
##    .DDPPI_M (.46.)    3.400    0.092   36.812    0.000    3.400    2.957
##    .DDPPI_C (.47.)    3.205    0.090   35.435    0.000    3.205    2.913
##    .DDPPI_C (.48.)    2.783    0.097   28.638    0.000    2.783    2.455
##    .DDPPI_C (.49.)    2.777    0.088   31.411    0.000    2.777    2.492
##    .DDPPI_P (.50.)    3.683    0.089   41.473    0.000    3.683    3.410
##    .DDPPI_P (.51.)    2.849    0.102   27.968    0.000    2.849    2.338
##    .DDPPI_P (.52.)    3.013    0.109   27.598    0.000    3.013    2.197
##    .DDPPI_P (.53.)    2.378    0.099   23.905    0.000    2.378    1.982
##    .DDPPI_P (.54.)    2.387    0.093   25.799    0.000    2.387    2.064
##    .DDPPI_P (.55.)    2.113    0.092   23.015    0.000    2.113    1.821
##    .DDPPI_P (.56.)    2.521    0.096   26.241    0.000    2.521    2.107
##    .DDPPI_P (.57.)    2.313    0.094   24.661    0.000    2.313    1.976
##
## Variances:
##                    Estimate  Std.Err  z-value  P(>|z|)   Std.lv  Std.all
##    .DDPPI_M (.18.)    0.507    0.050   10.150    0.000    0.507    0.382
##    .DDPPI_M (.19.)    0.363    0.040    9.154    0.000    0.363    0.271
##    .DDPPI_M (.20.)    0.386    0.042    9.275    0.000    0.386    0.280
##    .DDPPI_M (.21.)    0.662    0.062   10.671    0.000    0.662    0.501
##    .DDPPI_C (.22.)    0.643    0.061   10.499    0.000    0.643    0.531
##    .DDPPI_C (.23.)    0.523    0.056    9.370    0.000    0.523    0.407
##    .DDPPI_C (.24.)    0.783    0.072   10.951    0.000    0.783    0.630
```

```
##    .DDPPI_P (.25.)    0.598    0.061    9.877    0.000    0.598    0.513
##    .DDPPI_P (.26.)    0.697    0.074    9.469    0.000    0.697    0.469
##    .DDPPI_P (.27.)    1.115    0.107   10.399    0.000    1.115    0.593
##    .DDPPI_P (.28.)    0.607    0.066    9.263    0.000    0.607    0.422
##    .DDPPI_P (.29.)    0.701    0.070   10.041    0.000    0.701    0.524
##    .DDPPI_P (.30.)    0.745    0.073   10.211    0.000    0.745    0.553
##    .DDPPI_P (.31.)    0.736    0.074    9.978    0.000    0.736    0.514
##    .DDPPI_P (.32.)    0.712    0.071   10.000    0.000    0.712    0.520
##     Moral             0.821    0.135    6.087    0.000    1.000    1.000
##     Cnvntnl           0.567    0.110    5.134    0.000    1.000    1.000
##     Prudntl           0.569    0.115    4.942    0.000    1.000    1.000
##     Personl           0.832    0.146    5.706    0.000    1.000    1.000
##
##
## Group 2 [US]:
##
## Latent Variables:
##                    Estimate  Std.Err  z-value  P(>|z|)   Std.lv  Std.all
##   Moral =~
##     DDPPI_M           1.000                               0.957    0.802
##     DDPPI_M (.p2.)    1.093    0.063   17.453    0.000    1.045    0.866
##     DDPPI_M (.p3.)    1.100    0.064   17.301    0.000    1.053    0.861
##     DDPPI_M (.p4.)    0.897    0.066   13.696    0.000    0.858    0.726
##   Conventional =~
##     DDPPI_C           1.000                               0.889    0.742
##     DDPPI_C (.p6.)    1.159    0.081   14.389    0.000    1.030    0.818
##     DDPPI_C (.p7.)    0.900    0.078   11.523    0.000    0.799    0.670
##   Prudential =~
##     DDPPI_P           1.000                               0.766    0.704
##     DDPPI_P (.p9.)    1.177    0.101   11.621    0.000    0.902    0.734
##     DDPPI_P (.10.)    1.161    0.113   10.238    0.000    0.890    0.644
##   Personal =~
##     DDPPI_P           1.000                               1.049    0.803
##     DDPPI_P (.12.)    0.875    0.069   12.672    0.000    0.919    0.739
##     DDPPI_P (.13.)    0.851    0.069   12.239    0.000    0.892    0.719
##     DDPPI_P (.14.)    0.915    0.071   12.816    0.000    0.960    0.746
##     DDPPI_P (.15.)    0.890    0.070   12.718    0.000    0.933    0.742
##
## Covariances:
##                      Estimate  Std.Err  z-value  P(>|z|)   Std.lv  Std.all
##  .DDPPI_Moral_4 ~~
##    .DDPPI_Prudn_10    -0.060    0.078   -0.761    0.447   -0.060   -0.069
##  .DDPPI_Conven_7 ~~
##    .DDPPI_Persn_15    -0.013    0.069   -0.181    0.856   -0.013   -0.017
##   Moral ~~
##     Conventional       0.841    0.121    6.942    0.000    0.989    0.989
##     Prudential         0.702    0.106    6.617    0.000    0.957    0.957
##     Personal           0.812    0.127    6.415    0.000    0.809    0.809
##   Conventional ~~
##     Prudential         0.616    0.100    6.174    0.000    0.905    0.905
##     Personal           0.811    0.127    6.414    0.000    0.870    0.870
##   Prudential ~~
##     Personal           0.655    0.110    5.974    0.000    0.814    0.814
##
```

```
## Intercepts:
##                    Estimate  Std.Err  z-value  P(>|z|)   Std.lv  Std.all
##    .DDPPI_M (.43.)    3.074    0.096   32.017    0.000    3.074    2.578
##    .DDPPI_M (.44.)    2.974    0.100   29.811    0.000    2.974    2.464
##    .DDPPI_M (.45.)    3.155    0.101   31.289    0.000    3.155    2.581
##    .DDPPI_M (.46.)    3.400    0.092   36.812    0.000    3.400    2.875
##    .DDPPI_C (.47.)    3.205    0.090   35.435    0.000    3.205    2.677
##    .DDPPI_C (.48.)    2.783    0.097   28.638    0.000    2.783    2.211
##    .DDPPI_C (.49.)    2.777    0.088   31.411    0.000    2.777    2.328
##    .DDPPI_P (.50.)    3.683    0.089   41.473    0.000    3.683    3.383
##    .DDPPI_P (.51.)    2.849    0.102   27.968    0.000    2.849    2.318
##    .DDPPI_P (.52.)    3.013    0.109   27.598    0.000    3.013    2.182
##    .DDPPI_P (.53.)    2.378    0.099   23.905    0.000    2.378    1.820
##    .DDPPI_P (.54.)    2.387    0.093   25.799    0.000    2.387    1.921
##    .DDPPI_P (.55.)    2.113    0.092   23.015    0.000    2.113    1.702
##    .DDPPI_P (.56.)    2.521    0.096   26.241    0.000    2.521    1.958
##    .DDPPI_P (.57.)    2.313    0.094   24.661    0.000    2.313    1.838
##     Moral             0.734    0.125    5.895    0.000    0.767    0.767
##     Cnvntnl           0.770    0.120    6.438    0.000    0.867    0.867
##     Prudntl           0.474    0.111    4.250    0.000    0.618    0.618
##     Personl           0.595    0.133    4.479    0.000    0.567    0.567
##
## Variances:
##                    Estimate  Std.Err  z-value  P(>|z|)   Std.lv  Std.all
##    .DDPPI_M (.18.)    0.507    0.050   10.150    0.000    0.507    0.356
##    .DDPPI_M (.19.)    0.363    0.040    9.154    0.000    0.363    0.250
##    .DDPPI_M (.20.)    0.386    0.042    9.275    0.000    0.386    0.259
##    .DDPPI_M (.21.)    0.662    0.062   10.671    0.000    0.662    0.473
##    .DDPPI_C (.22.)    0.643    0.061   10.499    0.000    0.643    0.449
##    .DDPPI_C (.23.)    0.523    0.056    9.370    0.000    0.523    0.330
##    .DDPPI_C (.24.)    0.783    0.072   10.951    0.000    0.783    0.551
##    .DDPPI_P (.25.)    0.598    0.061    9.877    0.000    0.598    0.505
##    .DDPPI_P (.26.)    0.697    0.074    9.469    0.000    0.697    0.461
##    .DDPPI_P (.27.)    1.115    0.107   10.399    0.000    1.115    0.585
##    .DDPPI_P (.28.)    0.607    0.066    9.263    0.000    0.607    0.355
##    .DDPPI_P (.29.)    0.701    0.070   10.041    0.000    0.701    0.454
##    .DDPPI_P (.30.)    0.745    0.073   10.211    0.000    0.745    0.483
##    .DDPPI_P (.31.)    0.736    0.074    9.978    0.000    0.736    0.444
##    .DDPPI_P (.32.)    0.712    0.071   10.000    0.000    0.712    0.450
##     Moral             0.915    0.142    6.459    0.000    1.000    1.000
##     Cnvntnl           0.790    0.138    5.726    0.000    1.000    1.000
##     Prudntl           0.587    0.112    5.222    0.000    1.000    1.000
##     Personl           1.101    0.178    6.193    0.000    1.000    1.000
```

Compare scalar and residual models

Residual invariance can be rejected if the change in $CFI \geq -.010$ supplemented with the change in $RMSEA \geq .015$ or with the change in $SRMR \geq .010$ (Chen, 2007).

```
Comparison3Like<-compareFit(cfa.scalar,cfa.residual)
summary(Comparison3Like)
```

```
## ################### Nested Model Comparison #########################
```

```
##
## Chi-Squared Difference Test
##
##               Df   AIC   BIC  Chisq Chisq diff   RMSEA Df diff Pr(>Chisq)
## cfa.scalar   186 10644 10945 288.83
## cfa.residual 201 10654 10902 328.99     40.169 0.11232      15  0.0004277 ***
## ---
## Signif. codes:  0 '***' 0.001 '**' 0.01 '*' 0.05 '.' 0.1 ' ' 1
##
## ####################### Model Fit Indices ###########################
##                 chisq  df pvalue rmsea   cfi   tli  srmr        aic        bic
## cfa.scalar   288.826† 186   .000 .064† .956† .951† .069† 10644.278† 10945.292
## cfa.residual 328.995  201   .000 .069  .946  .943  .072  10654.447  10901.708†
##
## ################## Differences in Fit Indices #######################
##                           df rmsea    cfi    tli  srmr    aic     bic
## cfa.residual - cfa.scalar 15 0.005 -0.011 -0.007 0.003 10.169 -43.584
```

Compute composite reliability for DDPPI

```
compRelSEM(cfa.scalar,return.total = TRUE)
```

```
##   group Moral Conventional Prudential Personal total
## 1    HK 0.866        0.703      0.728    0.861 0.949
## 2    US 0.921        0.779      0.736    0.853 0.944
```

Specify the model parameters for NRI-SPV

Modification indices suggested the presence of correlated residuals, and thus, residuals for four items across two paths were allowed to correlate.

```
model_NRI <- '
Conflict=~NRI_SPV_Con_1+NRI_SPV_Con_2+NRI_SPV_Con_3
Antagonistic=~NRI_SPV_Ant_4+NRI_SPV_Ant_5+NRI_SPV_Ant_6
Affection=~ NRI_SPV_Aff_7+NRI_SPV_Aff_8+NRI_SPV_Aff_9
Reassurance=~ NRI_SPV_RS_10+NRI_SPV_RS_11+NRI_SPV_RS_12
Satisfaction=~NRI_SPV_Sat_13+NRI_SPV_Sat_14+NRI_SPV_Sat_15
NRI_SPV_Aff_7 ~~  NRI_SPV_Aff_8
NRI_SPV_Aff_7 ~~  NRI_SPV_RS_11
'
```

Run a MGCFA for NRI-SPV: Configural Model

```
cfa.model <- cfa(model_NRI, data=datafile,
                 estimator = "ML", group = "Region")
```

Print summary of MGCFA for NRI-SPV: Configural Model

We used criteria of $CFI > .90$, $RMSEA < .08$, and $SRMR < .08$, which indicate a satisfactory model fit (Hu & Bentler, 1999; Kenny et al., 2015; MacCallum et al., 1996).

```r
summary(cfa.model, fit.measures = TRUE, standardized = TRUE, ci = FALSE)
```

```
## lavaan 0.6.17 ended normally after 76 iterations
##
##   Estimator                                         ML
##   Optimization method                           NLMINB
##   Number of model parameters                       114
##
##   Number of observations per group:
##     HK                                             124
##     US                                             142
##
## Model Test User Model:
##
##   Test statistic                               247.908
##   Degrees of freedom                               156
##   P-value (Chi-square)                           0.000
##   Test statistic for each group:
##     HK                                         114.040
##     US                                         133.868
##
## Model Test Baseline Model:
##
##   Test statistic                              3641.528
##   Degrees of freedom                               210
##   P-value                                        0.000
##
## User Model versus Baseline Model:
##
##   Comparative Fit Index (CFI)                    0.973
##   Tucker-Lewis Index (TLI)                       0.964
##
## Loglikelihood and Information Criteria:
##
##   Loglikelihood user model (H0)              -4079.860
##   Loglikelihood unrestricted model (H1)      -3955.906
##
##   Akaike (AIC)                                8387.721
##   Bayesian (BIC)                              8796.239
##   Sample-size adjusted Bayesian (SABIC)       8434.795
##
## Root Mean Square Error of Approximation:
##
##   RMSEA                                          0.067
##   90 Percent confidence interval - lower         0.051
##   90 Percent confidence interval - upper         0.082
##   P-value H_0: RMSEA <= 0.050                    0.045
##   P-value H_0: RMSEA >= 0.080                    0.075
##
```

```
## Standardized Root Mean Square Residual:
##
##   SRMR                                           0.041
##
## Parameter Estimates:
##
##   Standard errors                             Standard
##   Information                                 Expected
##   Information saturated (h1) model          Structured
##
##
## Group 1 [HK]:
##
## Latent Variables:
##                    Estimate  Std.Err  z-value  P(>|z|)   Std.lv  Std.all
##   Conflict =~
##     NRI_SPV_Con_1     1.000                               0.863    0.897
##     NRI_SPV_Con_2     0.975    0.071   13.713    0.000    0.842    0.863
##     NRI_SPV_Con_3     0.983    0.066   14.902    0.000    0.848    0.898
##   Antagonistic =~
##     NRI_SPV_Ant_4     1.000                               0.767    0.797
##     NRI_SPV_Ant_5     1.201    0.102   11.758    0.000    0.922    0.920
##     NRI_SPV_Ant_6     0.897    0.117    7.656    0.000    0.688    0.652
##   Affection =~
##     NRI_SPV_Aff_7     1.000                               0.880    0.930
##     NRI_SPV_Aff_8     0.951    0.080   11.898    0.000    0.837    0.869
##     NRI_SPV_Aff_9     0.899    0.092    9.802    0.000    0.792    0.759
##   Reassurance =~
##     NRI_SPV_RS_10     1.000                               0.995    0.910
##     NRI_SPV_RS_11     0.781    0.080    9.724    0.000    0.777    0.729
##     NRI_SPV_RS_12     0.690    0.061   11.289    0.000    0.687    0.792
##   Satisfaction =~
##     NRI_SPV_Sat_13    1.000                               0.867    0.934
##     NRI_SPV_Sat_14    0.846    0.074   11.471    0.000    0.734    0.774
##     NRI_SPV_Sat_15    0.977    0.064   15.389    0.000    0.848    0.896
##
## Covariances:
##                    Estimate  Std.Err  z-value  P(>|z|)   Std.lv  Std.all
##  .NRI_SPV_Aff_7 ~~
##    .NRI_SPV_Aff_8    -0.074    0.046   -1.594    0.111   -0.074   -0.444
##    .NRI_SPV_RS_11    -0.052    0.038   -1.382    0.167   -0.052   -0.204
##   Conflict ~~
##     Antagonistic      0.616    0.098    6.300    0.000    0.929    0.929
##     Affection        -0.140    0.075   -1.878    0.060   -0.184   -0.184
##     Reassurance      -0.303    0.091   -3.334    0.001   -0.353   -0.353
##     Satisfaction     -0.360    0.082   -4.420    0.000   -0.481   -0.481
##   Antagonistic ~~
##     Affection        -0.102    0.067   -1.523    0.128   -0.151   -0.151
##     Reassurance      -0.279    0.084   -3.334    0.001   -0.365   -0.365
##     Satisfaction     -0.328    0.076   -4.326    0.000   -0.493   -0.493
##   Affection ~~
##     Reassurance       0.738    0.113    6.525    0.000    0.842    0.842
##     Satisfaction      0.504    0.089    5.690    0.000    0.661    0.661
##   Reassurance ~~
```

```
##     Satisfaction      0.621    0.105    5.913    0.000    0.719    0.719
##
## Intercepts:
##                    Estimate  Std.Err  z-value  P(>|z|)   Std.lv  Std.all
##    .NRI_SPV_Con_1     2.540    0.086   29.400    0.000    2.540    2.640
##    .NRI_SPV_Con_2     2.403    0.088   27.452    0.000    2.403    2.465
##    .NRI_SPV_Con_3     2.452    0.085   28.892    0.000    2.452    2.595
##    .NRI_SPV_Ant_4     2.677    0.087   30.946    0.000    2.677    2.779
##    .NRI_SPV_Ant_5     2.645    0.090   29.409    0.000    2.645    2.641
##    .NRI_SPV_Ant_6     2.984    0.095   31.500    0.000    2.984    2.829
##    .NRI_SPV_Aff_7     3.960    0.085   46.560    0.000    3.960    4.181
##    .NRI_SPV_Aff_8     3.718    0.087   42.966    0.000    3.718    3.858
##    .NRI_SPV_Aff_9     3.540    0.094   37.811    0.000    3.540    3.396
##    .NRI_SPV_RS_10     3.573    0.098   36.372    0.000    3.573    3.266
##    .NRI_SPV_RS_11     3.371    0.096   35.218    0.000    3.371    3.163
##    .NRI_SPV_RS_12     3.323    0.078   42.700    0.000    3.323    3.835
##    .NRI_SPV_Sat_13    3.532    0.083   42.369    0.000    3.532    3.805
##    .NRI_SPV_Sat_14    3.387    0.085   39.790    0.000    3.387    3.573
##    .NRI_SPV_Sat_15    3.492    0.085   41.100    0.000    3.492    3.691
##
## Variances:
##                    Estimate  Std.Err  z-value  P(>|z|)   Std.lv  Std.all
##    .NRI_SPV_Con_1     0.180    0.033    5.535    0.000    0.180    0.195
##    .NRI_SPV_Con_2     0.242    0.039    6.235    0.000    0.242    0.254
##    .NRI_SPV_Con_3     0.173    0.031    5.521    0.000    0.173    0.194
##    .NRI_SPV_Ant_4     0.339    0.051    6.701    0.000    0.339    0.365
##    .NRI_SPV_Ant_5     0.153    0.040    3.871    0.000    0.153    0.153
##    .NRI_SPV_Ant_6     0.639    0.086    7.391    0.000    0.639    0.575
##    .NRI_SPV_Aff_7     0.122    0.053    2.298    0.022    0.122    0.136
##    .NRI_SPV_Aff_8     0.227    0.060    3.766    0.000    0.227    0.245
##    .NRI_SPV_Aff_9     0.461    0.068    6.727    0.000    0.461    0.424
##    .NRI_SPV_RS_10     0.206    0.051    4.009    0.000    0.206    0.172
##    .NRI_SPV_RS_11     0.533    0.077    6.921    0.000    0.533    0.469
##    .NRI_SPV_RS_12     0.279    0.042    6.577    0.000    0.279    0.372
##    .NRI_SPV_Sat_13    0.110    0.031    3.594    0.000    0.110    0.127
##    .NRI_SPV_Sat_14    0.360    0.052    6.975    0.000    0.360    0.401
##    .NRI_SPV_Sat_15    0.177    0.035    5.082    0.000    0.177    0.197
##     Conflict          0.745    0.118    6.337    0.000    1.000    1.000
##     Antagonistic      0.589    0.113    5.217    0.000    1.000    1.000
##     Affection         0.775    0.124    6.262    0.000    1.000    1.000
##     Reassurance       0.991    0.156    6.347    0.000    1.000    1.000
##     Satisfaction      0.752    0.112    6.721    0.000    1.000    1.000
##
##
## Group 2 [US]:
##
## Latent Variables:
##                    Estimate  Std.Err  z-value  P(>|z|)   Std.lv  Std.all
##   Conflict =~
##     NRI_SPV_Con_1     1.000                               0.967    0.870
##     NRI_SPV_Con_2     0.938    0.070   13.432    0.000    0.908    0.857
##     NRI_SPV_Con_3     0.939    0.068   13.870    0.000    0.909    0.872
##   Antagonistic =~
##     NRI_SPV_Ant_4     1.000                               0.973    0.824
```

```
##     NRI_SPV_Ant_5     0.969    0.090   10.722    0.000    0.943    0.792
##     NRI_SPV_Ant_6     0.967    0.084   11.474    0.000    0.941    0.831
##   Affection =~
##     NRI_SPV_Aff_7     1.000                               0.703    0.832
##     NRI_SPV_Aff_8     1.012    0.044   22.851    0.000    0.711    0.838
##     NRI_SPV_Aff_9     1.403    0.113   12.432    0.000    0.986    0.889
##   Reassurance =~
##     NRI_SPV_RS_10     1.000                               1.045    0.898
##     NRI_SPV_RS_11     0.904    0.067   13.478    0.000    0.945    0.843
##     NRI_SPV_RS_12     0.732    0.065   11.324    0.000    0.765    0.762
##   Satisfaction =~
##     NRI_SPV_Sat_13    1.000                               1.075    0.959
##     NRI_SPV_Sat_14    0.999    0.045   22.168    0.000    1.074    0.924
##     NRI_SPV_Sat_15    0.972    0.043   22.479    0.000    1.046    0.927
##
## Covariances:
##                    Estimate  Std.Err  z-value  P(>|z|)   Std.lv  Std.all
##  .NRI_SPV_Aff_7 ~~
##    .NRI_SPV_Aff_8     0.158    0.032    4.887    0.000    0.158    0.726
##    .NRI_SPV_RS_11    -0.052    0.019   -2.722    0.006   -0.052   -0.184
##   Conflict ~~
##     Antagonistic      0.883    0.130    6.799    0.000    0.939    0.939
##     Affection        -0.178    0.067   -2.642    0.008   -0.262   -0.262
##     Reassurance      -0.436    0.104   -4.190    0.000   -0.431   -0.431
##     Satisfaction     -0.508    0.106   -4.803    0.000   -0.488   -0.488
##   Antagonistic ~~
##     Affection        -0.215    0.071   -3.037    0.002   -0.314   -0.314
##     Reassurance      -0.471    0.109   -4.326    0.000   -0.464   -0.464
##     Satisfaction     -0.534    0.110   -4.836    0.000   -0.511   -0.511
##   Affection ~~
##     Reassurance       0.644    0.097    6.666    0.000    0.877    0.877
##     Satisfaction      0.600    0.092    6.531    0.000    0.794    0.794
##   Reassurance ~~
##     Satisfaction      0.897    0.131    6.846    0.000    0.798    0.798
##
## Intercepts:
##                    Estimate  Std.Err  z-value  P(>|z|)   Std.lv  Std.all
##    .NRI_SPV_Con_1     2.507    0.093   26.873    0.000    2.507    2.255
##    .NRI_SPV_Con_2     2.331    0.089   26.213    0.000    2.331    2.200
##    .NRI_SPV_Con_3     2.345    0.087   26.826    0.000    2.345    2.251
##    .NRI_SPV_Ant_4     2.606    0.099   26.300    0.000    2.606    2.207
##    .NRI_SPV_Ant_5     2.768    0.100   27.697    0.000    2.768    2.324
##    .NRI_SPV_Ant_6     2.394    0.095   25.208    0.000    2.394    2.115
##    .NRI_SPV_Aff_7     4.613    0.071   65.040    0.000    4.613    5.458
##    .NRI_SPV_Aff_8     4.592    0.071   64.460    0.000    4.592    5.409
##    .NRI_SPV_Aff_9     4.225    0.093   45.383    0.000    4.225    3.808
##    .NRI_SPV_RS_10     3.923    0.098   40.183    0.000    3.923    3.372
##    .NRI_SPV_RS_11     3.866    0.094   41.107    0.000    3.866    3.450
##    .NRI_SPV_RS_12     3.859    0.084   45.797    0.000    3.859    3.843
##    .NRI_SPV_Sat_13    3.845    0.094   40.857    0.000    3.845    3.429
##    .NRI_SPV_Sat_14    3.732    0.098   38.266    0.000    3.732    3.211
##    .NRI_SPV_Sat_15    3.803    0.095   40.196    0.000    3.803    3.373
##
## Variances:
```

```
##                    Estimate  Std.Err  z-value  P(>|z|)   Std.lv  Std.all
##    .NRI_SPV_Con_1     0.300    0.048    6.208    0.000    0.300    0.243
##    .NRI_SPV_Con_2     0.299    0.046    6.473    0.000    0.299    0.266
##    .NRI_SPV_Con_3     0.259    0.042    6.162    0.000    0.259    0.239
##    .NRI_SPV_Ant_4     0.448    0.068    6.562    0.000    0.448    0.321
##    .NRI_SPV_Ant_5     0.529    0.076    6.967    0.000    0.529    0.373
##    .NRI_SPV_Ant_6     0.396    0.061    6.447    0.000    0.396    0.309
##    .NRI_SPV_Aff_7     0.220    0.036    6.159    0.000    0.220    0.308
##    .NRI_SPV_Aff_8     0.215    0.035    6.136    0.000    0.215    0.298
##    .NRI_SPV_Aff_9     0.258    0.054    4.828    0.000    0.258    0.210
##    .NRI_SPV_RS_10     0.261    0.051    5.128    0.000    0.261    0.193
##    .NRI_SPV_RS_11     0.363    0.056    6.447    0.000    0.363    0.289
##    .NRI_SPV_RS_12     0.423    0.057    7.382    0.000    0.423    0.419
##    .NRI_SPV_Sat_13    0.102    0.023    4.388    0.000    0.102    0.081
##    .NRI_SPV_Sat_14    0.197    0.031    6.298    0.000    0.197    0.146
##    .NRI_SPV_Sat_15    0.178    0.029    6.178    0.000    0.178    0.140
##     Conflict          0.936    0.146    6.412    0.000    1.000    1.000
##     Antagonistic      0.946    0.162    5.826    0.000    1.000    1.000
##     Affection         0.494    0.084    5.867    0.000    1.000    1.000
##     Reassurance       1.092    0.163    6.715    0.000    1.000    1.000
##     Satisfaction      1.156    0.150    7.704    0.000    1.000    1.000
```

Run a MGCFA for NRI-SPV: Metric Model

```
cfa.metric <- cfa(model_NRI, data=datafile,
                  estimator = "ML", group = "Region",

                  group.equal= "loadings", parameterization = "delta")
```

Print summary of MGCFA for NRI-SPV: Metric Model

We used criteria of $CFI > .90$, $RMSEA < .08$, and $SRMR < .08$, which indicate a satisfactory model fit (Hu & Bentler, 1999; Kenny et al., 2015; MacCallum et al., 1996).

```
summary(cfa.metric, fit.measures = TRUE, standardized = TRUE, ci = FALSE)
```

```
## lavaan 0.6.17 ended normally after 74 iterations
##
##   Estimator                                         ML
##   Optimization method                           NLMINB
##   Number of model parameters                       114
##   Number of equality constraints                    10
##
##   Number of observations per group:
##     HK                                             124
##     US                                             142
##
## Model Test User Model:
##
##   Test statistic                               270.291
```

```
##   Degrees of freedom                               166
##   P-value (Chi-square)                           0.000
##   Test statistic for each group:
##     HK                                         127.006
##     US                                         143.284
##
## Model Test Baseline Model:
##
##   Test statistic                              3641.528
##   Degrees of freedom                               210
##   P-value                                        0.000
##
## User Model versus Baseline Model:
##
##   Comparative Fit Index (CFI)                    0.970
##   Tucker-Lewis Index (TLI)                       0.962
##
## Loglikelihood and Information Criteria:
##
##   Loglikelihood user model (H0)              -4091.052
##   Loglikelihood unrestricted model (H1)      -3955.906
##
##   Akaike (AIC)                                8390.103
##   Bayesian (BIC)                              8762.787
##   Sample-size adjusted Bayesian (SABIC)       8433.048
##
## Root Mean Square Error of Approximation:
##
##   RMSEA                                          0.069
##   90 Percent confidence interval - lower         0.053
##   90 Percent confidence interval - upper         0.083
##   P-value H_0: RMSEA <= 0.050                    0.023
##   P-value H_0: RMSEA >= 0.080                    0.105
##
## Standardized Root Mean Square Residual:
##
##   SRMR                                           0.055
##
## Parameter Estimates:
##
##   Standard errors                             Standard
##   Information                                 Expected
##   Information saturated (h1) model          Structured
##
##
## Group 1 [HK]:
##
## Latent Variables:
##                    Estimate  Std.Err  z-value  P(>|z|)   Std.lv  Std.all
##   Conflict =~
##     NRI_SPV           1.000                               0.875    0.902
##     NRI_SPV (.p2.)    0.956    0.050   19.185    0.000    0.836    0.861
##     NRI_SPV (.p3.)    0.961    0.047   20.348    0.000    0.841    0.895
##   Antagonistic =~
```

```
##     NRI_SPV           1.000                                   0.804    0.812
##     NRI_SPV (.p5.)    1.091    0.067   16.283    0.000    0.877    0.902
##     NRI_SPV (.p6.)    0.957    0.069   13.896    0.000    0.769    0.696
##   Affection =~
##     NRI_SPV           1.000                                   0.800    0.880
##     NRI_SPV (.p8.)    0.991    0.037   27.093    0.000    0.793    0.834
##     NRI_SPV (.p9.)    1.158    0.070   16.534    0.000    0.927    0.816
##   Reassurance =~
##     NRI_SPV           1.000                                   0.971    0.899
##     NRI_SPV (.11.)    0.856    0.051   16.656    0.000    0.831    0.754
##     NRI_SPV (.12.)    0.714    0.045   15.928    0.000    0.693    0.797
##   Satisfaction =~
##     NRI_SPV           1.000                                   0.850    0.928
##     NRI_SPV (.14.)    0.962    0.038   25.029    0.000    0.818    0.809
##     NRI_SPV (.15.)    0.975    0.036   27.238    0.000    0.828    0.890
##
## Covariances:
##                    Estimate  Std.Err  z-value  P(>|z|)   Std.lv  Std.all
##  .NRI_SPV_Aff_7 ~~
##    .NRI_SPV_Aff_8    -0.019    0.038   -0.495    0.621   -0.019   -0.082
##    .NRI_SPV_RS_11    -0.053    0.038   -1.411    0.158   -0.053   -0.170
##   Conflict ~~
##     Antagonistic      0.656    0.097    6.733    0.000    0.932    0.932
##     Affection        -0.132    0.071   -1.874    0.061   -0.189   -0.189
##     Reassurance      -0.299    0.090   -3.334    0.001   -0.352   -0.352
##     Satisfaction     -0.357    0.080   -4.458    0.000   -0.480   -0.480
##   Antagonistic ~~
##     Affection        -0.101    0.066   -1.523    0.128   -0.156   -0.156
##     Reassurance      -0.290    0.085   -3.413    0.001   -0.372   -0.372
##     Satisfaction     -0.340    0.076   -4.462    0.000   -0.498   -0.498
##   Affection ~~
##     Reassurance       0.672    0.101    6.642    0.000    0.865    0.865
##     Satisfaction      0.466    0.080    5.807    0.000    0.685    0.685
##   Reassurance ~~
##     Satisfaction      0.600    0.100    6.028    0.000    0.728    0.728
##
## Intercepts:
##                    Estimate  Std.Err  z-value  P(>|z|)   Std.lv  Std.all
##    .NRI_SPV_Con_1     2.540    0.087   29.154    0.000    2.540    2.618
##    .NRI_SPV_Con_2     2.403    0.087   27.564    0.000    2.403    2.475
##    .NRI_SPV_Con_3     2.452    0.084   29.056    0.000    2.452    2.609
##    .NRI_SPV_Ant_4     2.677    0.089   30.139    0.000    2.677    2.707
##    .NRI_SPV_Ant_5     2.645    0.087   30.285    0.000    2.645    2.720
##    .NRI_SPV_Ant_6     2.984    0.099   30.069    0.000    2.984    2.700
##    .NRI_SPV_Aff_7     3.960    0.082   48.497    0.000    3.960    4.355
##    .NRI_SPV_Aff_8     3.718    0.085   43.533    0.000    3.718    3.909
##    .NRI_SPV_Aff_9     3.540    0.102   34.708    0.000    3.540    3.117
##    .NRI_SPV_RS_10     3.573    0.097   36.830    0.000    3.573    3.307
##    .NRI_SPV_RS_11     3.371    0.099   34.060    0.000    3.371    3.059
##    .NRI_SPV_RS_12     3.323    0.078   42.526    0.000    3.323    3.819
##    .NRI_SPV_Sat_13    3.532    0.082   42.939    0.000    3.532    3.856
##    .NRI_SPV_Sat_14    3.387    0.091   37.319    0.000    3.387    3.351
##    .NRI_SPV_Sat_15    3.492    0.084   41.754    0.000    3.492    3.750
##
```

```
## Variances:
##                    Estimate  Std.Err  z-value  P(>|z|)   Std.lv  Std.all
##    .NRI_SPV_Con_1     0.176    0.032    5.478    0.000    0.176    0.187
##    .NRI_SPV_Con_2     0.243    0.039    6.311    0.000    0.243    0.258
##    .NRI_SPV_Con_3     0.176    0.031    5.662    0.000    0.176    0.199
##    .NRI_SPV_Ant_4     0.333    0.051    6.558    0.000    0.333    0.340
##    .NRI_SPV_Ant_5     0.176    0.038    4.673    0.000    0.176    0.186
##    .NRI_SPV_Ant_6     0.629    0.087    7.262    0.000    0.629    0.515
##    .NRI_SPV_Aff_7     0.186    0.044    4.275    0.000    0.186    0.225
##    .NRI_SPV_Aff_8     0.275    0.053    5.175    0.000    0.275    0.304
##    .NRI_SPV_Aff_9     0.431    0.069    6.270    0.000    0.431    0.334
##    .NRI_SPV_RS_10     0.224    0.049    4.527    0.000    0.224    0.192
##    .NRI_SPV_RS_11     0.524    0.077    6.831    0.000    0.524    0.432
##    .NRI_SPV_RS_12     0.277    0.042    6.564    0.000    0.277    0.366
##    .NRI_SPV_Sat_13    0.117    0.028    4.147    0.000    0.117    0.139
##    .NRI_SPV_Sat_14    0.353    0.052    6.762    0.000    0.353    0.345
##    .NRI_SPV_Sat_15    0.181    0.033    5.482    0.000    0.181    0.209
##     Conflict          0.765    0.114    6.733    0.000    1.000    1.000
##     Antagonistic      0.646    0.108    5.974    0.000    1.000    1.000
##     Affection         0.640    0.098    6.532    0.000    1.000    1.000
##     Reassurance       0.943    0.144    6.566    0.000    1.000    1.000
##     Satisfaction      0.722    0.102    7.056    0.000    1.000    1.000
##
##
## Group 2 [US]:
##
## Latent Variables:
##                    Estimate  Std.Err  z-value  P(>|z|)   Std.lv  Std.all
##   Conflict =~
##     NRI_SPV           1.000                               0.954    0.866
##     NRI_SPV (.p2.)    0.956    0.050   19.185    0.000    0.912    0.858
##     NRI_SPV (.p3.)    0.961    0.047   20.348    0.000    0.917    0.875
##   Antagonistic =~
##     NRI_SPV           1.000                               0.939    0.813
##     NRI_SPV (.p5.)    1.091    0.067   16.283    0.000    1.025    0.820
##     NRI_SPV (.p6.)    0.957    0.069   13.896    0.000    0.899    0.814
##   Affection =~
##     NRI_SPV           1.000                               0.771    0.865
##     NRI_SPV (.p8.)    0.991    0.037   27.093    0.000    0.764    0.863
##     NRI_SPV (.p9.)    1.158    0.070   16.534    0.000    0.892    0.849
##   Reassurance =~
##     NRI_SPV           1.000                               1.062    0.904
##     NRI_SPV (.11.)    0.856    0.051   16.656    0.000    0.909    0.830
##     NRI_SPV (.12.)    0.714    0.045   15.928    0.000    0.758    0.758
##   Satisfaction =~
##     NRI_SPV           1.000                               1.084    0.960
##     NRI_SPV (.14.)    0.962    0.038   25.029    0.000    1.043    0.919
##     NRI_SPV (.15.)    0.975    0.036   27.238    0.000    1.057    0.929
##
## Covariances:
##                    Estimate  Std.Err  z-value  P(>|z|)   Std.lv  Std.all
##  .NRI_SPV_Aff_7 ~~
##    .NRI_SPV_Aff_8     0.140    0.034    4.139    0.000    0.140    0.701
##    .NRI_SPV_RS_11    -0.055    0.019   -2.855    0.004   -0.055   -0.201
```

```
##   Conflict ~~
##     Antagonistic      0.839    0.118    7.108    0.000    0.936    0.936
##     Affection        -0.193    0.072   -2.666    0.008   -0.263   -0.263
##     Reassurance      -0.436    0.103   -4.230    0.000   -0.430   -0.430
##     Satisfaction     -0.504    0.104   -4.855    0.000   -0.487   -0.487
##   Antagonistic ~~
##     Affection        -0.235    0.074   -3.156    0.002   -0.325   -0.325
##     Reassurance      -0.459    0.105   -4.361    0.000   -0.460   -0.460
##     Satisfaction     -0.523    0.106   -4.925    0.000   -0.514   -0.514
##   Affection ~~
##     Reassurance       0.721    0.103    7.019    0.000    0.880    0.880
##     Satisfaction      0.664    0.098    6.782    0.000    0.795    0.795
##   Reassurance ~~
##     Satisfaction      0.918    0.132    6.936    0.000    0.796    0.796
##
## Intercepts:
##                    Estimate  Std.Err  z-value  P(>|z|)   Std.lv  Std.all
##    .NRI_SPV_Con_1     2.507    0.092   27.132    0.000    2.507    2.277
##    .NRI_SPV_Con_2     2.331    0.089   26.117    0.000    2.331    2.192
##    .NRI_SPV_Con_3     2.345    0.088   26.668    0.000    2.345    2.238
##    .NRI_SPV_Ant_4     2.606    0.097   26.883    0.000    2.606    2.256
##    .NRI_SPV_Ant_5     2.768    0.105   26.384    0.000    2.768    2.214
##    .NRI_SPV_Ant_6     2.394    0.093   25.840    0.000    2.394    2.168
##    .NRI_SPV_Aff_7     4.613    0.075   61.678    0.000    4.613    5.176
##    .NRI_SPV_Aff_8     4.592    0.074   61.807    0.000    4.592    5.187
##    .NRI_SPV_Aff_9     4.225    0.088   47.914    0.000    4.225    4.021
##    .NRI_SPV_RS_10     3.923    0.099   39.754    0.000    3.923    3.336
##    .NRI_SPV_RS_11     3.866    0.092   42.080    0.000    3.866    3.531
##    .NRI_SPV_RS_12     3.859    0.084   45.986    0.000    3.859    3.859
##    .NRI_SPV_Sat_13    3.845    0.095   40.586    0.000    3.845    3.406
##    .NRI_SPV_Sat_14    3.732    0.095   39.165    0.000    3.732    3.287
##    .NRI_SPV_Sat_15    3.803    0.095   39.823    0.000    3.803    3.342
##
## Variances:
##                    Estimate  Std.Err  z-value  P(>|z|)   Std.lv  Std.all
##    .NRI_SPV_Con_1     0.302    0.048    6.361    0.000    0.302    0.249
##    .NRI_SPV_Con_2     0.299    0.046    6.517    0.000    0.299    0.264
##    .NRI_SPV_Con_3     0.257    0.042    6.176    0.000    0.257    0.234
##    .NRI_SPV_Ant_4     0.452    0.067    6.750    0.000    0.452    0.339
##    .NRI_SPV_Ant_5     0.512    0.077    6.662    0.000    0.512    0.327
##    .NRI_SPV_Ant_6     0.411    0.061    6.720    0.000    0.411    0.337
##    .NRI_SPV_Aff_7     0.200    0.037    5.381    0.000    0.200    0.252
##    .NRI_SPV_Aff_8     0.200    0.037    5.455    0.000    0.200    0.256
##    .NRI_SPV_Aff_9     0.308    0.051    6.035    0.000    0.308    0.279
##    .NRI_SPV_RS_10     0.254    0.051    4.998    0.000    0.254    0.183
##    .NRI_SPV_RS_11     0.372    0.056    6.689    0.000    0.372    0.311
##    .NRI_SPV_RS_12     0.425    0.057    7.448    0.000    0.425    0.425
##    .NRI_SPV_Sat_13    0.099    0.023    4.287    0.000    0.099    0.078
##    .NRI_SPV_Sat_14    0.201    0.031    6.497    0.000    0.201    0.156
##    .NRI_SPV_Sat_15    0.178    0.029    6.146    0.000    0.178    0.137
##     Conflict          0.910    0.131    6.933    0.000    1.000    1.000
##     Antagonistic      0.882    0.140    6.281    0.000    1.000    1.000
##     Affection         0.594    0.091    6.533    0.000    1.000    1.000
##     Reassurance       1.129    0.160    7.040    0.000    1.000    1.000
```

```
##     Satisfaction      1.175    0.150    7.819    0.000    1.000    1.000
```

Compare configural and metric models

Metric invariance can be rejected if the change in $CFI \geq -.010$ supplemented with the change in $RMSEA \geq .015$ or with the change in $SRMR \geq .030$ (Chen, 2007).

```
Comparison1Like<-compareFit(cfa.model, cfa.metric)
summary(Comparison1Like)
```

```
## ################### Nested Model Comparison #########################
##
## Chi-Squared Difference Test
##
##             Df    AIC    BIC  Chisq Chisq diff    RMSEA Df diff Pr(>Chisq)
## cfa.model  156 8387.7 8796.2 247.91
## cfa.metric 166 8390.1 8762.8 270.29     22.382 0.096488      10    0.01327 *
## ---
## Signif. codes:  0 '***' 0.001 '**' 0.01 '*' 0.05 '.' 0.1 ' ' 1
##
## ####################### Model Fit Indices ###########################
##               chisq  df pvalue rmsea   cfi   tli  srmr       aic       bic
## cfa.model  247.908† 156   .000 .067† .973† .964† .041† 8387.721† 8796.239
## cfa.metric 270.291  166   .000 .069  .970  .962  .055  8390.103  8762.787†
##
## ################## Differences in Fit Indices #######################
##                          df rmsea    cfi    tli  srmr   aic     bic
## cfa.metric - cfa.model 10 0.002 -0.004 -0.002 0.014 2.382 -33.453
```

Run a MGCFA for NRI-SPV: Scalar Model

```
cfa.scalar <- cfa(model_NRI, data=datafile,
                  estimator = "ML", group = "Region",

                  group.equal = c("loadings", "intercepts"))
```

Print summary of MGCFA for NRI-SPV: Scalar Model

We used criteria of $CFI > .90$, $RMSEA < .08$, and $SRMR < .08$, which indicate a satisfactory model fit (Hu & Bentler, 1999; Kenny et al., 2015; MacCallum et al., 1996).

```
summary(cfa.scalar , fit.measures = TRUE, standardized = TRUE, ci = FALSE)
```

```
## lavaan 0.6.17 ended normally after 124 iterations
##
##   Estimator                                         ML
##   Optimization method                           NLMINB
##   Number of model parameters                       119
##   Number of equality constraints                    25
```

```
##
##   Number of observations per group:
##     HK                                               124
##     US                                               142
##
## Model Test User Model:
##
##   Test statistic                                 333.885
##   Degrees of freedom                                 176
##   P-value (Chi-square)                             0.000
##   Test statistic for each group:
##     HK                                           162.183
##     US                                           171.702
##
## Model Test Baseline Model:
##
##   Test statistic                                3641.528
##   Degrees of freedom                                 210
##   P-value                                          0.000
##
## User Model versus Baseline Model:
##
##   Comparative Fit Index (CFI)                      0.954
##   Tucker-Lewis Index (TLI)                         0.945
##
## Loglikelihood and Information Criteria:
##
##   Loglikelihood user model (H0)                -4122.849
##   Loglikelihood unrestricted model (H1)        -3955.906
##
##   Akaike (AIC)                                  8433.698
##   Bayesian (BIC)                                8770.547
##   Sample-size adjusted Bayesian (SABIC)         8472.514
##
## Root Mean Square Error of Approximation:
##
##   RMSEA                                            0.082
##   90 Percent confidence interval - lower           0.069
##   90 Percent confidence interval - upper           0.095
##   P-value H_0: RMSEA <= 0.050                      0.000
##   P-value H_0: RMSEA >= 0.080                      0.613
##
## Standardized Root Mean Square Residual:
##
##   SRMR                                             0.063
##
## Parameter Estimates:
##
##   Standard errors                               Standard
##   Information                                   Expected
##   Information saturated (h1) model            Structured
##
##
## Group 1 [HK]:
```

```
##
## Latent Variables:
##                    Estimate  Std.Err  z-value  P(>|z|)   Std.lv  Std.all
##   Conflict =~
##     NRI_SPV           1.000                               0.874    0.901
##     NRI_SPV (.p2.)    0.957    0.050   19.166    0.000    0.836    0.861
##     NRI_SPV (.p3.)    0.963    0.047   20.334    0.000    0.842    0.895
##   Antagonistic =~
##     NRI_SPV           1.000                               0.805    0.813
##     NRI_SPV (.p5.)    1.080    0.068   15.994    0.000    0.869    0.895
##     NRI_SPV (.p6.)    0.971    0.072   13.534    0.000    0.781    0.671
##   Affection =~
##     NRI_SPV           1.000                               0.803    0.883
##     NRI_SPV (.p8.)    1.028    0.036   28.913    0.000    0.826    0.845
##     NRI_SPV (.p9.)    1.118    0.062   18.008    0.000    0.898    0.804
##   Reassurance =~
##     NRI_SPV           1.000                               0.944    0.884
##     NRI_SPV (.11.)    0.890    0.052   17.152    0.000    0.841    0.758
##     NRI_SPV (.12.)    0.754    0.046   16.418    0.000    0.711    0.801
##   Satisfaction =~
##     NRI_SPV           1.000                               0.849    0.928
##     NRI_SPV (.14.)    0.966    0.038   25.423    0.000    0.820    0.810
##     NRI_SPV (.15.)    0.975    0.035   27.623    0.000    0.828    0.889
##
## Covariances:
##                    Estimate  Std.Err  z-value  P(>|z|)   Std.lv  Std.all
##  .NRI_SPV_Aff_7 ~~
##    .NRI_SPV_Aff_8    -0.036    0.039   -0.933    0.351   -0.036   -0.162
##    .NRI_SPV_RS_11    -0.058    0.038   -1.508    0.131   -0.058   -0.187
##   Conflict ~~
##     Antagonistic      0.657    0.098    6.731    0.000    0.935    0.935
##     Affection        -0.131    0.070   -1.860    0.063   -0.187   -0.187
##     Reassurance      -0.289    0.087   -3.311    0.001   -0.351   -0.351
##     Satisfaction     -0.356    0.080   -4.458    0.000   -0.480   -0.480
##   Antagonistic ~~
##     Affection        -0.103    0.066   -1.560    0.119   -0.160   -0.160
##     Reassurance      -0.287    0.084   -3.434    0.001   -0.378   -0.378
##     Satisfaction     -0.344    0.077   -4.487    0.000   -0.503   -0.503
##   Affection ~~
##     Reassurance       0.659    0.099    6.664    0.000    0.869    0.869
##     Satisfaction      0.465    0.080    5.822    0.000    0.682    0.682
##   Reassurance ~~
##     Satisfaction      0.588    0.097    6.034    0.000    0.734    0.734
##
## Intercepts:
##                    Estimate  Std.Err  z-value  P(>|z|)   Std.lv  Std.all
##    .NRI_SPV (.48.)    2.556    0.085   30.096    0.000    2.556    2.636
##    .NRI_SPV (.49.)    2.402    0.083   28.862    0.000    2.402    2.474
##    .NRI_SPV (.50.)    2.436    0.082   29.680    0.000    2.436    2.590
##    .NRI_SPV (.51.)    2.692    0.084   31.918    0.000    2.692    2.719
##    .NRI_SPV (.52.)    2.710    0.086   31.568    0.000    2.710    2.791
##    .NRI_SPV (.53.)    2.662    0.089   30.077    0.000    2.662    2.285
##    .NRI_SPV (.54.)    3.903    0.078   50.086    0.000    3.903    4.291
##    .NRI_SPV (.55.)    3.828    0.081   47.279    0.000    3.828    3.916
```

```
##    .NRI_SPV (.56.)    3.477    0.094   36.972    0.000    3.477    3.113
##    .NRI_SPV (.57.)    3.509    0.093   37.541    0.000    3.509    3.288
##    .NRI_SPV (.58.)    3.414    0.090   38.051    0.000    3.414    3.080
##    .NRI_SPV (.59.)    3.399    0.075   45.226    0.000    3.399    3.827
##    .NRI_SPV (.60.)    3.526    0.081   43.726    0.000    3.526    3.852
##    .NRI_SPV (.61.)    3.409    0.082   41.461    0.000    3.409    3.365
##    .NRI_SPV (.62.)    3.490    0.080   43.436    0.000    3.490    3.748
##
## Variances:
##                    Estimate  Std.Err  z-value  P(>|z|)   Std.lv  Std.all
##    .NRI_SPV_Con_1     0.177    0.032    5.480    0.000    0.177    0.188
##    .NRI_SPV_Con_2     0.243    0.039    6.305    0.000    0.243    0.258
##    .NRI_SPV_Con_3     0.176    0.031    5.648    0.000    0.176    0.199
##    .NRI_SPV_Ant_4     0.332    0.051    6.511    0.000    0.332    0.339
##    .NRI_SPV_Ant_5     0.187    0.039    4.797    0.000    0.187    0.198
##    .NRI_SPV_Ant_6     0.747    0.102    7.331    0.000    0.747    0.550
##    .NRI_SPV_Aff_7     0.182    0.044    4.129    0.000    0.182    0.220
##    .NRI_SPV_Aff_8     0.274    0.055    4.936    0.000    0.274    0.286
##    .NRI_SPV_Aff_9     0.440    0.068    6.464    0.000    0.440    0.353
##    .NRI_SPV_RS_10     0.248    0.050    4.945    0.000    0.248    0.218
##    .NRI_SPV_RS_11     0.522    0.077    6.764    0.000    0.522    0.425
##    .NRI_SPV_RS_12     0.283    0.044    6.492    0.000    0.283    0.359
##    .NRI_SPV_Sat_13    0.117    0.028    4.156    0.000    0.117    0.139
##    .NRI_SPV_Sat_14    0.354    0.052    6.759    0.000    0.354    0.345
##    .NRI_SPV_Sat_15    0.181    0.033    5.494    0.000    0.181    0.209
##     Conflict          0.763    0.113    6.729    0.000    1.000    1.000
##     Antagonistic      0.648    0.109    5.948    0.000    1.000    1.000
##     Affection         0.645    0.097    6.628    0.000    1.000    1.000
##     Reassurance       0.891    0.138    6.477    0.000    1.000    1.000
##     Satisfaction      0.721    0.102    7.061    0.000    1.000    1.000
##
##
## Group 2 [US]:
##
## Latent Variables:
##                    Estimate  Std.Err  z-value  P(>|z|)   Std.lv  Std.all
##   Conflict =~
##     NRI_SPV           1.000                               0.953    0.865
##     NRI_SPV (.p2.)    0.957    0.050   19.166    0.000    0.912    0.858
##     NRI_SPV (.p3.)    0.963    0.047   20.334    0.000    0.918    0.875
##   Antagonistic =~
##     NRI_SPV           1.000                               0.935    0.811
##     NRI_SPV (.p5.)    1.080    0.068   15.994    0.000    1.010    0.805
##     NRI_SPV (.p6.)    0.971    0.072   13.534    0.000    0.908    0.806
##   Affection =~
##     NRI_SPV           1.000                               0.768    0.866
##     NRI_SPV (.p8.)    1.028    0.036   28.913    0.000    0.789    0.871
##     NRI_SPV (.p9.)    1.118    0.062   18.008    0.000    0.858    0.831
##   Reassurance =~
##     NRI_SPV           1.000                               1.042    0.894
##     NRI_SPV (.11.)    0.890    0.052   17.152    0.000    0.928    0.838
##     NRI_SPV (.12.)    0.754    0.046   16.418    0.000    0.785    0.767
##   Satisfaction =~
##     NRI_SPV           1.000                               1.083    0.960
```

```
##     NRI_SPV (.14.)    0.966    0.038   25.423    0.000    1.046    0.919
##     NRI_SPV (.15.)    0.975    0.035   27.623    0.000    1.056    0.929
##
## Covariances:
##                    Estimate  Std.Err  z-value  P(>|z|)   Std.lv  Std.all
##  .NRI_SPV_Aff_7 ~~
##    .NRI_SPV_Aff_8     0.137    0.035    3.892    0.000    0.137    0.693
##    .NRI_SPV_RS_11    -0.055    0.019   -2.877    0.004   -0.055   -0.207
##   Conflict ~~
##     Antagonistic      0.838    0.118    7.110    0.000    0.940    0.940
##     Affection        -0.193    0.072   -2.671    0.008   -0.264   -0.264
##     Reassurance      -0.429    0.101   -4.242    0.000   -0.432   -0.432
##     Satisfaction     -0.503    0.104   -4.855    0.000   -0.488   -0.488
##   Antagonistic ~~
##     Affection        -0.235    0.074   -3.161    0.002   -0.327   -0.327
##     Reassurance      -0.456    0.104   -4.398    0.000   -0.468   -0.468
##     Satisfaction     -0.522    0.106   -4.922    0.000   -0.516   -0.516
##   Affection ~~
##     Reassurance       0.705    0.100    7.028    0.000    0.881    0.881
##     Satisfaction      0.660    0.097    6.797    0.000    0.794    0.794
##   Reassurance ~~
##     Satisfaction      0.902    0.130    6.932    0.000    0.799    0.799
##
## Intercepts:
##                    Estimate  Std.Err  z-value  P(>|z|)   Std.lv  Std.all
##    .NRI_SPV (.48.)    2.556    0.085   30.096    0.000    2.556    2.322
##    .NRI_SPV (.49.)    2.402    0.083   28.862    0.000    2.402    2.259
##    .NRI_SPV (.50.)    2.436    0.082   29.680    0.000    2.436    2.323
##    .NRI_SPV (.51.)    2.692    0.084   31.918    0.000    2.692    2.335
##    .NRI_SPV (.52.)    2.710    0.086   31.568    0.000    2.710    2.159
##    .NRI_SPV (.53.)    2.662    0.089   30.077    0.000    2.662    2.363
##    .NRI_SPV (.54.)    3.903    0.078   50.086    0.000    3.903    4.401
##    .NRI_SPV (.55.)    3.828    0.081   47.279    0.000    3.828    4.227
##    .NRI_SPV (.56.)    3.477    0.094   36.972    0.000    3.477    3.368
##    .NRI_SPV (.57.)    3.509    0.093   37.541    0.000    3.509    3.012
##    .NRI_SPV (.58.)    3.414    0.090   38.051    0.000    3.414    3.085
##    .NRI_SPV (.59.)    3.399    0.075   45.226    0.000    3.399    3.319
##    .NRI_SPV (.60.)    3.526    0.081   43.726    0.000    3.526    3.126
##    .NRI_SPV (.61.)    3.409    0.082   41.461    0.000    3.409    2.996
##    .NRI_SPV (.62.)    3.490    0.080   43.436    0.000    3.490    3.068
##     Conflct          -0.073    0.118   -0.623    0.533   -0.077   -0.077
##     Antgnst          -0.103    0.116   -0.889    0.374   -0.110   -0.110
##     Affectn           0.707    0.105    6.761    0.000    0.920    0.920
##     Ressrnc           0.475    0.130    3.638    0.000    0.456    0.456
##     Stsfctn           0.323    0.122    2.641    0.008    0.298    0.298
##
## Variances:
##                    Estimate  Std.Err  z-value  P(>|z|)   Std.lv  Std.all
##    .NRI_SPV_Con_1     0.304    0.048    6.374    0.000    0.304    0.251
##    .NRI_SPV_Con_2     0.299    0.046    6.516    0.000    0.299    0.265
##    .NRI_SPV_Con_3     0.257    0.042    6.165    0.000    0.257    0.234
##    .NRI_SPV_Ant_4     0.454    0.068    6.726    0.000    0.454    0.342
##    .NRI_SPV_Ant_5     0.555    0.081    6.817    0.000    0.555    0.352
##    .NRI_SPV_Ant_6     0.445    0.066    6.780    0.000    0.445    0.350
```

```
##    .NRI_SPV_Aff_7     0.197    0.038    5.208    0.000    0.197    0.250
##    .NRI_SPV_Aff_8     0.198    0.038    5.137    0.000    0.198    0.241
##    .NRI_SPV_Aff_9     0.329    0.052    6.352    0.000    0.329    0.309
##    .NRI_SPV_RS_10     0.273    0.051    5.331    0.000    0.273    0.201
##    .NRI_SPV_RS_11     0.365    0.056    6.557    0.000    0.365    0.298
##    .NRI_SPV_RS_12     0.432    0.059    7.381    0.000    0.432    0.412
##    .NRI_SPV_Sat_13    0.099    0.023    4.305    0.000    0.099    0.078
##    .NRI_SPV_Sat_14    0.200    0.031    6.474    0.000    0.200    0.155
##    .NRI_SPV_Sat_15    0.178    0.029    6.159    0.000    0.178    0.138
##     Conflict          0.908    0.131    6.927    0.000    1.000    1.000
##     Antagonistic      0.875    0.140    6.241    0.000    1.000    1.000
##     Affection         0.589    0.089    6.599    0.000    1.000    1.000
##     Reassurance       1.085    0.155    6.979    0.000    1.000    1.000
##     Satisfaction      1.173    0.150    7.825    0.000    1.000    1.000
```

Compare metric and scalar models

Scalar invariance can be rejected if the change in $CFI \geq -.010$ supplemented with the change in $RMSEA \geq .015$ or with the change in $SRMR \geq .010$ (Chen, 2007).

```
Comparison2Like<-compareFit(cfa.metric, cfa.scalar)
summary(Comparison2Like)
```

```
## ################### Nested Model Comparison #########################
##
## Chi-Squared Difference Test
##
##             Df    AIC    BIC  Chisq Chisq diff   RMSEA Df diff Pr(>Chisq)
## cfa.metric 166 8390.1 8762.8 270.29
## cfa.scalar 176 8433.7 8770.5 333.89     63.595 0.20074      10   7.52e-10 ***
## ---
## Signif. codes:  0 '***' 0.001 '**' 0.01 '*' 0.05 '.' 0.1 ' ' 1
##
## ####################### Model Fit Indices ###########################
##               chisq  df pvalue rmsea   cfi   tli  srmr       aic       bic
## cfa.metric 270.291† 166   .000 .069† .970† .962† .055† 8390.103† 8762.787†
## cfa.scalar 333.885  176   .000 .082  .954  .945  .063  8433.698  8770.547
##
## ################## Differences in Fit Indices #######################
##                         df rmsea    cfi    tli  srmr    aic  bic
## cfa.scalar - cfa.metric 10 0.013 -0.016 -0.016 0.008 43.595 7.76
```

Run a MGCFA for NRI-SPV: Residual Model

```
cfa.residual <- cfa(model_NRI, data=datafile,
                    estimator = "ML", group = "Region",

                    group.equal = c("loadings", "intercepts", "residuals"))
```

Print summary of MGCFA for NRI-SPV: Residual Model

We used criteria of $CFI > .90$, $RMSEA < .08$, and $SRMR < .08$, which indicate a satisfactory model fit (Hu & Bentler, 1999; Kenny et al., 2015; MacCallum et al., 1996).

```r
summary(cfa.residual, fit.measures = TRUE, standardized = TRUE, ci = FALSE)
```

```
## lavaan 0.6.17 ended normally after 106 iterations
##
##   Estimator                                         ML
##   Optimization method                           NLMINB
##   Number of model parameters                       119
##   Number of equality constraints                    40
##
##   Number of observations per group:
##     HK                                             124
##     US                                             142
##
## Model Test User Model:
##
##   Test statistic                               384.361
##   Degrees of freedom                               191
##   P-value (Chi-square)                           0.000
##   Test statistic for each group:
##     HK                                         186.668
##     US                                         197.693
##
## Model Test Baseline Model:
##
##   Test statistic                              3641.528
##   Degrees of freedom                               210
##   P-value                                        0.000
##
## User Model versus Baseline Model:
##
##   Comparative Fit Index (CFI)                    0.944
##   Tucker-Lewis Index (TLI)                       0.938
##
## Loglikelihood and Information Criteria:
##
##   Loglikelihood user model (H0)              -4148.087
##   Loglikelihood unrestricted model (H1)      -3955.906
##
##   Akaike (AIC)                                8454.173
##   Bayesian (BIC)                              8737.269
##   Sample-size adjusted Bayesian (SABIC)       8486.795
##
## Root Mean Square Error of Approximation:
##
##   RMSEA                                          0.087
##   90 Percent confidence interval - lower         0.075
##   90 Percent confidence interval - upper         0.100
##   P-value H_0: RMSEA <= 0.050                    0.000
##   P-value H_0: RMSEA >= 0.080                    0.832
```

```
##
## Standardized Root Mean Square Residual:
##
##   SRMR                                           0.061
##
## Parameter Estimates:
##
##   Standard errors                             Standard
##   Information                                 Expected
##   Information saturated (h1) model          Structured
##
##
## Group 1 [HK]:
##
## Latent Variables:
##                    Estimate  Std.Err  z-value  P(>|z|)   Std.lv  Std.all
##   Conflict =~
##     NRI_SPV           1.000                               0.870    0.872
##     NRI_SPV (.p2.)    0.952    0.050   19.148    0.000    0.828    0.847
##     NRI_SPV (.p3.)    0.949    0.048   19.869    0.000    0.825    0.865
##   Antagonistic =~
##     NRI_SPV           1.000                               0.786    0.785
##     NRI_SPV (.p5.)    1.054    0.066   15.998    0.000    0.829    0.814
##     NRI_SPV (.p6.)    0.927    0.071   13.020    0.000    0.729    0.683
##   Affection =~
##     NRI_SPV           1.000                               0.805    0.872
##     NRI_SPV (.p8.)    1.034    0.036   28.807    0.000    0.832    0.869
##     NRI_SPV (.p9.)    1.096    0.062   17.700    0.000    0.883    0.815
##   Reassurance =~
##     NRI_SPV           1.000                               0.940    0.877
##     NRI_SPV (.11.)    0.891    0.053   16.967    0.000    0.837    0.786
##     NRI_SPV (.12.)    0.754    0.046   16.235    0.000    0.709    0.761
##   Satisfaction =~
##     NRI_SPV           1.000                               0.851    0.932
##     NRI_SPV (.14.)    0.952    0.040   23.785    0.000    0.810    0.840
##     NRI_SPV (.15.)    0.976    0.036   27.371    0.000    0.831    0.892
##
## Covariances:
##                    Estimate  Std.Err  z-value  P(>|z|)   Std.lv  Std.all
##  .NRI_SPV_Aff_7 ~~
##    .NRI_SPV_Aff_8    -0.042    0.034   -1.229    0.219   -0.042   -0.194
##    .NRI_SPV_RS_11    -0.061    0.037   -1.673    0.094   -0.061   -0.206
##   Conflict ~~
##     Antagonistic      0.662    0.098    6.746    0.000    0.967    0.967
##     Affection        -0.125    0.071   -1.769    0.077   -0.179   -0.179
##     Reassurance      -0.289    0.088   -3.288    0.001   -0.354   -0.354
##     Satisfaction     -0.355    0.080   -4.418    0.000   -0.479   -0.479
##   Antagonistic ~~
##     Affection        -0.103    0.067   -1.548    0.122   -0.163   -0.163
##     Reassurance      -0.290    0.084   -3.450    0.001   -0.393   -0.393
##     Satisfaction     -0.346    0.077   -4.493    0.000   -0.517   -0.517
##   Affection ~~
##     Reassurance       0.660    0.099    6.662    0.000    0.872    0.872
##     Satisfaction      0.467    0.080    5.840    0.000    0.682    0.682
```

```
##   Reassurance ~~
##     Satisfaction      0.585    0.097    6.010    0.000    0.730    0.730
##
## Intercepts:
##                    Estimate  Std.Err  z-value  P(>|z|)   Std.lv  Std.all
##    .NRI_SPV (.48.)    2.561    0.086   29.839    0.000    2.561    2.569
##    .NRI_SPV (.49.)    2.402    0.083   28.951    0.000    2.402    2.457
##    .NRI_SPV (.50.)    2.432    0.082   29.719    0.000    2.432    2.549
##    .NRI_SPV (.51.)    2.692    0.084   32.089    0.000    2.692    2.687
##    .NRI_SPV (.52.)    2.767    0.087   31.915    0.000    2.767    2.719
##    .NRI_SPV (.53.)    2.718    0.084   32.273    0.000    2.718    2.547
##    .NRI_SPV (.54.)    3.893    0.078   49.784    0.000    3.893    4.219
##    .NRI_SPV (.55.)    3.811    0.081   47.129    0.000    3.811    3.980
##    .NRI_SPV (.56.)    3.487    0.092   37.841    0.000    3.487    3.221
##    .NRI_SPV (.57.)    3.507    0.094   37.483    0.000    3.507    3.272
##    .NRI_SPV (.58.)    3.410    0.089   38.503    0.000    3.410    3.202
##    .NRI_SPV (.59.)    3.419    0.076   44.740    0.000    3.419    3.670
##    .NRI_SPV (.60.)    3.526    0.081   43.724    0.000    3.526    3.862
##    .NRI_SPV (.61.)    3.407    0.081   42.022    0.000    3.407    3.531
##    .NRI_SPV (.62.)    3.489    0.081   43.325    0.000    3.489    3.744
##
## Variances:
##                    Estimate  Std.Err  z-value  P(>|z|)   Std.lv  Std.all
##    .NRI_SPV (.18.)    0.237    0.029    8.135    0.000    0.237    0.239
##    .NRI_SPV (.19.)    0.270    0.031    8.843    0.000    0.270    0.283
##    .NRI_SPV (.20.)    0.229    0.027    8.360    0.000    0.229    0.251
##    .NRI_SPV (.21.)    0.386    0.043    9.021    0.000    0.386    0.384
##    .NRI_SPV (.22.)    0.349    0.042    8.378    0.000    0.349    0.337
##    .NRI_SPV (.23.)    0.607    0.059   10.214    0.000    0.607    0.533
##    .NRI_SPV (.24.)    0.203    0.029    6.916    0.000    0.203    0.239
##    .NRI_SPV (.25.)    0.225    0.032    7.093    0.000    0.225    0.245
##    .NRI_SPV (.26.)    0.393    0.043    9.208    0.000    0.393    0.336
##    .NRI_SPV (.27.)    0.265    0.037    7.150    0.000    0.265    0.231
##    .NRI_SPV (.28.)    0.433    0.046    9.360    0.000    0.433    0.382
##    .NRI_SPV (.29.)    0.366    0.037    9.825    0.000    0.366    0.421
##    .NRI_SPV (.30.)    0.109    0.019    5.784    0.000    0.109    0.131
##    .NRI_SPV (.31.)    0.274    0.029    9.469    0.000    0.274    0.295
##    .NRI_SPV (.32.)    0.178    0.022    7.979    0.000    0.178    0.205
##     Conflct           0.756    0.115    6.562    0.000    1.000    1.000
##     Antgnst           0.618    0.107    5.787    0.000    1.000    1.000
##     Affectn           0.648    0.097    6.706    0.000    1.000    1.000
##     Ressrnc           0.884    0.137    6.439    0.000    1.000    1.000
##     Stsfctn           0.725    0.102    7.104    0.000    1.000    1.000
##
##
## Group 2 [US]:
##
## Latent Variables:
##                    Estimate  Std.Err  z-value  P(>|z|)   Std.lv  Std.all
##   Conflict =~
##     NRI_SPV           1.000                               0.968    0.893
##     NRI_SPV (.p2.)    0.952    0.050   19.148    0.000    0.921    0.871
##     NRI_SPV (.p3.)    0.949    0.048   19.869    0.000    0.919    0.887
##   Antagonistic =~
```

```
##     NRI_SPV           1.000                               0.969    0.842
##     NRI_SPV (.p5.)    1.054    0.066   15.998    0.000    1.021    0.866
##     NRI_SPV (.p6.)    0.927    0.071   13.020    0.000    0.899    0.756
##   Affection =~
##     NRI_SPV           1.000                               0.761    0.860
##     NRI_SPV (.p8.)    1.034    0.036   28.807    0.000    0.786    0.856
##     NRI_SPV (.p9.)    1.096    0.062   17.700    0.000    0.834    0.799
##   Reassurance =~
##     NRI_SPV           1.000                               1.041    0.896
##     NRI_SPV (.11.)    0.891    0.053   16.967    0.000    0.927    0.815
##     NRI_SPV (.12.)    0.754    0.046   16.235    0.000    0.784    0.792
##   Satisfaction =~
##     NRI_SPV           1.000                               1.082    0.956
##     NRI_SPV (.14.)    0.952    0.040   23.785    0.000    1.029    0.891
##     NRI_SPV (.15.)    0.976    0.036   27.371    0.000    1.056    0.929
##
## Covariances:
##                    Estimate  Std.Err  z-value  P(>|z|)   Std.lv  Std.all
##  .NRI_SPV_Aff_7 ~~
##    .NRI_SPV_Aff_8     0.152    0.028    5.498    0.000    0.152    0.712
##    .NRI_SPV_RS_11    -0.062    0.020   -3.093    0.002   -0.062   -0.208
##   Conflict ~~
##     Antagonistic      0.842    0.120    7.042    0.000    0.897    0.897
##     Affection        -0.192    0.073   -2.633    0.008   -0.261   -0.261
##     Reassurance      -0.432    0.102   -4.246    0.000   -0.429   -0.429
##     Satisfaction     -0.507    0.104   -4.854    0.000   -0.484   -0.484
##   Antagonistic ~~
##     Affection        -0.249    0.076   -3.263    0.001   -0.338   -0.338
##     Reassurance      -0.464    0.106   -4.388    0.000   -0.460   -0.460
##     Satisfaction     -0.539    0.109   -4.955    0.000   -0.514   -0.514
##   Affection ~~
##     Reassurance       0.704    0.100    7.042    0.000    0.889    0.889
##     Satisfaction      0.661    0.097    6.806    0.000    0.804    0.804
##   Reassurance ~~
##     Satisfaction      0.904    0.130    6.944    0.000    0.803    0.803
##
## Intercepts:
##                    Estimate  Std.Err  z-value  P(>|z|)   Std.lv  Std.all
##    .NRI_SPV (.48.)    2.561    0.086   29.839    0.000    2.561    2.364
##    .NRI_SPV (.49.)    2.402    0.083   28.951    0.000    2.402    2.271
##    .NRI_SPV (.50.)    2.432    0.082   29.719    0.000    2.432    2.347
##    .NRI_SPV (.51.)    2.692    0.084   32.089    0.000    2.692    2.339
##    .NRI_SPV (.52.)    2.767    0.087   31.915    0.000    2.767    2.345
##    .NRI_SPV (.53.)    2.718    0.084   32.273    0.000    2.718    2.285
##    .NRI_SPV (.54.)    3.893    0.078   49.784    0.000    3.893    4.402
##    .NRI_SPV (.55.)    3.811    0.081   47.129    0.000    3.811    4.151
##    .NRI_SPV (.56.)    3.487    0.092   37.841    0.000    3.487    3.342
##    .NRI_SPV (.57.)    3.507    0.094   37.483    0.000    3.507    3.021
##    .NRI_SPV (.58.)    3.410    0.089   38.503    0.000    3.410    3.000
##    .NRI_SPV (.59.)    3.419    0.076   44.740    0.000    3.419    3.451
##    .NRI_SPV (.60.)    3.526    0.081   43.724    0.000    3.526    3.118
##    .NRI_SPV (.61.)    3.407    0.081   42.022    0.000    3.407    2.950
##    .NRI_SPV (.62.)    3.489    0.081   43.325    0.000    3.489    3.069
##     Conflct          -0.073    0.118   -0.614    0.539   -0.075   -0.075
```

```
##     Antgnst          -0.100    0.117   -0.850    0.395   -0.103   -0.103
##     Affectn           0.715    0.105    6.842    0.000    0.940    0.940
##     Ressrnc           0.473    0.130    3.635    0.000    0.455    0.455
##     Stsfctn           0.324    0.122    2.647    0.008    0.299    0.299
##
## Variances:
##                    Estimate  Std.Err  z-value  P(>|z|)   Std.lv  Std.all
##    .NRI_SPV (.18.)    0.237    0.029    8.135    0.000    0.237    0.202
##    .NRI_SPV (.19.)    0.270    0.031    8.843    0.000    0.270    0.241
##    .NRI_SPV (.20.)    0.229    0.027    8.360    0.000    0.229    0.213
##    .NRI_SPV (.21.)    0.386    0.043    9.021    0.000    0.386    0.291
##    .NRI_SPV (.22.)    0.349    0.042    8.378    0.000    0.349    0.250
##    .NRI_SPV (.23.)    0.607    0.059   10.214    0.000    0.607    0.429
##    .NRI_SPV (.24.)    0.203    0.029    6.916    0.000    0.203    0.260
##    .NRI_SPV (.25.)    0.225    0.032    7.093    0.000    0.225    0.267
##    .NRI_SPV (.26.)    0.393    0.043    9.208    0.000    0.393    0.361
##    .NRI_SPV (.27.)    0.265    0.037    7.150    0.000    0.265    0.197
##    .NRI_SPV (.28.)    0.433    0.046    9.360    0.000    0.433    0.335
##    .NRI_SPV (.29.)    0.366    0.037    9.825    0.000    0.366    0.373
##    .NRI_SPV (.30.)    0.109    0.019    5.784    0.000    0.109    0.085
##    .NRI_SPV (.31.)    0.274    0.029    9.469    0.000    0.274    0.206
##    .NRI_SPV (.32.)    0.178    0.022    7.979    0.000    0.178    0.138
##     Conflct           0.937    0.132    7.076    0.000    1.000    1.000
##     Antgnst           0.940    0.146    6.442    0.000    1.000    1.000
##     Affectn           0.579    0.089    6.532    0.000    1.000    1.000
##     Ressrnc           1.083    0.154    7.009    0.000    1.000    1.000
##     Stsfctn           1.170    0.150    7.784    0.000    1.000    1.000
```

Compare scalar and residual models

Residual invariance can be rejected if the change in $CFI \geq -.010$ supplemented with the change in $RMSEA \geq .015$ or with the change in $SRMR \geq .010$ (Chen, 2007).

```
Comparison3Like<-compareFit(cfa.scalar,cfa.residual)
summary(Comparison3Like)
```

```
## ################### Nested Model Comparison #########################
##
## Chi-Squared Difference Test
##
##               Df    AIC    BIC  Chisq Chisq diff   RMSEA Df diff Pr(>Chisq)
## cfa.scalar   176 8433.7 8770.5 333.89
## cfa.residual 191 8454.2 8737.3 384.36     50.475 0.13335      15  1.007e-05 ***
## ---
## Signif. codes:  0 '***' 0.001 '**' 0.01 '*' 0.05 '.' 0.1 ' ' 1
##
## ####################### Model Fit Indices ###########################
##                 chisq  df pvalue rmsea   cfi   tli  srmr       aic       bic
## cfa.scalar   333.885† 176   .000 .082† .954† .945† .063  8433.698† 8770.547
## cfa.residual 384.361  191   .000 .087  .944  .938  .061† 8454.173  8737.269†
##
## ################## Differences in Fit Indices #######################
##                            df rmsea   cfi    tli   srmr    aic     bic
```

```
## cfa.residual - cfa.scalar 15 0.005 -0.01 -0.007 -0.002 20.475 -33.277
```

Compute composite reliability for NRI-SPV

```r
compRelSEM(cfa.scalar,return.total = TRUE)
```

```
##   group Conflict Antagonistic Affection Reassurance Satisfaction total
## 1    HK    0.916        0.888     0.924       0.890        0.941 0.918
## 2    US    0.899        0.852     0.860       0.875        0.951 0.929
```
