# Supplementary material for: How parents leverage guilt and pride: A comparison of parental guilt and pride induction in Hong Kong and the United States
Source: J Res Adolesc. 2025 Dec 10;35(4):e70107. doi: 10.1111/jora.70107 (PMC12696220; doi:10.1111/jora.70107)
Supplement: Supplementary file 6 — Data S5: [file JORA-35-0-s003.pdf]

# Regressions

Corresponding Author

2024-10-25

This R Markdown document presents the code for the regression analyses in our study. Regression analysis was used to test H2 and H3.

H2: The negative association between guilt induction and adolescent-parent relationship quality (including perceived support from parents and negative interaction with parents) is more pronounced in topics perceived as not legitimately regulated by parents (i.e., personal domain).

H3: The negative association between guilt induction and adolescent-parent relationship quality is stronger among US participants.

## Load packages

```
library(interactions)
library(psych)
library(dplyr)
library(tidyr)
library(ggplot2)
```

## Import data

```
datafile <- read.csv("D:\\R WM\\gipca data csv_with newly added data.csv")
```

## Data preparation

(1) Create dummy variable for guilt induction personal domain (yes or no)
(2) Compute means for perceived support from parents (Parent_supp) and negative interaction with parents (Parent_NI).

```
# Select data needed
subset_data_DDPGI <- datafile %>%
  select(
    DDPGI_Moral_Mean, DDPGI_Conven_Mean, DDPGI_Pruden_Mean, DDPGI_Person_Mean,
    Region, Parent_conflict_Mean, Parent_antag_Mean, Parent_affection_Mean,
    Parent_reassurance_Mean, Parent_satisfaction_Mean, Age
  )

# Restack the data using pivot_longer
```

```
long_data <- subset_data_DDPGI %>%
  pivot_longer(
    cols = starts_with("DDPGI_"),  # Select columns that start with "DDPGI_"
    names_to = "Guilt_Induction_Type",
    values_to = "Guilt_Induction_Mean_Score"
  ) %>%

  # Create dummy variable for guilt induction personal domain (yes or no; DDPGI_Recoded)
  mutate(DDPGI_Recoded = ifelse(Guilt_Induction_Type ==
                                "DDPGI_Person_Mean", 1, 0)) #DDPGI_Person_Mean=1

# Compute mean for perceived support from parents (Parent_supp) and negative interaction with parents (
long_data <- long_data %>%
  mutate(
    Parent_NI = rowMeans(select(., Parent_conflict_Mean,
                                Parent_antag_Mean), na.rm = TRUE),
    Parent_supp = rowMeans(select(., Parent_affection_Mean,
                                  Parent_reassurance_Mean,
                                  Parent_satisfaction_Mean), na.rm = TRUE)
  )
```

## Perceived parental support as the outcome

### Parental support ~ DDPGI * personal domain (yes or no) * Culture (HK or US)

```
# Run the regression analysis with interaction term
model_interaction1 <- lm(Parent_supp ~ Guilt_Induction_Mean_Score*
                           DDPGI_Recoded * Region, data = long_data)

summary(model_interaction1)
```

```
##
## Call:
## lm(formula = Parent_supp ~ Guilt_Induction_Mean_Score * DDPGI_Recoded *
##     Region, data = long_data)
##
## Residuals:
##     Min      1Q  Median      3Q     Max
## -2.8453 -0.4495  0.1604  0.5782  1.6629
##
## Coefficients:
##                                                    Estimate Std. Error t value
## (Intercept)                                         3.47005    0.12103  28.672
## Guilt_Induction_Mean_Score                          0.02700    0.04146   0.651
## DDPGI_Recoded                                       0.44627    0.22487   1.985
## RegionUS                                            1.18038    0.19485   6.058
## Guilt_Induction_Mean_Score:DDPGI_Recoded           -0.21392    0.09719  -2.201
## Guilt_Induction_Mean_Score:RegionUS                -0.18801    0.05737  -3.277
## DDPGI_Recoded:RegionUS                              0.19376    0.31891   0.608
## Guilt_Induction_Mean_Score:DDPGI_Recoded:RegionUS -0.11341    0.12093  -0.938
##                                                    Pr(>|t|)
```

```
## (Intercept)                                          < 2e-16 ***
## Guilt_Induction_Mean_Score                           0.51516
## DDPGI_Recoded                                        0.04745 *
## RegionUS                                            1.92e-09 ***
## Guilt_Induction_Mean_Score:DDPGI_Recoded             0.02795 *
## Guilt_Induction_Mean_Score:RegionUS                  0.00108 **
## DDPGI_Recoded:RegionUS                               0.54361
## Guilt_Induction_Mean_Score:DDPGI_Recoded:RegionUS   0.34852
## ---
## Signif. codes:  0 '***' 0.001 '**' 0.01 '*' 0.05 '.' 0.1 ' ' 1
##
## Residual standard error: 0.8077 on 1056 degrees of freedom
## Multiple R-squared:  0.1538, Adjusted R-squared:  0.1482
## F-statistic: 27.42 on 7 and 1056 DF,  p-value: < 2.2e-16
```

**Parental support: Simple slope analysis**

DDPGI * personal domain (yes or no)

```
# Simple slopes analysis
sim_slopes(model_interaction1, pred = Guilt_Induction_Mean_Score,
           modx = DDPGI_Recoded)
```

```
## JOHNSON-NEYMAN INTERVAL
##
## When DDPGI_Recoded is OUTSIDE the interval [-0.89, 0.71], the slope of
## Guilt_Induction_Mean_Score is p < .05.
##
## Note: The range of observed values of DDPGI_Recoded is [0.00, 1.00]
##
## SIMPLE SLOPES ANALYSIS
##
## Slope of Guilt_Induction_Mean_Score when DDPGI_Recoded = 0.00 (0):
##
##   Est.   S.E.   t val.      p
## ------ ------ --------- ------
##   0.03   0.04     0.65   0.52
##
## Slope of Guilt_Induction_Mean_Score when DDPGI_Recoded = 1.00 (1):
##
##    Est.   S.E.   t val.      p
## ------- ------ --------- ------
##   -0.19   0.09    -2.13   0.03
```

**Parental support: Simple slope analysis**

DDPGI * Region

```
# Simple slopes analysis
sim_slopes(model_interaction1, pred = Guilt_Induction_Mean_Score,
           modx = Region)
```

```
## SIMPLE SLOPES ANALYSIS
##
## Slope of Guilt_Induction_Mean_Score when Region = US:
##
##    Est.   S.E.   t val.      p
## -------- ------ -------- ------
##   -0.24   0.03    -7.29   0.00
##
## Slope of Guilt_Induction_Mean_Score when Region = HK:
##
##    Est.   S.E.   t val.      p
## -------- ------ -------- ------
##   -0.03   0.04    -0.70   0.49
```

**Parental support: Interaction plot**

```
# Prepare data for plotting
# Create a sequence of Guilt Induction Mean Scores for plotting
score_seq <- seq(min(long_data$Guilt_Induction_Mean_Score, na.rm = TRUE),
                 max(long_data$Guilt_Induction_Mean_Score, na.rm = TRUE),
                 length.out = 100)

# Create a new data frame for predictions
pred_data <- expand.grid(
  Guilt_Induction_Mean_Score = score_seq,
  DDPGI_Recoded = c(0, 1),  # Assuming 0 and 1 are the two levels
  Region = unique(long_data$Region)  # Include all regions
)

# Get predictions from the model
pred_data$Predicted_Parent_Supp <- predict(model_interaction1, newdata = pred_data)

# Plot the interaction with renamed legend and custom line styles
ggplot(pred_data, aes(x = Guilt_Induction_Mean_Score, y = Predicted_Parent_Supp)) +
  geom_line(aes(linetype = factor(DDPGI_Recoded)),
            color = "Black",  # Set color directly to black
            size = 0.8,
            show.legend = TRUE) +  # Keep linetype legend
  labs(title = "Guilt Induction Mean x Guilt Induction Domain on Support from Parents",
       x = "Guilt Induction Mean Score",
       y = "Perceived Support from Parents",
       linetype = "Domain") +  # Only linetype in legend
  scale_linetype_manual(values = c("0" = "dotted", "1" = "solid"),
                        labels = c("0" = "Legitimately Regulated", "1" = "Personal Issues")) +
  theme_minimal() +
  theme(legend.position = "right") +
  facet_wrap(~ Region)
```

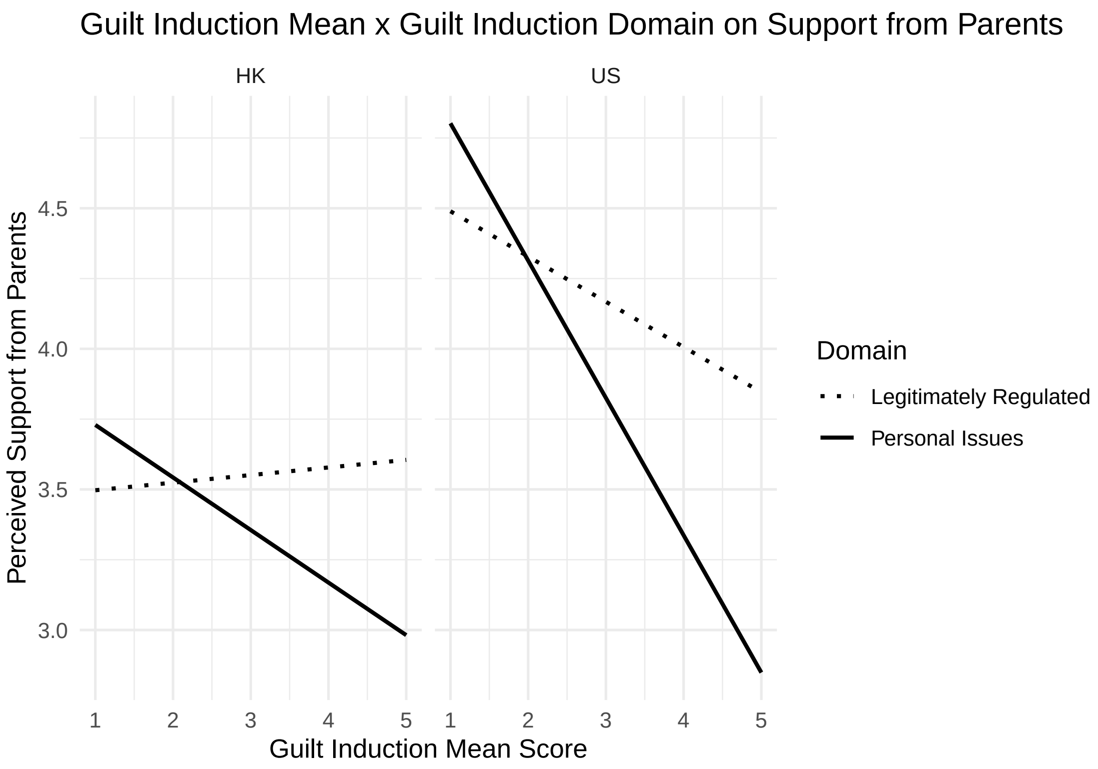


Guilt Induction * Guilt Induction Domain on Support from Parents

## Negative interactions with parents as the outcome

### Negative interactions with parents ~ DDPGI * personal domain (yes or no) * Culture (HK or US)

```
# Run the regression analysis with interaction term
model_interaction2 <- lm(Parent_NI ~ Guilt_Induction_Mean_Score*DDPGI_Recoded
                         * Region, data = long_data)

summary(model_interaction2)
```

```
##
## Call:
## lm(formula = Parent_NI ~ Guilt_Induction_Mean_Score * DDPGI_Recoded *
##     Region, data = long_data)
##
## Residuals:
##      Min       1Q   Median       3Q      Max
## -1.71111 -0.63641 -0.09905  0.56787  2.51168
##
## Coefficients:
##                                                     Estimate Std. Error t value
## (Intercept)                                          2.41229    0.13336  18.088
## Guilt_Induction_Mean_Score                           0.07471    0.04569   1.635
## DDPGI_Recoded                                       -0.38764    0.24779  -1.564
```

```
## RegionUS                                          -0.16114    0.21472  -0.750
## Guilt_Induction_Mean_Score:DDPGI_Recoded           0.22260    0.10710   2.078
## Guilt_Induction_Mean_Score:RegionUS               -0.01008    0.06322  -0.159
## DDPGI_Recoded:RegionUS                            -0.24556    0.35142  -0.699
## Guilt_Induction_Mean_Score:DDPGI_Recoded:RegionUS  0.05705    0.13325   0.428
##                                                   Pr(>|t|)
## (Intercept)                                         <2e-16 ***
## Guilt_Induction_Mean_Score                          0.1023
## DDPGI_Recoded                                       0.1180
## RegionUS                                            0.4531
## Guilt_Induction_Mean_Score:DDPGI_Recoded            0.0379 *
## Guilt_Induction_Mean_Score:RegionUS                 0.8733
## DDPGI_Recoded:RegionUS                              0.4849
## Guilt_Induction_Mean_Score:DDPGI_Recoded:RegionUS   0.6687
## ---
## Signif. codes:  0 '***' 0.001 '**' 0.01 '*' 0.05 '.' 0.1 ' ' 1
##
## Residual standard error: 0.89 on 1056 degrees of freedom
## Multiple R-squared:  0.04226,    Adjusted R-squared:  0.03591
## F-statistic: 6.656 on 7 and 1056 DF,  p-value: 9.987e-08
```

### Negative interactions with parents: Simple slope analysis

DDPGI * personal domain (yes or no)

```
# Simple slopes analysis
sim_slopes(model_interaction2, pred = Guilt_Induction_Mean_Score,
           modx = DDPGI_Recoded)
```

```
## JOHNSON-NEYMAN INTERVAL
##
## When DDPGI_Recoded is OUTSIDE the interval [-9.21, 0.05], the slope of
## Guilt_Induction_Mean_Score is p < .05.
##
## Note: The range of observed values of DDPGI_Recoded is [0.00, 1.00]
##
## SIMPLE SLOPES ANALYSIS
##
## Slope of Guilt_Induction_Mean_Score when DDPGI_Recoded = 0.00 (0):
##
##   Est.   S.E.   t val.      p
## ------ ------ -------- ------
##   0.07   0.05     1.64   0.10
##
## Slope of Guilt_Induction_Mean_Score when DDPGI_Recoded = 1.00 (1):
##
##   Est.   S.E.   t val.      p
## ------ ------ -------- ------
##   0.30   0.10     3.07   0.00
```

**Negative interactions with parents: Interaction plot**

```
# Prepare data for plotting
# Create a sequence of Guilt Induction Mean Scores for plotting
score_seq <- seq(min(long_data$Guilt_Induction_Mean_Score, na.rm = TRUE),
                 max(long_data$Guilt_Induction_Mean_Score, na.rm = TRUE),
                 length.out = 100)

# Create a new data frame for predictions
pred_data <- expand.grid(
  Guilt_Induction_Mean_Score = score_seq,
  DDPGI_Recoded = c(0, 1),  # Assuming 0 and 1 are the two levels
  Region = unique(long_data$Region)  # Include all regions
)

# Get predictions from the model

pred_data$Predicted_Parent_NI <- predict(model_interaction2, newdata = pred_data)
ggplot(pred_data, aes(x = Guilt_Induction_Mean_Score, y = Predicted_Parent_NI)) +
  geom_line(aes(linetype = factor(DDPGI_Recoded)),
            color = "Black",  # Set color directly to black
            size = 0.8,
            show.legend = TRUE) +  # Keep linetype legend
  labs(title = "Guilt Induction Mean x Guilt Induction Domain on
       Negative Interaction with Parents",
       x = "Guilt Induction Mean Score",
       y = "Negative Interaction with Parents",
       linetype = "Domain") +  # Only linetype in legend
  scale_linetype_manual(values = c("0" = "dotted", "1" = "solid"),
                        labels = c("0" = "Legitimately Regulated",
                                   "1" = "Personal Issues")) +
  theme_minimal() +
  theme(legend.position = "right") +
  facet_wrap(~ Region)
```

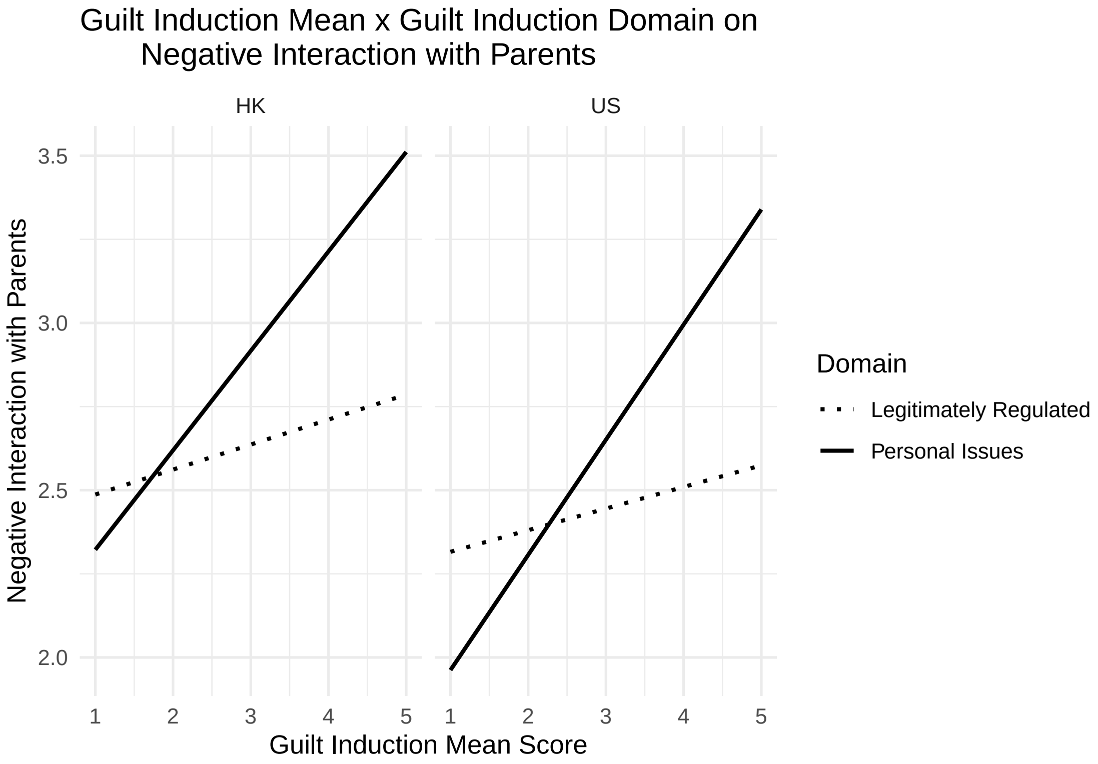


Guilt Induction * Guilt Induction Domain on Negative Interaction with Parents
